# Are there differences in efficacy and safety between local and imported direct-acting antiviral agents for hepatitis C in China?

**DOI:** 10.1186/s40249-025-01344-2

**Published:** 2025-07-25

**Authors:** Zhitao Wang, Youbing Ran, Yihan Fu, Jing Sun, Yuanli Liu

**Affiliations:** School of Health Policy and Management, Chinese Academy of Medical Sciences and Peking Union Medical College, Beijing, China

**Keywords:** Direct-acting antiviral agent, Hepatitis C, Hepatitis C virus, Efficacy, Safety

## Abstract

**Background:**

Despite price advantages, local direct-acting antiviral agents (DAAs) for hepatitis C (hep C) have not been widely used in China compared with the imported ones. There is no evidence on their relative efficacy and safety, nor whether the small market share of local DAAs was attributable to the potential differences.

**Methods:**

This study systematically evaluated the efficacy and safety evidence of 5 local and 6 imported DAAs with valid Chinese registration numbers as of January 25, 2024. Meta-analyses, subgroup analyses and meta-regressions were performed to synthesize evidence and compared the outcomes by using the random-effects empirical Bayes model.

**Results:**

Nineteen randomized controlled trials and 82 single-arm trials (SATs) were included. The results demonstrated no statistically significant difference in 12-week sustained virological response [0.97, (95% confidence interval (*CI*) 0.95, 0.99) vs 0.96, (95% *CI:* 0.94, 0.98), *P* = 0.21], relapse [0.02, (95% *CI:* 0.01, 0.04) vs 0.02, (95% *CI:* 0.01, 0.03), *P* = 0.65], virological breakthrough [0.003, (95% *CI:* < 0.001, 0.02) vs 0.0000002, (95% *CI:* < 0.001, 0.0006), *P* = 0.51] and serious adverse events (SAEs) [0.04, (95% *CI:* 0.03, 0.06) vs 0.03, (95% *CI:* 0.02, 0.03), *P* = 0.12] between local and imported DAAs. By controlling for ethnicities of patients in multiple meta-regression, the local DAAs had a 33.7% higher rate of adverse events (AEs) [0.337, (95% *CI:* 0.188, 0.486), *P* < 0.001]. No statistically significant difference was found in the interaction test between local and imported pan-genotypic DAAs regarding the rate of AEs [0.72, (95% *CI:* 0.64, 0.79) vs 0.73, (95% *CI:* 0.65, 0.50), *P* = 0.81].

**Conclusions:**

Current evidence demonstrates no statistically significant differences in efficacy and SAEs between local and imported DAAs. Given that simplified pan-genotypic DAA regimens are now standard care, local pan-genotypic DAAs hold potential to increase hepatitis C virus treatment rates in China. It is critical for local DAA developers to generate more evidence with expanded patient population in terms of age, treatment experience and genotype of hepatitis C virus, conducting head-to-head studies directly comparing the efficacy and safety. Clinical and policy decision-making should be adaptive and evolve as new evidence is generated.

**Graphical Abstract:**

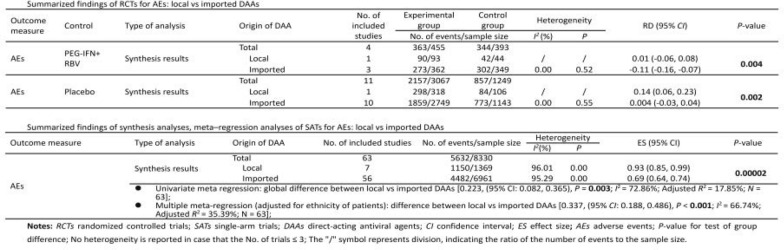

**Supplementary Information:**

The online version contains supplementary material available at 10.1186/s40249-025-01344-2.

## Background

China bears a substantial burden of chronic hepatitis C virus (HCV) infections and associated liver cancer incidence and mortality. In 2024, the prevalent cases of hepatitis C (hep C) in China are 3,938,663, with 21,893 liver-related deaths [1]. Effective control and prevention of HCV in China is crucial for the world to achieve the 2030 global target of eliminating the public health threat of viral hepatitis. China allocated substantial government funding to support local research and development of oral direct-acting antiviral agents (DAAs), followed by the fast-track review and approval for market authorization, as well as swift public funding coverage to enable patient access. Two years after the approval of the first imported DAA, China had its first local DAA marketed [2, 3], marking a significant milestone in local pharmaceutical innovation. By the end of 2023, there were 11 DAAs holding valid registration numbers in China. Among which, all five local and four of six imported DAAs were covered by the national health insurance soon after market authorization (Figure S1). Local DAAs remain largely confined to the Chinese market, except ravidasvir (RDV), which was registered as pan-genotypic in combination with sofosbuvir (SOF) in Malaysia and Egypt. However, RDV is only approved for use in combination with ritonavir-boosted danoprevir (DNV) and ribavirin (RBV) for genotype 1b treatment in China.

Despite a significant price advantage over imported DAAs (USD 1500 for a 3-month course), local DAAs (USD 200 for a 3-month course) captured only 10% of the Chinese market in 2023, with imported DAAs continuing to dominate the Chinese market [4]. In principle, both local and imported DAAs undergo rigorous evaluation by the National Medical Products Administration (NMPA) to ensure safety and efficacy, and all the approved products should meet the same criteria set by NMPA. However, why did the lower price advantage of the local DAAs not lead to a higher market share in China? And if the small market share of local DAAs was attributable to the potential differences in efficacy and safety between the local and imported DAAs? The public may not have enough confidence in local DAAs. This study addresses this evidence gap, synthesizing existing data to comprehensively evaluate whether local DAAs offer comparable efficacy and safety to imported ones. Due to the lack of direct evidence, we expected that synthesis analysis of the existing data could help to generate comprehensive indirect evidence to address the speculation that local DAAs can ensure substantial efficacy and safety compared to the imported ones. This study targeted all of the five local DAA combinations (DNV-based regimes; RDV-based regimes; emitasvir + sofosbuvir, EMV + SOF; coblopasvir + SOF, CLP + SOF, alfosbuvir + daclatasvir, AOF + DAC) and six imported ones (sofosbuvir + ribavirin, SOF + RBV; elbasvir/grazoprevir, EBR/GZR; ledipasvir/sofosbuvir, LDV/SOF; sofosbuvir/velpatasvir, SOF/VEL; glecaprevir/pibrentasvir, GLE/PIB; sofosbuvir/velpatasvir/voxilaprevir, SOF/VEL/VOX) holding valid registration numbers granted by the NMPA as of January 25, 2024 (Table S1). We systematically reviewed and compared the efficacy and safety of these DAAs. The evidence is expected to be used for promoting DAA treatment of hep C, aiming to achieve the goal of elimination of the public health threats of viral hepatitis in China and globally.

## Methods

The authors followed the Preferred Reporting Items for Systematic Reviews and Meta-analyses reporting guideline to perform the analysis and report the findings [5]. As this study utilized publicly available data without individual patient identifiers, institutional review board approval was deemed not applicable. The PROSPERO registration of this study is CRD42024517554. The authors made four methodological adjustments to the study. The protocol adopted risk ratio as the outcome measure to assess the effect size in the randomized controlled trials (RCTs), we changed it into risk difference (RD). The authors did not perform meta-regression analysis for RCTs due to the limited number of RCTs. The following analyses were not in the protocol, the sensitivity analysis to assess the robustness of the results, and the Duval and Tweedie’s trim-and-fill procedure to correct for publication bias.

### Search strategy

Focused on ‘hepatitis C direct antiviral therapy’, we systematically searched major online databases including China National Knowledge Infrastructure (https://www.cnki.net/), SinoMed (http://www.sinomed.ac.cn/index.jsp), Web of Science, PubMed, Embase, and Cochrane Library. The literature published from the inception of these databases to January 25, 2024 were searched. Detailed searching queries used for each of the above databases were presented in Table S2.

### Inclusion and exclusion criteria

We included RCTs and single-arm trials (SATs) that involved hep C patients as the study subjects and received DAA interventions. The control group of RCTs was pegylated interferon with ribavirin (Peg-IFN+ RBV) or placebo. The included trials should report at least one efficacy or safety primary endpoint. We did not restrict treatment duration and number of patients included, nor the nationality, age, sex, status of the cirrhosis, treatment experience of the participants, and whether they had sexually transmitted infections. This study only included research articles in Chinese and English. Detailed descriptions of the eligibility criteria for the participants, intervention and comparators, study designs were shown in Appendix 1. The number of studies retrieved from respective databases were reported in Table S2.

Considering that some trials might not be published by academic journals, we extracted the clinical trial numbers cited in the Technical Review Reports and NMPA approved pharmaceutical instructions of the targeted DAAs, which are publicly available on the website of the Center for Drug Evaluation (CDE) of NMPA [6]. We compared the extracted clinical trial numbers with the trial numbers cited by academic journals to identify any missing trials. Missing trials were retrieved from CDE’s Drug Clinical Trial Registration and Information Platform [7] and ClinicalTrials.gov [8].

### Data extraction

WZ and RY independently screened the trials that were initially identified through electronic searches, reading titles and abstracts, and removing duplicates. Full-text articles were reviewed and cross-checked to confirm inclusion. Disagreements were resolved through consultation with the corresponding author. For the trials published in multiple sources, the version with the most comprehensive data was selected. Additionally, we also examined the reference lists of the meta-analyses and the pooled analyses, in order to identify any missing studies from the electronic search.

### Outcome measures

The primary outcome measures were 12-week sustained virologic response (SVR12). In addition, relapse, virological breakthrough, adverse events (AEs) and serious adverse events (SAEs) were identified as the secondary outcome measurements. Details of these outcome measures were shown in Appendix 1.

### Quality assessment

WZ and RY used Version 2 of the Cochrane risk-of-bias tool to independently assess the risks of bias of RCTs [9], while SATs were appraised with a checklist for quality appraisal of case series studies developed by the Institute of Health Economics [10]. Disagreements were resolved through consultation with the corresponding author.

### Evidence analyses

We firstly performed pooled analyses of the efficacy and safety evidence for local and imported DAAs respectively. RD and rate of each outcome measure were used to assess the effect size*.* Inter-group outcome differences were evaluated using Cochran’s Q test [11]. Pre-planned subgroup analyses based on specific characteristics of the participants and indicated genotypes of DAAs were performed to lower the heterogeneity. Subgroup effect sizes were compared using Cochran’s Q test to identify any statistically significant differences [12]. Heterogeneity was assessed with the *I*^2^ statistic, where *I*^2^ > 50% indicated high heterogeneity, and a random effect model was applied to account for high heterogeneity in the pooled analyses. Otherwise, a fixed-effect model was used. We performed meta-analysis using the random-effects empirical Bayes model for both RCTs and SATs to mitigate the potential systematic bias stemming from differences in the volume and quality of trials [13].

We also performed meta-regression analyses of SATs to assess the global difference in each outcome measure between local and imported DAAs. Multiple meta-regression analyses were performed based on univariate analyses, adjusting for the potential confounders (mean age, ethnicities, cirrhosis status, treatment experience of patients, and indicated genotype of DAAs) to further evaluate the differences [14].

Sensitivity analyses were conducted to validate the robustness of the results. We firstly removed the studies appraised as with high risk of bias from the pooled analyses, followed by removal of the studies assessed with some concerns of bias. Comparisons were made based on the results of these sensitivity analyses results subsequently. Similar sensitivity analyses were performed for multiple meta-regressions. As Peg-IFN is no longer in use for hep C treatment and has a safety profile very different from DAAs, we performed additional sensitivity analyses of SATs for rates of AEs and SAEs by removing the trials for DAA interventions that contained Peg-IFN. Funnel plots and Egger’s tests assessed publication bias in datasets with ≥ 10 trials [15]. The Duval and Tweedie’s trim and fill procedure was conducted to correct for publication bias, serving as an additional validation to assess whether a small study effect existed in the pooled analyses [16, 17]. The threshold for statistical significance was set at a two-tailed *P* < 0.05 for all analyses. All statistical analyses were performed using Stata/MP 17.0 (StataCorp LLC, Texas, USA) (for data analysis and graphs).

## Results

### Characteristics of the targeted DAAs

As shown in Table S1, 11 DAAs held valid registration numbers granted by NMPA as of January 25, 2024. Of which, five are local and six are imported DAAs. Two out of five local and four out of six imported DAAs are pan-genotypic. Two out of five local and all imported DAAs are indicated for HCV patients with cirrhosis. Two out of five local and no imported DAAs are indicated only for treatment-naïve HCV patients. None of the local and two out of six imported DAAs are indicated for 12–18 years old teenagers. Two out of five local and all imported DAAs are indicated for ≥ 65 years old. No local DAAs are indicated for human immunodeficiency virus (HIV) co-infected hep C patients, and all imported DAAs are indicated for HIV co-infections. Three out of five local and all imported DAAs are indicated for kidney impairment patients.

### Identification of studies

**Fig. 1:**
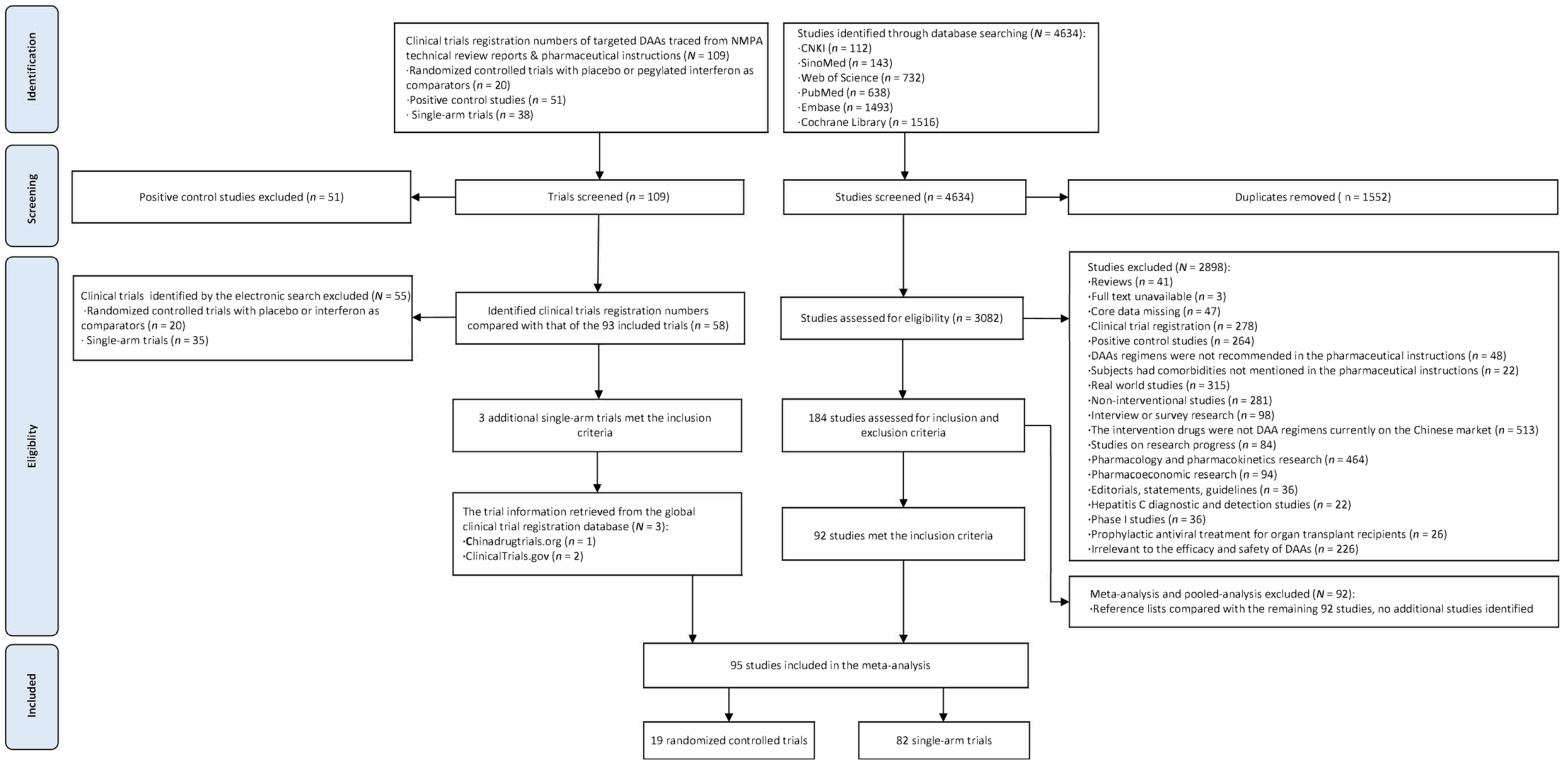
Flowchart of literature search and study selection processes

As shown in Fig. 1, a total of 184 studies met the eligibility criteria. Among which, 92 studies were meta-analyses or pooled analyses, 92 were individual studies. We examined the reference lists of the 92 meta-analyses or pooled analyses, and confirmed no additional citations beyond the 92 individual studies already identified from electronic search. By comparing the clinical trial registration numbers of the 92 individual studies with those cited by CDE Technical Review Reports and NMPA approved pharmaceutical instructions of the targeted DAAs, we identified three additional eligible trials which were outside the original 92 individual studies. A total of 95 studies were finally included in the analysis. Among which, four studies [18–21] reported one RCT and one SAT respectively, and one study [22] reported one RCT and two SATs. A total of 19 RCTs and 82 SATs were included for quantitative synthesis.

### Characteristics of the included trials

As shown in Table S3, among the included 19 RCTs, only two were for local and 17 for imported DAAs. The median number of patients enrolled in the RCTs was 281 (range: 137–424) for local and 301 (range: 68–740) for imported DAAs. The two RCTs of local DAAs were all multi-center trials involved treatment-naïve patients without cirrhosis. Among which, one reported SVR24 only, another had a control group that immediately started DAA treatment after 12 weeks of blinded interventions. SVR12 was not available from RCTs for local DAAs. Among the 17 RCTs for imported DAAs, 14 were multi-center trials, 12 involved cirrhotic patients, and nine involved treatment-experienced patients. Only two RCTs for imported but no RCT for local DAAs involved HIV co-infected patients.

As shown in Table S4, among the 82 SATs, seven were trials for local DAAs, the other 75 were for imported ones. The median enrolled number of patients was 141 (range: 38–371) for local and 100 (range: 9–2400) for imported DAAs. All of the seven SATs for local and 56 out of 75 SATs for imported DAAs were multi-centered trials. Three out of seven SATs for local and 54 out of 75 SATs for imported DAAs involved cirrhotic patients. Three out of seven SATs for local and 54 out of 75 SATs for imported DAAs involved treatment-experienced patients. Ten SATs for imported DAAs involved HIV co-infected patients, while no trials for local DAAs did.

### Quality of the included trials

Details of the risk of bias assessments were presented in Tables S5–6. The only two RCTs for local and seven RCTs for imported DAAs were assessed as with low risk of bias, nine RCTs for imported DAAs were with some concerns of risk, and one RCT for imported DAAs was assessed as with high risk of bias associated with randomization process (Figure S2). Among the 82 SATs, all of the seven trials for local DAAS and 64 for imported DAAs were assessed as with low risk of bias, 11 SATs for imported DAAs were with some concerns of risk, no SAT was assessed as with high risk of bias (Figure S3).

### Efficacy and safety evidence from RCTs: local vs imported DAAs

**Fig. 2:**
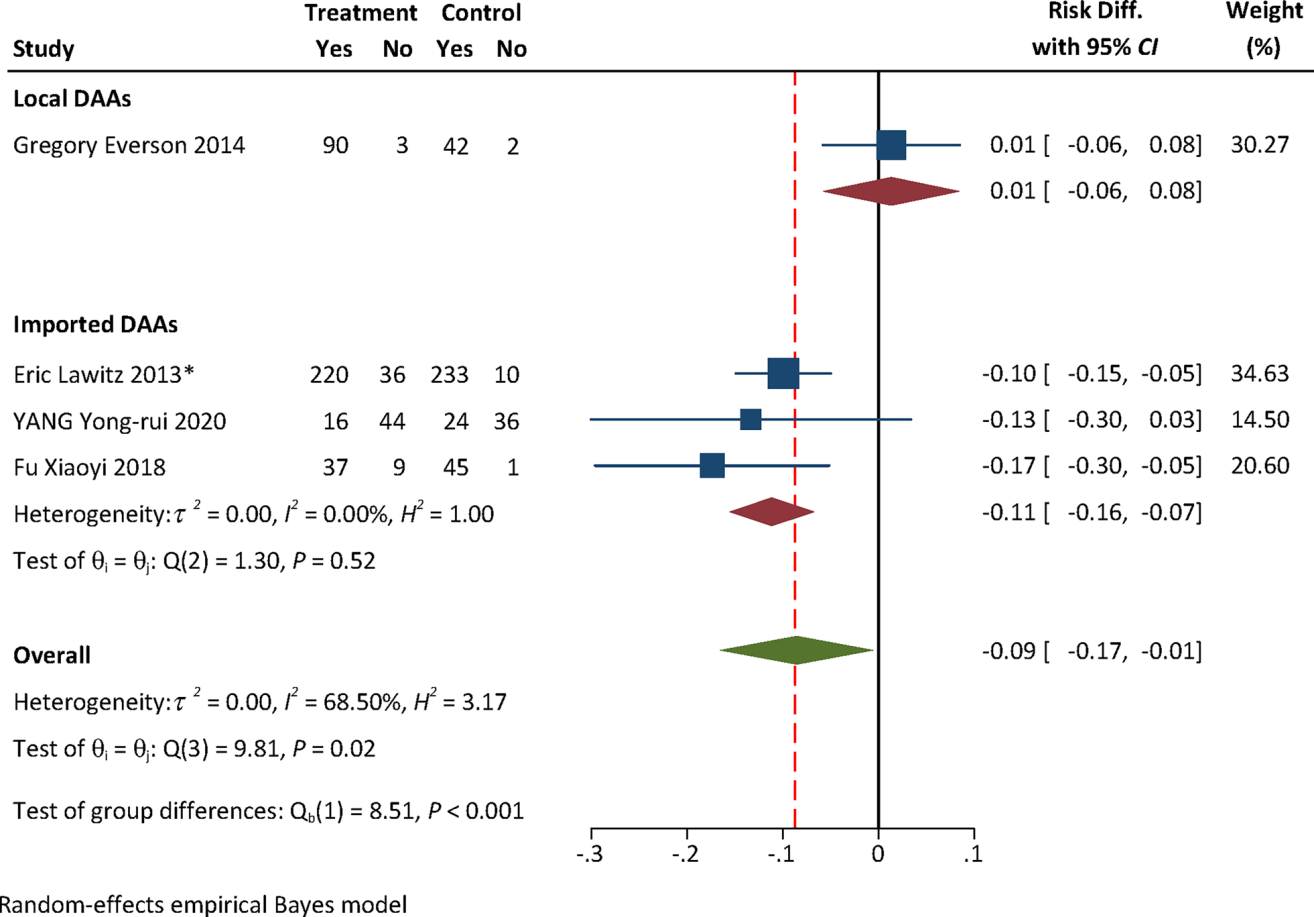
Forest plot for RD of AEs from RCTs with Peg-IFN + RBV as the control. **Notes:** *RD* risk difference, *AEs* adverse events, *RCTs* randomized controlled trials, *Peg-IFN* + *RBV* pegylated interferon and ribavirin, *DAAs* direct-acting antiviral agents; only one RCT for local DAAs was included, heterogeneity analysis was not performed for local DAAs

**Fig. 3:**
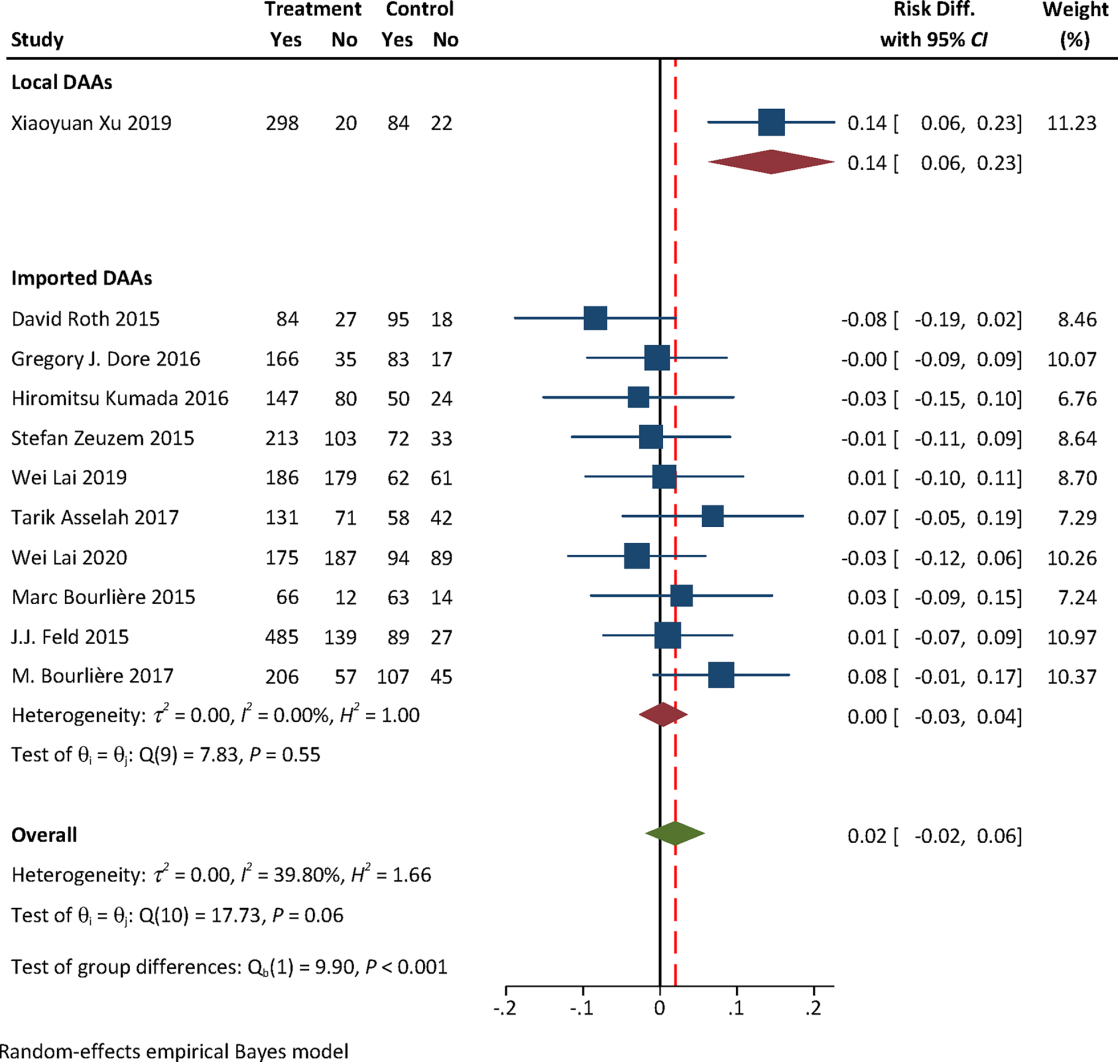
Forest plot for RD of AEs from RCTs with placebo as the control. **Notes:** *RD* risk difference, *AEs* adverse events, *RCTs* randomized controlled trials, *DAAs* direct-acting antiviral agents; only one RCT for local DAAs was included, heterogeneity analysis was not performed for local DAAs

**Fig. 4:**
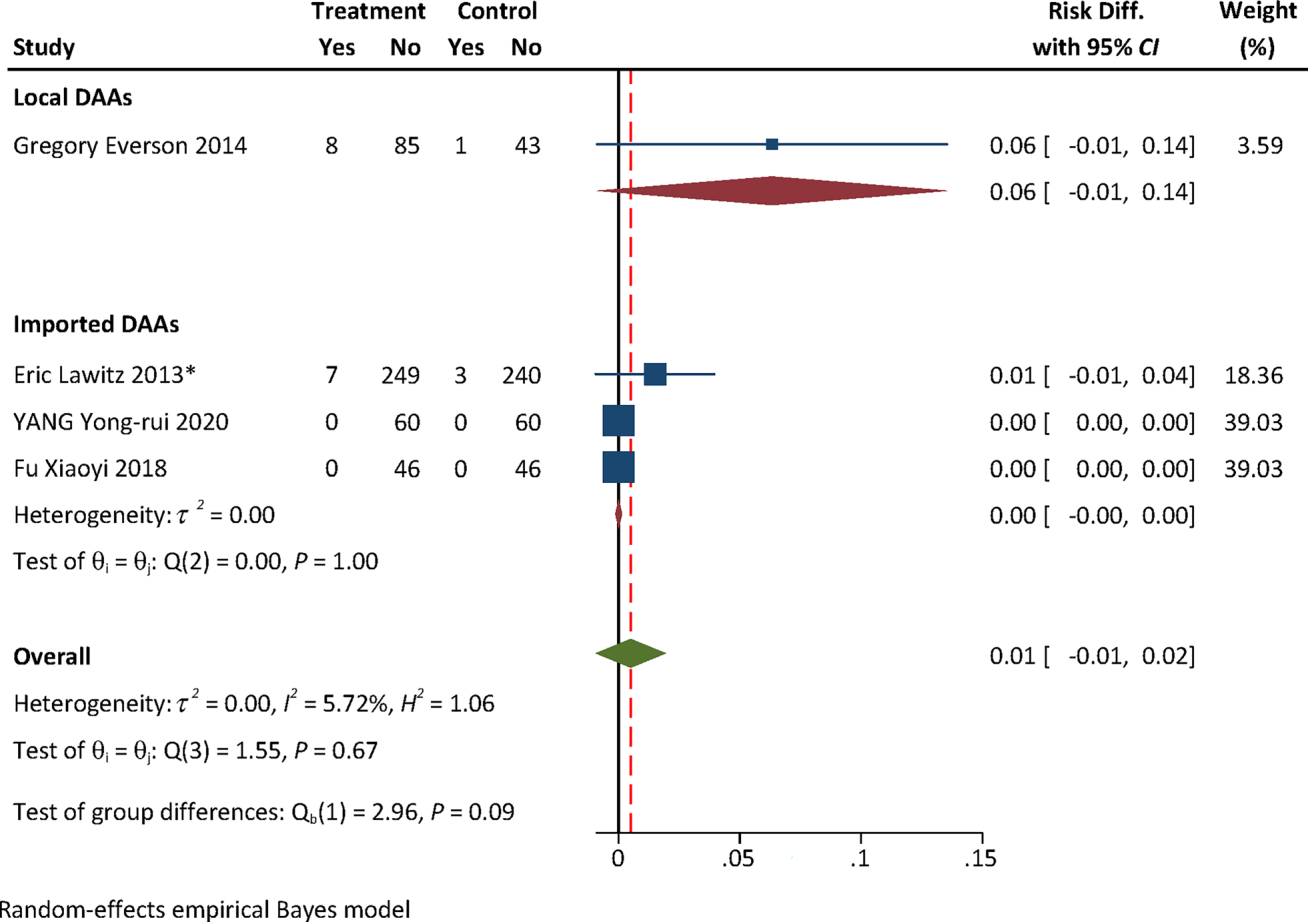
Forest plot for RD of SAEs from RCTs with Peg-IFN + RBV as the control. **Notes:** *RD* Risk difference; *SAEs* serious adverse events; *RCTs* randomized controlled trials; *Peg-IFN* + *RBV* pegylated interferon and ribavirin; *DAAs* direct-acting antiviral agents; only one RCT for local DAAs was included, heterogeneity analysis was not performed for local DAAs; the value of Q test of RCTs for imported DAAs is 0, *I*^2^ and H^2^ cannot be calculated (*I*^2^ = 100%(Q-df)/Q; *I*^2^ = (*H*^2^ − 1)/*H*^2^)

**Fig. 5:**
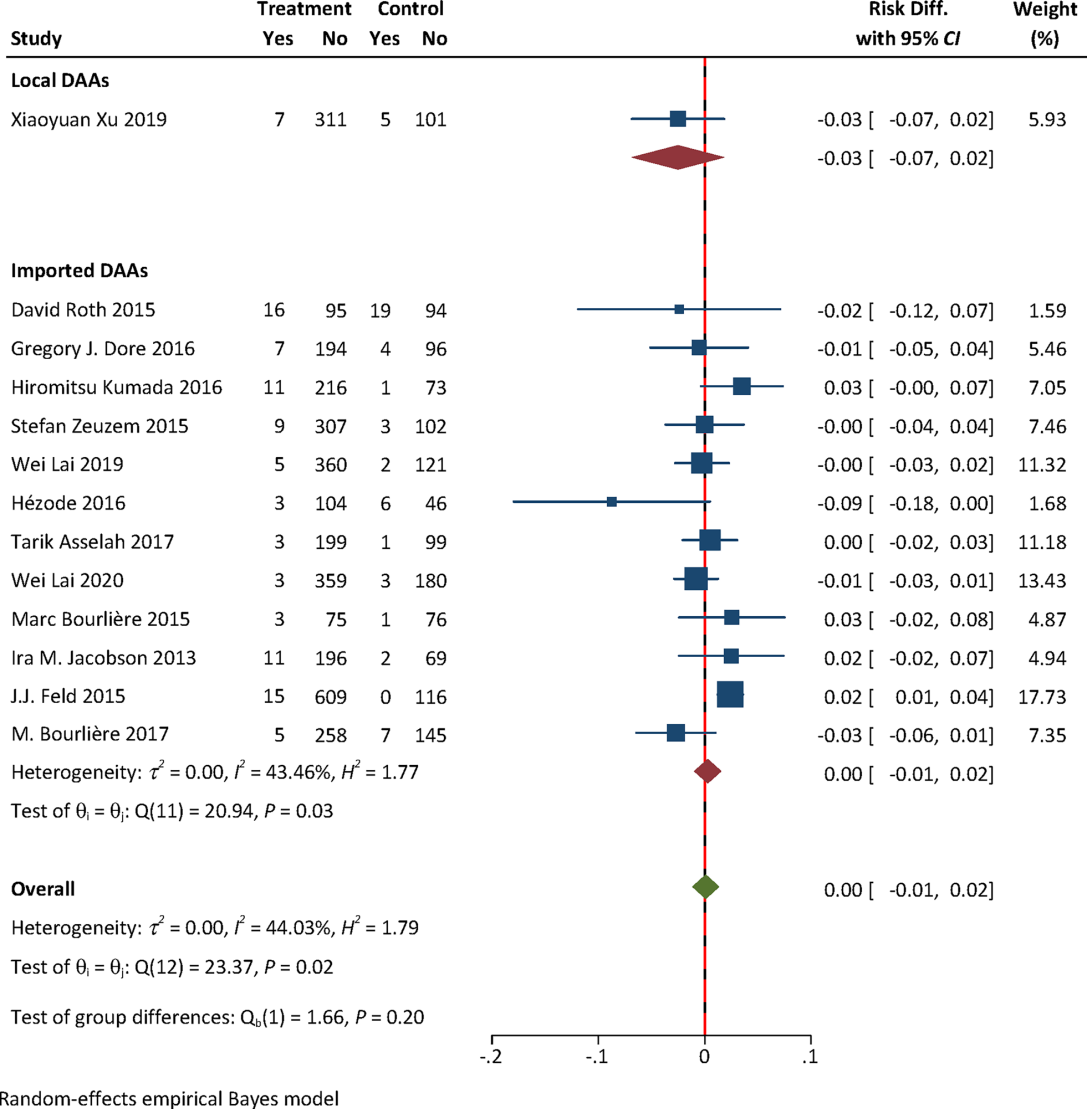
Forest plot for RD of SAEs from RCTs with placebo as the control. **Notes:** *RD* Risk difference; *SAEs* serious adverse events; *RCTs* randomized controlled trials; *DAAs* direct-acting antiviral agents; only one RCT for local DAAs was included, heterogeneity analysis was not performed for local DAAs

No efficacy evidence was available from RCTs for local DAAs. The pooled safety effect size from RCTs for local and imported DAAs were presented in Table 1. The RD for AEs with either Peg-IFN + RBV [0.01, (95% confidence interval (*CI*) − 0.06, 0.08) vs − 0.11 (95% *CI:* − 0.16, − 0.07), *P* = 0.004] or placebo [0.14, (95% *CI:* 0.06, 0.23) vs 0.004, (95% *CI:* − 0.03, 0.04), *P* = 0.002] as the control of RCTs for local DAAs was higher than that for imported ones (Figs. 2, 3). No statistically significant difference in the RD for SAEs was found between local and imported DAAs [0.06, (95% *CI:* − 0.01, 0.14) vs 0.005, (95% *CI:* − 0.001, 0.001), *P* = 0.09, Fig. 4; − 0.03, (95% *CI:* − 0.07, 0.02) vs 0.005, (95% *CI:* − 0.01, 0.02), *P* = 0.20, Fig. 5). The results of subgroup analyses and subsequent comparisons (Tables S7–8) were consistent with those of the synthesis analyses (Table 1). The reported SAEs of local DAAs mainly came from DNV-based regimes, included acute pancreatitis, depression, acute renal failure, anemia and chest pain. These are the typical side effects of Peg-IFN, which is part of the DNV-based regimes. SAEs for imported DAAs included atrial fibrillation, ventricular arrhythmias, atrial fibrillation, myocardial infarction, non-cardiogenic chest pain and hypertensive crisis.

**Table 1:** Summarized findings of synthesis analyses and sensitivity analyses of RCTs for efficacy and safety outcome measures: local vs imported DAAs

Outcome measure	Control	Type of analysis	Origin of DAA	No. of included studies	Experimental group	Control group	Heterogeneity		RD (95% *CI*)	*P*-value
					No. of events/sample size		*I*^*2*^ (%)	*P*		
AEs	PEG-IFN + RBV	Synthesis results	Total	4	363/455	344/393				
			Local	1	90/93	42/44	–	–	0.01 (− 0.06, 0.08)	**0.004**
			Imported	3	273/362	302/349	0.00	0.52	− 0.11 (− 0.16, − 0.07)	
		Sensitivity analyses by excluding high risk of bias	Total	3	326/409	299/347				
			Local	1	90/93	42/44	–	–	0.01 (− 0.06, 0.08)	**0.01**
			Imported	2	236/316	257/303	0.02	0.70	− 0.10 (− 0.15, − 0.05)	
SAEs		Synthesis results	Total	4	15/455	4/392				
			Local	1	8/93	1/43	–	–	0.06 (− 0.01, 0.14)	0.09
			Imported	3^*^	7/362	3/349	–	1.00	0.0005 (− 0.001, 0.001)	
		Sensitivity analyses by excluding high risk of bias	Total	3	15/409	4/347				
			Local	1	8/93	1/44	–	–	0.06 (− 0.01, 0.14)	0.10
			Imported	2^*^	7/316	3/303	–	1.00	0.002 (− 0.01, 0.01)	
AEs	Placebo	Synthesis results	Total	11	2157/3067	857/1249				
			Local	1	298/318	84/106	–	–	0.14 (0.06, 0.23)	**0.002**
			Imported	10	1859/2749	773/1143	0.00	0.55	0.004 (− 0.03, 0.04)	
		Sensitivity analyses by excluding some concerns of risk	Total	7	1328/1936	597/882				
			Local	1	298/318	84/106	–	–	0.14 (0.06, 0.23)	** < 0.01**
			Imported	6	1030/1618	513/776	17.17	0.30	− 0.004 (− 0.05, 0.04)	
SAEs		Synthesis results	Total	13	98/3381	54/1372				
			Local	1	7/318	5/106	–	–	− 0.03 (− 0.07, 0.02)	0.20
			Imported	12	91/3063	49/1266	43.46	0.03	0.005 (− 0.01, 0.02)	
		Sensitivity analyses by excluding some concerns of risk	Total	7	52/1926	43/882				
			Local	1	7/318	5/106	–	–	− 0.03 (− 0.07, 0.02)	0.46
			Imported	6	45/1618	38/776	0.11	0.91	− 0.01 (− 0.02, 0.01)	

*RCTs* randomized controlled trials; *DAAs* direct-acting antiviral agents; *CI* confidence interval; *RD* risk difference; *AEs* adverse events; *SAEs* serious adverse events; *Peg-IFN* + *RBV* pegylated interferon and ribavirin; *P*-value for test of group difference; ^*^ indicates double zero-events existed in RCTs; Heterogeneity is not reported in case that the number of trials ≤ 3; Bold means statistically significant

### Efficacy and safety evidence from SATs: local vs imported DAAs

No statistically significant differences were found from the pooled analyses of SATs for rates of SVR12 [0.97, (95% *CI:* 0.95, 0.99) vs 0.96, (95% *CI:* 0.94, 0.98), *P* = 0.21], relapse [0.02, (95% *CI:* 0.01, 0.04) vs 0.02, (95% *CI:* 0.01, 0.03), *P* = 0.65], virological breakthrough [0.003, (95% *CI:* < 0.001, 0.02) vs 0.0000002, (95% *CI:* < 0.001, 0.0006), *P* = 0.51], and SAEs [0.04, (95% *CI:* 0.03, 0.06) vs 0.03, (95% *CI:* 0.02, 0.03), *P* = 0.12] between local and imported DAAs (Figure S4–6, S8). The rate of AEs of local DAAs was higher than that of imported ones [0.93, (95% *CI:* 0.85, 0.99) vs 0.69, (95% *CI:* 0.64, 0.74), *P* = 0.00002, Figure S7]. The results of synthesis analyses of SATs and subsequent comparisons (Table 2) were consistent with those of RCTs (Table 1).

**Table 2:** Summarized findings of synthesis analyses, meta-regression analyses, sensitivity analyses of SATs for efficacy and safety outcome measures: local vs imported DAAs

Outcome measure	Type of analysis	Origin of DAA	No. of included studies	No. of events/sample size	Heterogeneity		ES (95% *CI*)	*P*-value
					*I*^*2*^ (%)	*P*		
SVR12	Synthesis results	Total	82	11831/12988				
		Local	7	1335/1369	79.01	0.00	0.97 (0.95, 0.99)	0.21
		Imported	75	10496/11619	94.90	0.00	0.96 (0.94, 0.98)	
	Sensitivity analyses by excluding some concerns of risk	Total	71	9766/10828				
		Local	7	1335/1369	79.01	0.00007	0.97 (0.95, 0.99)	0.21
		Imported	64	8431/9459	95.11	0.00	0.96 (0.93, 0.98)	
	• *P*-for-interaction (genotype subgroup difference) = 0.07, *I*^2^ = 94.81% • Univariate meta-regression: global difference between local vs imported DAAs [0.028, (95% *CI:* − 0.061, 0.116), *P* = 0.54; *I*^*2*^ = 45.79%; Adjusted *R*^2^ = 4.65%; *N* = 82] • Multiple meta-regression (adjusted for ethnicity and cirrhosis status of patients): difference between local vs imported DAAs [− 0.037, (95% *CI:* − 0.124, 0.050), *P* = 0.40; *I*^*2*^ = 0.00%; Adjusted *R*^2^ = 55.65%; *N* = 82] • Sensitivity analysis of multiple meta-regression (adjusted for ethnicity and cirrhosis status of patients) by removing trials with ‘some concerns’ [− 0.036, (95% *CI:* − 0.129, 0.058), *P* = 0.45; *I*^2^ = 0.00%; Adjusted *R*^2^ = 51.50%; *N* = 71]							
Relapse	Synthesis results	Total	71	285/9445				
		Local	7	28/1369	69.52	0.00	0.02 (0.01, 0.04)	0.65
		Imported	64	257/8076	79.67	0.00	0.02 (0.01, 0.03)	
	Sensitivity analyses by excluded some concerns of risk	Total	64	223/7668				
		Local	7	28/1369	69.52	0.003	0.02 (0.006, 0.04)	0.69
		Imported	57	195/6299	76.61	0.00	0.02 (0.01, 0.03)	
	• *P*-for-interaction (genotype subgroup difference) = 0.23, *I*^2^ = 78.93% • Univariate meta-regression: global difference between local vs imported DAAs [− 0.011, (95% *CI:* − 0.070, 0.047), *P* = 0.70; *I*^*2*^ = 0.00%; *N* = 71] • Sensitivity analysis of univariate meta-regression by removing trials with ‘some concerns’ [− 0.011, (95% *CI:* − 0.070, 0.049), *P* = 0.73; *I*^2^ = 0.00%; *N* = 64] No statistically significant moderator was found, only performed univariate meta-regression							
Virological break-through	Synthesis results	Total	54	27/7007				
		Local	7	13/1369	79.00	0.00007	0.003 (< 0.001, 0.02)	0.51
		Imported	47	14/5638	0.00	0.98	0.0000002 (< 0.001, 0.0006)	
	Sensitivity analyses by excluding some concerns of risk	Total	53	23/6687				
		Local	7	13/1369	79.00	0.00007	0.002 (< 0.001, 0.02)	0.44
		Imported	46	10/5318	0.00	0.99	< 0.001 (< 0.001, 0.0003)	
	• *P-*for-interaction (genotype subgroup difference) = 0.18, *I*^2^ = 9.72%; • Univariate meta-regression: global difference between local vs imported DAAs [0.007, (95% *CI:* − 0.053, 0.067), *P* = 0.82; *I*^2^ = 0.00%; *N* = 54] • Sensitivity analysis of univariate meta-regression by removing trials with some concerns of risk [0.008, (95% *CI:* − 0.053, 0.068), *P* = 0.80; *I*^2^ = 0.00%; *N* = 53] No statistically significant moderator was found, only performed univariate meta-regression							
AEs	Synthesis results	Total	63	5632/8330				
		Local	7	1150/1369	96.01	0.00	0.93 (0.85, 0.99)	**0.00002**
		Imported	56	4482/6961	95.29	0.00	0.69 (0.64, 0.74)	
	Sensitivity analyses by excluding some concerns of risk	Total	61	5019/7265				
		Local	7	1150/1369	96.01	0.00	0.93 (0.85, 0.99)	**0.00004**
		Imported	54	3869/5896	95.30	0.00	0.70 (0.64, 0.75)	
	Sensitivity analyses by excluding some concerns of risk & DNV-PEG INF-based regimes	Total	57	4712/6955				**0.002**
		Local	3	843/1059	–	–	0.80 (0.76, 0.83)	
		Imported	54	3869/5896	95.30	0.00	0.70 (0.64, 0.75)	
	• *P*-for-interaction (genotype subgroup difference) = 0.81, *I*^2^ = 96.10% • Univariate meta regression: global difference between local vs imported DAAs [0.223, (95% *CI:* 0.082, 0.365), *P* = **0.003**; *I*^2^ = 72.86%; Adjusted *R*^2^ = 17.85%; *N* = 63] • Multiple meta-regression (adjusted for ethnicity of patients): difference between local vs imported DAAs [0.337, (95% *CI:* 0.188, 0.486), *P* < **0.001**; *I*^2^ = 66.74%; Adjusted *R*^2^ = 35.39%; *N* = 63] • Sensitivity analysis of multiple meta-regression (adjusted for treatment experience of patients) by removing trials with ‘some concerns’ [0.235, (95% *CI:* 0.091, 0.379), *P* = **0.002**; *I*^2^ = 69.49%; Adjusted *R*^2^ = 27.00%; *N* = 61] • Sensitivity analysis of multiple meta-regression (adjusted for treatment experience of patients) by removing trials with ‘some concerns’ & DNV-Peg-INF-based regimens [0.255, (95% *CI:* 0.058, 0.452), *P* = **0.012**; *I*^2^ = 68.18%; Adjusted *R*^*2*^ = 25.30%; *N* = 57] Different statistically significant moderator was identified in the sensitivity analyses (treatment experience of patients) compared with that in the original meta-regression (ethnicity of patients)							
SAEs	Synthesis results	Total	71	284/8120				
		Local	7	63/1369	34.75	0.16	0.04 (0.03, 0.06)	0.12
		Imported	64	221/6751	73.74	0.00	0.03 (0.02, 0.03)	
	Sensitivity analyses by excluding some concerns of risk	Total	68	284/7794				
		Local	7	63/1369	34.75	0.16	0.04 (0.03, 0.06)	0.22
		Imported	61	221/6425	72.66	0.00	0.03 (0.02, 0.04)	
	Sensitivity analyses by removing some concerns of risk & DNV-Peg-INF-based regimens	Total	64	274/7484				0.23
		Local	3	53/1059	–	–	0.05 (0.03, 0.08)	
		Imported	61	221/6425	72.66	0.00	0.03 (0.02, 0.04)	
	• *P*-for-interaction (genotype subgroup difference) = **0.005**, *I*^2^ = 73.06% • Univariate meta-regression: global difference between local vs imported DAAs [0.013, (95% *CI:* − 0.046, 0.072), *P* = 0.66; *I*^*2*^ = 0.00%; *N* = 71] • Multiple meta-regression (adjusted for genotype): difference between local vs imported DAAs [0.015, (95% *CI:* − 0.044, 0.074), *P* = 0.61; *I*^2^ = 0.00%; *N* = 71] • Sensitivity analysis of multiple meta-regression (adjusted for genotype) by removing trials with some concerns of risk [0.014, (95% *CI:* − 0.046, 0.073), *P* = 0.64; *I*^2^ = 0.00%; *N* = 68] • Sensitivity analysis of multiple meta-regression (adjusted for genotype) by removing trials with some concerns of risk & DNV-Peg-INF-based regimens [0.015, (95% *CI:* − 0.051, 0.081), *P* = 0.66; *I*^2^ = 0.00%; *N* = 64]							

*SATs* single-arm trials; *DAAs* direct-acting antiviral agents; *CI* confidence interval; *ES* effect size**;** *SVR12* 12-week sustained virologic response, which is undetectable HCV RNA in blood 12 weeks after end of treatment; *AEs* adverse events; *SAEs* serious adverse events; *P*-value for test of group difference; Heterogeneity is not reported in case that the number of trials ≤ 3; Bold means statistically significant

The results of subgroup analyses of SATs and subsequent comparisons (Tables S9, 10) were consistent with those of synthesis analyses (Table 2). SVR12 and virological breakthrough rates in the pan-genotypic subgroup, SAEs rate in the subgroups of Asian patients and genotype-specific DAAs were exceptions. Considering that data extracted from the included SATs provided efficacy and safety evidence for both pan-genotypic and genotype-specific local and imported DAAs, we performed the test of interaction for genotype subgroup difference in each efficacy and safety outcome measure (Figures S9–13). Statistically significant genotype subgroup difference was found only in SAEs rate (*P-*for-interaction = 0.005, *I*^2^ = 73.06%, Figure S13). The genotype subgroup pooled analyses results indicated higher SAEs rate of local genotype-specific DAAs than imported ones [0.04, (95% *CI:* 0.02, 0.05) vs 0.01, (95% *CI:* 0.005, 0.02), *P* = 0.007], but no statistically significant difference between local pan-genotypic DAAs and imported ones [0.05, (95% *CI:* 0.04, 0.07) vs 0.04, (95% *CI:* 0.02, 0.05), *P* = 0.42]. SAEs reported by SATs for local DAAs were consistent with those reported by RCTs, both were mainly related to DNV-based treatment regimens. The SAEs reported by SATs for imported DAAs were consistent with those reported by RCTs.

The univariate meta-regressions of SATs identified ethnicities and cirrhosis status of patients (*P* < 0.05) as the predictors for SVR12 rate, ethnicities of patients (*P* < 0.1) as the predictors for AEs rate. No statistically significant predictors were identified for rates of relapse, virological breakthrough and SAEs. The global differences between local and imported DAAs in all efficacy and safety outcome measures drawn from the univariate meta-regressions without adjustment of potential moderator were not statistically significant, except in AEs rate. In the univariate meta-regressions, the AEs rate of local DAAs was 22.3% higher than that of imported DAAs [0.223, (95% *CI:* 0.082, 0.365), *P* = 0.003, Table 2 and Table S11].

By adjusting for ethnicities and cirrhosis status of patients in multiple meta-regression, we did not find statistically significant difference in SVR12 rate [− 0.037, (95% *CI:* − 0.124, 0.050), *P* = 0.40, Table 2 and Table S12]. By controlling for ethnicities of patients in multiple meta-regression, we found that the AEs rate of local was 33.7% higher than that of imported DAAs [0.337, (95% *CI:* 0.188, 0.486), *P* < 0.001, Table 2 and Table S12]. By adjusting for indicated genotype of DAAs in the multiple meta-regression, we found no statistically significant difference in SAEs rate [0.015, (95% *CI:* − 0.044, 0.074), *P* = 0.61, Table 2 and Table S12]. The results of the multiple meta-regressions adjusted for potential moderators were consistent with those of the univariate meta-regressions and synthesis analyses and subsequent comparisons.

### Sensitivity analyses and publication bias

We firstly removed the only one RCT assessed as with high risk of bias from pooled analyses of RCTs with Peg-IFN + RBV as the control for imported DAAs to perform the sensitivity analyses for the safety outcome measures. The results indicated that RD for AEs of local DAAs was statistically significantly higher than that of imported ones [0.01, (95% *CI:* − 0.06, 0.08) vs − 0.10, (95% *CI:* − 0.15, − 0.05),* P* = 0.01, Table 1]. No statistically significant difference in RD of SAEs was found [0.06, (95% *CI:* − 0.01, 0.14) vs 0.002, (95% *CI:* − 0.01, 0.01), *P* = 0.10, Table 1]. These were consistent with the results of synthesis analyses and subsequent comparisons (Table 1). For RCTs with placebo as the control for imported DAAs, we conducted the sensitivity analyses by removing six RCTs assessed as with some concerns of risk from pooled analysis of safety outcome measures. The results of sensitivity analyses demonstrated that RD for AEs of local DAAs was statistically significantly higher than that of imported ones [0.14, (95% *CI:* 0.06, 0.23) vs − 0.004, (95% *CI:* − 0.05, 0.04), *P* < 0.01]. No statistically significant difference in RD of SAEs was found [− 0.03, (95% *CI:* − 0.07, 0.02) vs − 0.01, (95% *CI:* − 0.02, 0.01),* P* = 0.46, Table 1]. These were consistent with the results of synthesis analyses and subsequent comparisons (Table 1).

We removed 11 SATs of imported DAAs assessed as with some concerns of risk from the pooled analyses for efficacy and safety outcome measures (Table 2). The results of the sensitivity analyses and the original synthesis analyses were consistent with each other. The rate of AEs of local was higher than that of imported DAAs [0.93, (95% *CI:* 0.85, 0.99) vs 0.70, (95% *CI:* 0.64, 0.75), *P* = 0.00004], and no statistically significant difference in the rate of SAEs [0.04, (95% *CI:* 0.03, 0.06) vs 0.03, (95% *CI:* 0.02, 0.04),* P* = 0.22, Table 2]. By removing the four trials associated with DNV-based regimes which contain Peg-IFN, the results were still consistent, with the *P* value for differences between the rate of AEs of local and imported DAAs changed to 0.002 [0.80, (95% *CI:* 0.76, 0.83) vs 0.70, (95% *CI:* 0.64, 0.75), *P* = 0.002]. Similar sensitivity analyses were performed for meta-regressions. By removing the 11 SATs assessed as with some concerns of risk from multiple meta-regression adjusted for ethnicities and cirrhosis status of patients, we did not find statistically significant difference in SVR12 rate [− 0.036, (95% *CI:* − 0.129, 0.058),* P* = 0.45, Table 2]. By adjusting for treatment experience of patients, we found 23.5% higher AEs rate of local than imported DAAs [0.235, (95% *CI:* 0.091, 0.379), *P* = 0.002]. By adjusting for indicated genotype of DAA, we did not observe statistically significant difference in SAEs rate [0.014, (95% *CI:* − 0.046, 0.073),* P* = 0.64, Table 2]. The results of sensitivity analyses of the meta-regressions were consistent with those of the original meta-regressions, synthesis analyses and subsequent comparisons based on both RCTs and SATs analyses (Table 1, 2 and Tables S13, 14).

Results showed in Figures S14–29 suggested that only the funnel plots and Egger tests for SVR12 rate from SATs for imported DAAs detected potential publication bias (*P* < 0.001). After adjustment with imputed studies, SVR12 rate of imported DAAs was slightly attenuated to 0.91 (95% *CI:* 0.89, 0.94).

## Discussion

The clinical evidence of local DAAs is significantly fewer than that of imported ones, in terms of both the number of studies and the diversity of targeted patients. Trials for imported DAAs enrolled demographically diverse participants in different age groups, cirrhosis stages, treatment histories, and HIV co-infection statuses. In contrast, trials for local DAAs predominantly enrolled treatment-naïve and non-cirrhosis HCV infections. Only two trials for local DAAs enrolled elderly patients, and no trials for local DAAs enrolled adolescent patients and patients co-infected with HIV. This has important implications for the local DAA developers, and serves as an important flag for them to continue strengthening the evidence in the real-world with expanded patient population, and to improve the quality of the evidence presented to regulators. A more forward-looking approach should be adopted in China, as most imported DAAs are mostly oral regimes and pan-genotypic, which is the standard care recommended by the WHO. It is also critical to strengthen the post-marketing pharmacovigilance of DAA treatment to collect comprehensive safety information, thereby to better inform treatment decisions.

The results of synthesis analyses and adjusted multiple meta-regressions were consistent with each other. The analyses results based on RCTs and SATs were also consistent with each other, and came with the same findings of no statistically significant differences in efficacy and SAEs between local and imported DAAs, but higher AEs of the former compared with that of the latter. In particular, the SAEs rate of local genotype-specific DAAs was significantly higher than that of imported ones. The results of sensitivity analyses also aligned with the original findings, confirming that the general conclusions remained consistent.

Findings of higher AEs rate of local than imported DAAs, and higher SAEs rate of local genotype-specific DAAs than imported ones were consistent with the safety descriptions in CDE Technical Review Reports and NMPA approved pharmaceutical instructions of relevant DAAs. The drug interaction study of CLP and SOF showed that the AEs rate for CLP was significantly higher than that of SOF, but comparable to that of CLP in combination with SOF. The regulatory files for local genotype-specific DNV-based regimes [23, 24] indicated that most SAEs were associated with Peg-IFN/RBV/RTV components. Additionally, five cases of acute pancreatitis occurred during treatment, possibly related to any components of the DNV-based regimes, which should be given particular attention, and corresponding risk control measures should be developed. The investigators of two pan-genotypic DAAs (CLP and AOF) stated that the observed SAEs were not associated with the study medicines, and could recover gradually with or without medication. Overall, no special safety issues were concerned for imported DAAs.

Based on the existing efficacy evidence, all DAAs can achieve over 90% of SVR12, which is a standard measure for the success of hep C treatment. Therefore, the safety profile of these treatment regimens becomes particularly important. According to the regulatory files, although most mild to moderate AEs associated with local DAAs are generally tolerable and reversible. However, both mild and severe AEs can cause short-term discomfort in patients’ daily lives, which may affect the continuity of treatment and patient adherence. Several regional and national surveys about Chinese patients’ knowledge, awareness and perceptions of HCV treatment found that psychosocial factors represent significant barriers to accessing HCV treatment, with safety concerns and potential side effects being among the top concerns, followed closely by the economic burden of treatment [25, 26]. Both treatment-naïve and treatment-experienced patients expressed a fear of side effects and showed strong preferences for treatments with minimal adverse reactions [27]. The NS3/4A protease inhibitors (PIs) included in the local DNV-based regimes and imported EBR/GZR, GLE/PIB, SOF/VEL/VOX are metabolized by liver. This metabolism may increase the likelihood of hepatocellular damage, leading to a higher frequency of AEs. Therefore, these medicines are contraindicated in patients with compromised liver function to avert the risk of decompensation, and are not recommended without close clinical or laboratory supervision [28]. Many Chinese HCV patients are diagnosed late due to poor awareness, often when their disease has already progressed to cirrhosis [29]. Local regimens such as RDV and EMV could not be used by this large group of Chinese patients, as they are indicated only for non-cirrhotic patients. Evidence supports that favorable overall safety and tolerability profile of local pan-genotypic DAAs, like CLP and AOF, showed a generally favorable overall safety and tolerability profile for patients with compensated cirrhosis and mild renal impairment. The known and potential risks of CLP can be effectively managed through a risk control plan [30, 31]. There is substantial evidence supporting the use of imported DAAs like LDV/SOF, SOF/VEL and EBR/GZR for patients with concomitant liver damage (compensated cirrhosis, decompensated cirrhosis, and liver transplantation) or kidney damage (chronic kidney disease, hemodialysis, and renal failure) [32, 33]. Additionally, some imported DAA regimens, such as the LDV/SOF and GLE/PIB, offer an 8-week oral treatment regimen, which is a very important course for shortening the treatment duration.

China’s HCV epidemiological landscape exhibits distinct characteristics. There are quite a proportion of HCV patients in China infected through blood transfusions before implementation of the Blood Donation Law in 1998. This historical transmission route has created a substantial patient cohort with prolonged infection durations exceeding two decades [34]. These elderly now enter advanced age with increasing comorbidities. Most of them are under polypharmacy, which may potentially interact with DAAs. The Guideline for Prevention and Treatment of Hepatitis C suggests evaluating patient’s comorbidities and polypharmacy before starting antiviral therapy, and loosely monitor for the potential drug-drug interactions (DDIs). DAAs with a lower likelihood of DDIs should be adopted for patients on multiple medications. CYP450 is a crucial hepatic drug-metabolizing enzyme involved in drug metabolism, most drugs are metabolized by CYP450, which increases the risk of DDIs. NS3/4A PIs are typically genotype-specific DAAs, while NS5B and NS5A inhibitors are pan-genotypic DAAs. A vast majority of NS3/4A PIs are metabolized by CYP450, which may affect the activity of CYP450. As a result, NS3/4A PIs are more likely to have DDIs. In contrast, NS5B and NS5A inhibitors demonstrate negligible CYP450 involvement [35]. Local NS5A inhibitors, including CLP and AOF, are therefore associated with fewer DDIs and potentially lower AEs incidence. Imported pan-genotypic DAAs generally have good tolerability even among patients with multiple treatment experiences.

Local DAA developers should put in place post-marketing risk management strategies, strengthen the pharmacovigilance system, and regularly update the safety reports. Furthermore, they should also continue the trials involving expanded patients in terms of age, treatment experience, cirrhosis status, co-infections and kidney impairment, etc. The real-world studies could help to collect information about DDIs and long-term safety, providing a more comprehensive picture of DAA treatment in practice.

In addition, genotype distribution patterns further complicate therapeutic strategies. The prevalent HCV genotypes in China are 1b and 2a [28], followed by genotype 3, particularly subtype 3b, which is common in certain subpopulations and at-risk groups. Genotype 3 is considered more difficult to cure, as many oral DAAs are less effective against it [36, 37]. The diversity of genotypes in China necessitates a potent pan-genotypic treatment regimen. Local DNV, RDV, and EMV are indicated only for genotype 1. SOF is currently under intellectual property right protection, thus local DAAs cannot be marketed alongside generic SOF to form full pan-genotypic combinations. Considering that the patent holder already had the market authorization of DAC lapsed in China, and announced no enforcement of its patent right over DAC, the newly approved AOF in 2023 comes as another core DAA like SOF for pan-genotypic combination. It can be used in combination with DAC to form a full local pan-genotypic combination with the potential of better efficacy and safety. This combination could play a pivotal role in the simplified treatment of hep C, as recommended by international guidelines [38].

This study has several limitations. First, as local DAAs are only marketed in China and were marketed later than imported ones, the number of trials available for this study was relatively small and the available evidence is sparse. The imbalance in the evidence base between local and imported DAAs might weaken the robustness of the analyses. Using the random-effects empirical Bayes model to conduct the synthesis analyses helped to mitigate the potential systematic bias stemming from differences in the volume and quality of trials. As more evidence for local DAAs is available, it is necessary to further validate the findings of this analysis through future studies. Second, although all included trials for local DAAs were assessed as with ‘low risk of bias’, some trials for imported DAAs were assessed as with ‘high risk of bias’ or ‘some concerns’ of risks. The results of sensitivity analyses yielded consistent results. Future attention to real-world evidence is critical to further validate our main findings. Third, as there are no head-to-head studies that directly compared the efficacy and safety of local and imported DAAs, this study only included RCTs with placebo or Peg-IFN + RBV as the control. Based on an initial literature search, the authors found that the number of head-to-head studies of DAAs is small and only conducted between imported DAAs. Due to the lack of direct evidence about the efficacy and safety differences between local and imported DAAs, we conducted this meta-analysis based on the existing evidence, and generated indirect evidence. The indirect nature of the evidence limits the conclusiveness of the findings. Clinical and policy decision-making should be adaptive and evolve as new evidence is generated. Additionally, comparisons were based on studies with different populations and settings with the potential of confounding and bias. Some synthesis analyses exhibited high heterogeneity. Although random effects models were applied to address this, and the sensitivity analyses valid the results of the synthesis analyses to some extent, the findings must be interpreted with caution. It is necessary to conduct further synthesis analysis for each subgroup to validate the findings of this study when more evidence for local DAAs is available. Lastly, the studies with positive results are more likely to be reported or published and were inevitably more likely to be included in this study. The trim and fill procedure might help to reduce the publication bias. Despite these limitations, to the best of our knowledge, this is the first study which systematically reviewed, analyzed and compared the efficacy and safety evidence generated by not only RCTs but also SATs for the local and imported DAAs in China.

## Conclusions

Current evidence does not demonstrate statistically significant difference in efficacy and SAEs between local and imported DAAs. Considering that the current standard of care is simplified treatment with pan-genotypic DAAs, local pan-genotypic DAAs have the potential to contribute to increasing the treatment rate of hep C. It is critical for local DAAs to generate more evidence with expanded patient population. It is also critical to strengthen the post-marketing pharmacovigilance of DAA treatment to collect comprehensive safety information to better inform treatment. Head-to-head studies directly comparing the efficacy and safety of local and imported DAAs are also needed. Thus, to boost the confidence of clinicians in local DAAs, which also has the global significance of the access potential for low-and-middle income countries that are still not on track to eliminate hep C. Clinical and policy decision-making should be adaptive and evolve as new evidence is generated.

## Supplementary Information

Below is the link to the electronic supplementary material.

Supplementary file1 (DOCX 5160 KB)

## Data Availability

The study was based on open data, no primary data to be shared. The data supporting the findings of our study are available within this article and the supplementary. The statistical plan and code for analyses are available on request from the corresponding author without any access criteria.

## Abbreviations

HCV: Hepatitis C virus
Hep C: Hepatitis C
DAAs: Direct-acting antiviral agents
RDV: Ravidasvir
SOF: Sofosbuvir
DNV: Danoprevir
RBV: Ribavirin
NMPA: National Medical Products Administration
RCTs: Randomized controlled trials
SATs: Single-arm trials
CDE: Center for Drug Evaluation
EMA: European Medicines Agency
FDA: Food and Drug Administration
Peg-IFN + RBV: Pegylated interferon with ribavirin
SVR12: 12-week sustained virologic response
AEs: Adverse event
SAEs: Serious adverse events
RD: Risk difference
HIV: Human immunodeficiency virus
*CI*: Confidence interval
ES: Effect size
CLP: Coblopasvir
EBR/GZR: Elbasvir/grazoprevir
GLE/PIB: Glecaprevir/pibrentasvir
SOF/VEL: Sofosbuvir/velpatasvir/voxilaprevir
LDV/SOF: Ledipasvir/sofosbuvir
SOF/VEL: Sofosbuvir/velpatasvir
AOF: Alfosbuvir
DDIs: Drug–drug interactions
PIs: Protease inhibitors
