# Supplementary material for: Are there differences in efficacy and safety between local and imported direct-acting antiviral agents for hepatitis C in China?
Source: Infect Dis Poverty. 2025 Jul 25;14:75. doi: 10.1186/s40249-025-01344-2 (PMC12291330; doi:10.1186/s40249-025-01344-2)
Supplement: Supplementary file 1 — Supplementary file1 (DOCX 5160 KB) [file 40249_2025_1344_MOESM1_ESM.docx]

Supplementary material

**Directory**

[**Appendix 1:** A detailed description of the eligibility criteria 1](#_Toc202121363)

[**Table S1:** Characteristics of local and imported DAAs 3](#_Toc202121364)

[**Figure S1:** Timeline of market authorization and public funding coverage of targeted DAAs 6](#_Toc202121365)

[**Table S2:** Search queries and results 7](#_Toc202121366)

[**Table S3:** Characteristics of the included RCTs 10](#_Toc202121367)

[**Table S4:** Characteristics of the included SATs 12](#_Toc202121368)

[**Table S5:** Risk of bias appraisal of individual RCTs 18](#_Toc202121369)

[**Table S6:** Risk of bias appraisal of individual SATs 19](#_Toc202121370)

[**Figure S2:** Summary of overall risk of bias of individual RCTs 24](#_Toc202121371)

[**Figure S3:** Summary of overall risk of bias of individual SATs 25](#_Toc202121372)

**[Table S7:](#_Toc202121373)** [Subgroup analyses of RCTs with Peg-IFN+RBV as the control for safety outcome measures: locally developed vs imported DAAs 27](#_Toc202121373)

[**Table S8:** Subgroup analyses of RCTs with placebo as the control for safety outcome measures: locally developed vs imported DAAs 28](#_Toc202121374)

[**Table S9:** Subgroup analyses of SATs for efficacy outcome measures: locally developed vs imported DAAs 29](#_Toc202121375)

[**Table S10:** Subgroup analyses of SATs for safety outcome measures: locally developed vs imported DAAs 32](#_Toc202121376)

[**Figure S4:** Forest plot for SVR12 rate from SATs: locally developed vs imported DAAs 34](#_Toc202121377)

[**Figure S5:** Forest plot for relapse rate from SATs: locally developed vs imported DAAs 35](#_Toc202121378)

[**Figure S6:** Forest plot for virological breakthrough rate from SATs: locally developed vs imported DAAs 36](#_Toc202121379)

[**Figure S7:** Forest plot for AE rate from SATs: locally developed vs imported DAAs 37](#_Toc202121380)

[**Figure S8:** Forest plot for SAE rate from SATs: locally developed vs imported DAAs 38](#_Toc202121381)

[**Figure S9:** Forest plot for SVR12 rate from SATs: pan-genotypic vs genotype-specific DAAs 39](#_Toc202121382)

[**Figure S10:** Forest plot for relapse rate from SATs: pan-genotypic vs genotype-specific DAAs 40](#_Toc202121383)

[**Figure S11:** Forest plot for virological breakthrough rate from SATs: pan-genotypic vs genotype-specific DAAs 41](#_Toc202121384)

[**Figure S12:** Forest plot for AE rate from SATs: pan-genotypic vs genotype-specific DAAs 42](#_Toc202121385)

[**Figure S13:** Forest plot for SAE rate from SATs: pan-genotypic vs genotype-specific DAAs 43](#_Toc202121386)

[**Table S11:** Univariate meta-regression of SATs for efficacy and safety outcome measures 44](#_Toc202121387)

[**Table S12:** Multiple meta-regression of SATs for efficacy outcome measures 49](#_Toc202121388)

[**Figure S14:** Publication bias for RD of AEs from RCTs 50](#_Toc202121389)

[**Figure S15:** Publication bias for RD of AEs from RCTs with placebo as the control 51](#_Toc202121390)

[**Figure S16:** Publication bias for RD of AEs from RCTs with placebo as the control of imported DAAs 52](#_Toc202121391)

[**Figure S17:** Publication bias for RD of SAEs from RCTs 53](#_Toc202121392)

[**Figure S18:** Publication bias for RD of SAEs from RCTs with placebo as the control 54](#_Toc202121393)

[**Figure S19:** Publication bias for RD of SAEs from RCTs with placebo as the control of imported DAAs 55](#_Toc202121394)

[**Figure S20:** Publication bias for SVR12 rate from SATs 56](#_Toc202121395)

[**Figure S21:** Publication bias for SVR12 rate from SATs of imported DAAs 57](#_Toc202121396)

[**Figure S22:** Publication bias for relapse rate from SATs 58](#_Toc202121397)

[**Figure S23:** Publication bias for relapse rate from SATs of imported DAAs 59](#_Toc202121398)

[**Figure S24:** Publication bias for virological breakthrough rate from SATs 60](#_Toc202121399)

[**Figure S25:** Publication bias for virological breakthrough rate from SATs of imported DAAs 61](#_Toc202121400)

[**Figure S26:** Publication bias for AE rate from SATs 62](#_Toc202121401)

[**Figure S27:** Publication bias for AE rate from SATs of imported DAAs 63](#_Toc202121402)

[**Figure S28:** Publication bias for SAE rate from SATs 64](#_Toc202121403)

[**Figure S29:** Publication bias for SAE rate from SATs of imported DAAs 65](#_Toc202121404)

[**Table S13:** Sensitivity analyses of univariate meta-regressions of SATs (removed ‘some concerns’) 66](#_Toc202121405)

[**Table S14:** Sensitivity analyses of multiple meta-regressions of SATs (removed ‘some concerns’) 71](#_Toc202121406)

[**Table S15:** The Preferred Reporting Items for Systematic Reviews and Meta-Analyses for Protocols 72](#_Toc202121407)

[**List of included studies** 76](#_Toc202121408)

Appendix 1: A detailed description of the eligibility criteria

**Inclusion criteria**

**Participants:** patients diagnosed with hepatitis C virus (HCV) infection. We did not limit the nationality, age, sex, treatment experience of the targeted patients. We did not exclude whether the participants had sexually transmitted infections such as Human immunodeficiency virus (HIV), or whether they had different stages of liver disease progression. This is because that different DAAs target different HCV patients, including treatment naïve vs treatment experienced, children and adults vs adults only, and different cirrhosis status, co-infections. If we exclude any of the above specific group of patients, the comparison between locally and imported DAAs would not be comprehensive. Sub-group analyses will help to compare locally developed and imported DAAs in different groups of HCV patients.

**Intervention and Comparators:** direct-acting antiviral agents (DAAs) holding a valid registration number of the National Medical Products Administration (NMPA) of China as of January 25, 2024, and forming the treatment regimens that are in line with the NMPA approved pharmaceutical instructions **(Table S1)**. The control group of randomized controlled trials (RCTs) was pegylated interferon with or without ribavirin (Peg-IFN+RBV) or placebo. We did not have any restrictions on treatment duration, or number of patients included.

**Outcome measures:** the efficacy outcome measures were HCV sustained virologic response, which is undetectable HCV RNA in the blood 12 weeks after the end of treatment (SVR12); HCV relapse, which is undetectable HCV RNA during treatment and/or at end of treatment, but subsequent detectable HCV RNA following treatment cessation; and HCV virological breakthrough, which is re-emergence or significant increase of HCV RNA that had been reduced to undetectable level during treatment , indicates resistant to treatment or failed treatment. The safety outcome measures were any adverse events (AEs) and serious adverse events (SAEs).

**Study designs:** the Center for Drug Evaluation (CDE) of NMPA accepts evidence generated by SATs for the review of market authorization applications of DAAs, which aligns with the principles of the European Medicines Agency (EMA) and the Food and Drug Administration (FDA). So, we included interventional RCTs and single-arm trials (SATs). In addition, the primary endpoints of RCTs and SATs should include at least one the outcome measures of SVR12; relapse; virological breakthrough, AEs and SAEs.

**Exclusion criteria**

1. Reviews
2. Full text unavailable
3. Core data missing
4. Clinical trial registration
5. Positive control studies
6. DAAs regimens were not recommended in the pharmaceutical instructions
7. Subjects had comorbidities not mentioned in the pharmaceutical instructions
8. Real world studies
9. Non-interventional studies
10. Interview or survey research
11. The intervention drugs were not DAA regimens currently on the Chinese market
12. Studies on research progress
13. Pharmacology and pharmacokinetics research
14. Pharmacoeconomic research
15. Editorials, statements, guidelines
16. Hepatitis C diagnostic and detection studies
17. Phase I studies
18. Prophylactic antiviral treatment for organ transplant recipients
19. Irrelevant to the efficacy and safety of DAAs

Table S1: Characteristics of local and imported DAAs

| DAA | Dosage Form & Strength | Mechanism of action | Origin of DAA | Indication | Indicated genotype | Inclusion of patients | | | | | | Time of market authorization | Time of public funding coverage |
| --- | --- | --- | --- | --- | --- | --- | --- | --- | --- | --- | --- | --- | --- |
|  |  |  |  |  |  | Cirrhosis status | Treatment experience | 12-18 years old teenagers | ≥ 65 years old elderly | HIV co-infected | Renal impairment |  |  |
| Sofosbuvir, SOF | Tab: 400mg | NS5B polymerase inhibitor | Imported | HCV infection | Pan-genotypic (combined with ribavirin) | Mixed | Mixed | Yes | Yes | Yes | Yes | 2017/9/20 | / |
| Elbasvir/Grazoprevir, EBR/GZR | FDC: 50mg/100mg | NS3/4A protease inhibitor/NS5A inhibitor | Imported | HCV infection in adults | GT1,4 | Mixed | Mixed | No | Yes | Yes | Yes | 2018/4/28 | 2020/1/1 |
| Sofosbuvir/Velpatasvir, SOF/VEL | FDC: 400mg/100mg | NS5B polymerase inhibitor/NS5A inhibitor | Imported | HCV infection in adults | Pan-genotypic | Mixed | Mixed | No | Yes | Yes | Yes | 2018/5/23 | 2020/1/1 |
| Ledipasvir/Sofosbuvir, LDV/SOF | Tab: 90mg/400mg | NS5A inhibitor/NS5B polymerase inhibitor | Imported | HCV infection | GT1, 3, 4, 5, 6 | Mixed | Mixed | Yes | Yes | Yes | Yes | 2018/11/21 | 2020/1/1 |
| Glecaprevir/Pibrentasvir, GLE/PIB | FDC: 100mg/40mg | NS3/4A protease inhibitor/NS5A inhibitor | Imported | HCV infection in adults | Pan-genotypic | Mixed | Mixed | No | Yes | Yes | Yes | 2019/5/15 | / |
| Sofosbuvir/Velpatasvir/Voxilaprevir, SOF/VEL/VOX | FDC: 400mg/100mg/100mg | NS5B polymerase inhibitor/NS5A inhibitor/NS3/4A protease inhibitor | Imported | DAA treatment experienced HCV infection | Pan-genotypic | Mixed | Experienced | No | Yes | Yes | Yes | 2019/12/18 | 2022/1/1 |
| Danoprevir, DNV | Tab: 100mg | NS3/4A protease inhibitor | Local | HCV infection in treatment-naive, non-cirrhotic adults | GT1b (combined with ritonavir, pegylated interferon and ribavirin) | None | Naïve | No | No | / | / | 2018/6/7 | 2022/1/1 |
| Coblopasvir, CLP | Cap: 60mg | NS5A inhibitor | Local | HCV infection in adults | Pan-genotypic: GT1,2,3,6 (combined with SOF) | Mixed | Mixed | No | No | / | Yes | 2020/2/11 | 2021/3/1 |
| Ravidasvir, RDV | Tab: 200mg | NS5A inhibitor | Local | Treatment-naive non-cirrhotic HCV infection in  adults | GT1b (combined with DNV, ritonavir and ribavirin) * | None | Naïve | No | No | / | / | 2020/7/29 | 2022/1/1 |
| Emitasvir, EMV | Cap: 100mg | NS5A inhibitor | Local | Non-cirrhotic chronic HCV infection in adults | GT1 (combined with SOF) | None | Mixed | No | Yes | / | Yes | 2020/12/21 | 2022/1/1 |
| Alfosbuvir, AOF | Tab: 100mg | NS5B polymerase inhibitor | Local | HCV infection in adults | Pan-genotypic: GT1, 2, 3, 6 (combined with daclatasvir) | Mixed | Mixed | No | Yes | / | Yes | 2023/5/17 | 2024/1/1 |

Notes: *DAA* direct-acting antiviral agent; *HCV* hepatitis C virus; *NMPA* National Medical Products Administration; *Tab* tablet; *FDC* fixed-dose combination; *Cap* capsule; *GT* genotype; Mixed means research subjects include both non-cirrhosis and cirrhosis, or both treatment naïve and treatment experienced patients; EBR/GZR is not covered by public funding after January 1, 2024; *RDV is registered in China for genotype 1b in combination with ritonavir-intensified danoprevir and ribavirin. However, it is registered as a pan-genotypic in combination with sofosbuvir in Malaysia and Egypt. This study only included the trials with the interventional DAA treatment regimens that are in line with the NMPA Technical Review Reports and the NMPA approved pharmaceutical instructions.

Figure S1: Timeline of market authorization and public funding coverage of targeted DAAs


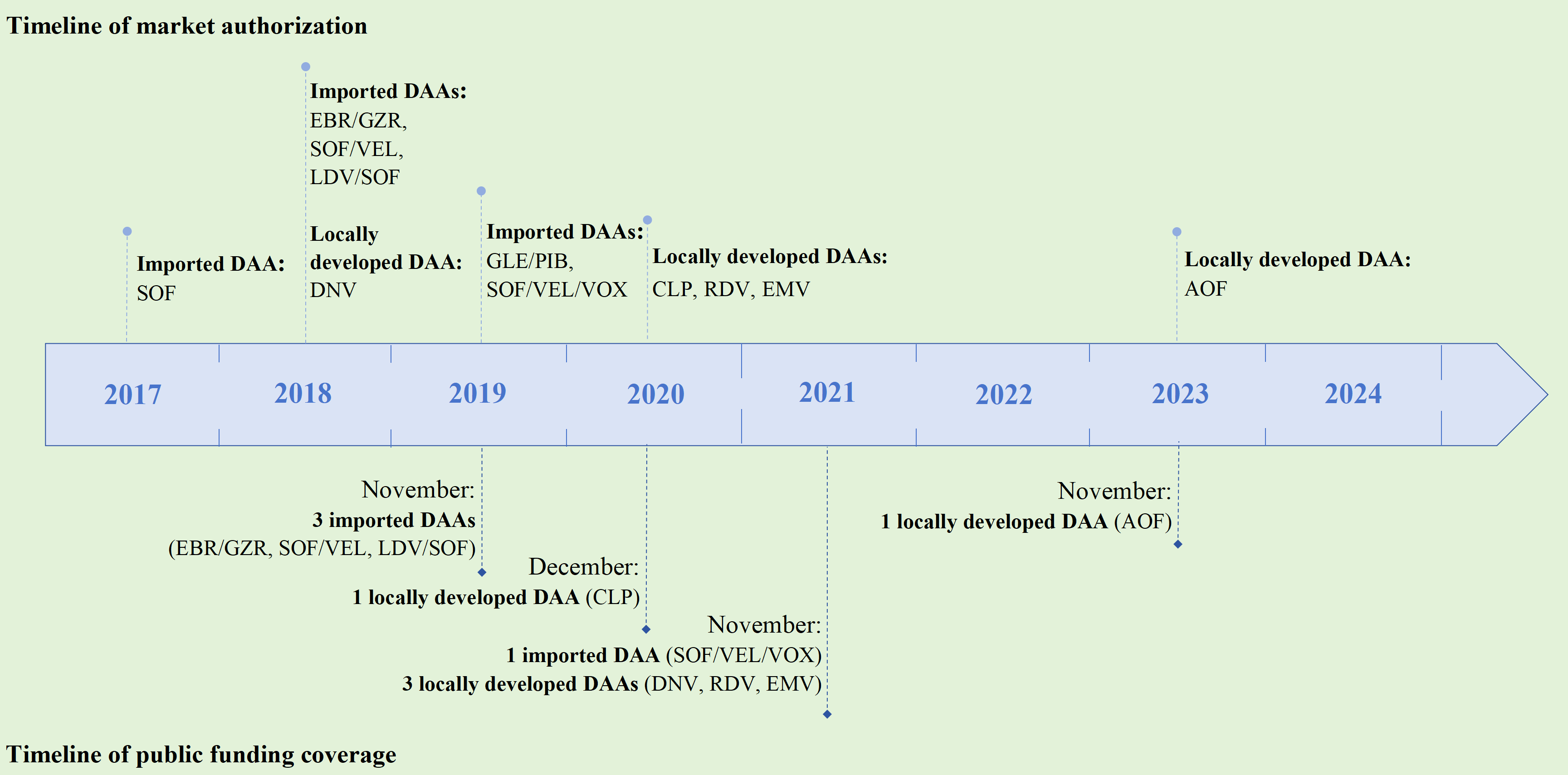


Notes: *DAA* direct-acting antiviral agent; *NMPA* National Medical Products Administration; *SOF* sofosbuvir; *EBR/GZR* elbasvir/grazoprevir; *SOF/VEL* sofosbuvir/velpatasvir; *LDV/SOF* ledipasvir/sofosbuvir; *DNV* danoprevir; *SOF/VEL/VOX* sofosbuvir/velpatasvir/voxilaprevir; *GLE/PIB* glecaprevir/pibrentasvir; *CLP* coblopasvir hydrochloride; *RDV* ravidasvir hydrochloride; *EMV* emitasvir phosphate; *AOF* alfosbuvir; EBR/GZR is not covered by public funding after January 1, 2024.

Table S2: Search queries and results

Focused on ‘hepatitis C direct antiviral therapy’, we systematically searched major online databases including China National Knowledge Infrastructure, SinoMed, Web of Science, PubMed, Embase, and Cochrane Library with publications dated from the inceptions to January 25, 2024. The search strategy included all possible combinations of the following terms: (1) Medical Subject Headings of ‘Hepatitis C’, ‘HCV’, ‘Parenterally-Transmitted Non-A, Non-B Hepatitis’, ‘Parenterally Transmitted Non A, Non B Hepatitis’, ‘PT-NANBH’, ‘Hepatitis, Viral, Non-A, Non-B, Parenterally-Transmitted’, ‘DAA’, ‘Direct Acting Antiviral’, ‘Directly Acting Antiviral’, ‘Direct-Acting Antiviral’; (2) free-text terms of ‘Sofosbuvir’, ‘Elbasvir’, ‘Grazoprevir’, ‘Velpatasvir’, ‘Danoprevir’, ‘Ledipasvir’, ‘Glecaprevir’, ‘Pibrentasvir’, ‘Voxilaprevir’, ‘Coblopasvir’, ‘Ravidasvir’, ‘Emitasvir’, ‘Alfosbuvir’. We selected ‘Clinical Trials’ in the Filters section of the database.

| Database | Query | *N* = 4634 |
| --- | --- | --- |
| CNKI | TKA = ('HCV' + '丙肝'+'丙型病毒性肝炎'+ '丙型肝炎') AND TKA = ('直接抗病毒药物' +'DAA' +'索磷布韦' + '艾尔巴韦'+'格拉瑞韦' + '维帕他韦' + '达诺瑞韦' + '来迪派韦' + '格卡瑞韦'+'派仑他韦' + '索磷维伏' + '可洛派韦' + '拉维达韦' + '依米他韦'+'奥磷布韦') AND SU% = ('临床试验') | 112 |
| SinoMed | (("HCV"[常用字段:智能] OR "丙肝"[常用字段:智能] OR "丙型病毒性肝炎"[常用字段:智能] OR "丙型肝炎"[常用字段:智能]) AND ("直接抗病毒药"[常用字段:智能] OR "DAA"[常用字段:智能] OR "索磷布韦"[常用字段:智能] OR "艾尔巴韦"[常用字段:智能] OR "格拉瑞韦"[常用字段:智能] OR "维帕他韦"[常用字段:智能] OR "达诺瑞韦"[常用字段:智能] OR "来迪派韦"[常用字段:智能] OR "格卡瑞韦"[常用字段:智能] OR "派仑他韦"[常用字段:智能] OR "索磷维伏"[常用字段:智能] OR "可洛派韦"[常用字段:智能] OR "拉维达韦"[常用字段:智能] OR "依米他韦"[常用字段:智能] OR "奥磷布韦"[常用字段:智能])) AND ("临床试验"[文献类型]) | 143 |
| Web of Science | [TS = (("Hepatitis C" OR HCV OR "Parenterally-Transmitted Non-A, Non-B Hepatitis" OR "Parenterally Transmitted Non A, Non B Hepatitis" OR "PT-NANBH" OR "Hepatitis, Viral, Non-A, Non-B, Parenterally-Transmitted") AND (DAA OR "Direct Acting Antiviral" OR "Directly acting antiviral" OR "Direct-Acting Antiviral" OR Sofosbuvir OR Elbasvir OR Grazoprevir OR Velpatasvir OR Danoprevir OR Ledipasvir OR Glecaprevir OR Pibrentasvir OR Voxilaprevir OR Coblopasvir OR Ravidasvir OR Emitasvir OR Alfosbuvir) ) AND DT = (Clinical Trial)](https://webofscience.clarivate.cn/wos/alldb/summary/53bea7ac-e0e8-4e72-afd7-2fc605d195b1-c84eb95c/relevance/1) | 732 |
| PubMed | ("Hepatitis C"[Mesh Terms] OR HCV[Title/Abstract] OR "Parenterally-Transmitted Non-A, Non-B Hepatitis"[Title/Abstract] OR "Parenterally Transmitted Non A, Non B Hepatitis"[Title/Abstract] OR "PT-NANBH"[Title/Abstract] OR "Hepatitis, Viral, Non-A, Non-B, Parenterally-Transmitted"[Title/Abstract]) AND (DAA[Title/Abstract] OR "Direct Acting Antiviral"[Title/Abstract] OR "Directly Acting Antiviral"[Title/Abstract] OR "Direct-Acting Antiviral"[Title/Abstract] OR Sofosbuvir[Title/Abstract] OR Elbasvir[Title/Abstract] OR Grazoprevir[Title/Abstract] OR Velpatasvir[Title/Abstract] OR Danoprevir[Title/Abstract] OR Ledipasvir[Title/Abstract] OR Glecaprevir[Title/Abstract] OR Pibrentasvir[Title/Abstract] OR Voxilaprevir[Title/Abstract] OR Coblopasvir[Title/Abstract] OR Ravidasvir[Title/Abstract] OR Emitasvir[Title/Abstract] OR Alfosbuvir[Title/Abstract]) AND (clinical trial[Filter]) | 638 |
| Embase | (((('hepatitis c' OR 'hcv' OR 'Parenterally-Transmitted Non-A, Non-B Hepatitis' OR 'Parenterally Transmitted Non A, Non B Hepatitis' OR 'PT-NANBH' OR 'Hepatitis, Viral, Non-A, Non-B, Parenterally-Transmitted'):ab,ti)) AND ((daa OR 'direct acting antiviral' OR 'directly acting antiviral' OR 'direct-acting antiviral' OR sofosbuvir OR elbasvir OR grazoprevir OR velpatasvir OR danoprevir OR ledipasvir OR glecaprevir OR pibrentasvir OR voxilaprevir OR coblopasvir OR ravidasvir OR emitasvir OR alfosbuvir):ab,ti)) AND ('clinical trial'/de) | 1493 |
| Cochrane Library | (("Hepatitis C" OR "HCV" OR "Parenterally-Transmitted Non-A, Non-B Hepatitis" OR "Parenterally Transmitted Non A, Non B Hepatitis" OR "PT-NANBH" OR "Hepatitis, Viral, Non-A, Non-B, Parenterally-Transmitted") AND ("DAA" OR "Direct Acting Antiviral" OR "Directly Acting Antiviral" OR "Direct-Acting Antiviral" OR "Sofosbuvir" OR "Elbasvir" OR "Grazoprevir" OR "Velpatasvir" OR "Danoprevir" OR "Ledipasvir" OR "Glecaprevir" OR "Pibrentasvir" OR "Voxilaprevir" OR "Coblopasvir" OR "Ravidasvir" OR "Emitasvir" OR "Alfosbuvir")):ti,ab,kw in Trials | 1516 |

**Table S3:** Characteristics of the included RCTs

| Included study | Clinical trial number | Setting | Blinding | Sample size | Mean age | Ethnicity | Cirrhosis status | Treatment experience | Treatment regimen | Control |
| --- | --- | --- | --- | --- | --- | --- | --- | --- | --- | --- |
| Locally developed DAAs | | | | | | | | | | |
| Gregory Everson 2014 [[1](#_ENREF_1)] | NCT01220947 | Multi-center | No | 137 | 52 | White: 113, Black: 14, Other: 10 | Non-cirrhosis: 137 | Naïve: 137 | DNV-based | Peg-IFN +RBV |
| Xiaoyuan Xu 2019 [[2](#_ENREF_2)] | NCT03362814 | Multi-center | Yes | 424 | 47 | Asian: 424 | Non-cirrhosis: 424 | Naïve: 424 | RDV-based | Placebo |
| Imported DAAs | | | | | | | | | | |
| David Roth 2015 [[3](#_ENREF_3)] | NCT02092350 | Multi-center | Yes | 224 | 56 | White: 103, Black: 103, Asian: 14, Other: 4 | Non-cirrhosis: 210, Cirrhosis: 14 | Naïve: 179, Experienced: 45 | EBR/GZR | Placebo |
| Gregory J. Dore 2016 [[4](#_ENREF_4)] | NCT02105688 | Multi-center | Yes | 301 | 47 | White: 241, Black: 38, Asian: 16, Other: 6 | Non-cirrhosis: 239, Cirrhosis: 62 | Naïve: 301 | EBR/GZR | Placebo |
| Hiromitsu Kumada 2016 [[5](#_ENREF_5)] | NCT02203149 | Multi-center | Yes | 301 | 61 | Asian: 301 | Non-cirrhosis: 301 | Naïve: 198, Experienced: 103 | EBR/GZR | Placebo |
| Stefan Zeuzem 2015 [[6](#_ENREF_6)] | NCT02105467 | Multi-center | Yes | 421 | 53 | White: 264, Black: 77, Asian: 67, Other: 13 | Non-cirrhosis: 329, Compensated cirrhosis:92 | Naïve: 421 | EBR/GZR | Placebo |
| Wei Lai 2019 [[7](#_ENREF_7)] | NCT02251990 | Multi-center | Yes | 488 | 48 | White: 135, Asian: 350, Other: 1, Missing: 2 | Non-cirrhosis: 396, Compensated cirrhosis:90, Missing: 2 | Naïve: 488 | EBR/GZR | Placebo |
| Hézode 2016 [[8](#_ENREF_8)] | NA | Multi-center | Yes | 159 | 44 | Black: 29, Other: 130 | Non-cirrhosis: 121, Cirrhosis: 38 | Mixed | EBR/GZR | Placebo |
| Tarik Asselah 2017 [[9](#_ENREF_9)] | NCT02640482 | Multi-center | Yes | 302 | 57 | White: 181, Black: 14, Asian: 101, Other: 6 | Non-cirrhosis: 302 | Naïve: 212, Experienced: 90 | EBR/GZR | Placebo |
| Wei Lai 2020 [[10](#_ENREF_10)] | NCT03222583 | Multi-center | Yes | 545 | 49 | Asian: 545 | Non-cirrhosis: 545 | Naïve: 436, Experienced: 109 | EBR/GZR | Placebo |
| Marc Bourlière 2015 [[11](#_ENREF_11)] | NCT01965535 | Multi-center | Yes | 155 | 56 | White: 151, Black: 4 | Compensated cirrhosis:155 | Experienced: 155 | LDV/SOF | Placebo |
| Eric Lawitz 2013* [[12](#_ENREF_12)] | NCT01497366 | Multi-center | No | 499 | 48 | White: 435, Black: 17, Asian: 29, Other: 18 | Non-cirrhosis: 399, Compensated cirrhosis:100 | Naïve: 499 | SOF+RBV | Peg-IFN +RBV |
| Ira M. Jacobson 2013 [[13](#_ENREF_13)] | NCT01542788 | Multi-center | Yes | 278 | 52 | White: 254, Black: 13, Asian: 8, Other: 3 | Non-cirrhosis: 234, Compensated cirrhosis: 44 | Naïve: 259, Experienced: 19 | SOF+RBV | Placebo |
| YANG Yong-rui 2020 [[14](#_ENREF_14)] | NA | Single-center | NA | 120 | 37 | Asian: 120 | Mixed | NA | SOF+RBV | Peg-IFN +RBV |
| Eric Lawitz 2013 [[15](#_ENREF_15)] | NCT01188772 | Multi-center | Yes | 73 | 50 | White: 58, Black: 12, Other: 3 | Non-cirrhosis: 73 | Naïve: 73 | SOF+RBV | Peg-IFN +RBV |
| Fu Xiaoyi 2018 [[16](#_ENREF_16)] | NA | Single-center | No | 92 | 39 | Asian: 70 | Compensated cirrhosis: 78 | Naïve: 78 | SOF/VEL | Peg-IFN +RBV |
| Huang Maohui 2022 [[17](#_ENREF_17)] | NA | Single-center | NA | 68 | 49 | Asian: 68 | NA | NA | SOF/VEL | Peg-IFN +RBV |
| J.J. Feld 2015 [[18](#_ENREF_18)] | NCT02201940 | Multi-center | Yes | 740 | 54 | White: 583, Black: 63, Asian: 73, Other: 18, Missing: 3 | Non-cirrhosis: 598, Compensated cirrhosis:142 | Naïve: 506, Experienced: 234 | SOF/VEL | Placebo |
| M. Bourlière 2017 [[19](#_ENREF_19)] | NCT02607735 | Multi-center | Yes | 415 | 59 | White: 335, Black: 60, Asian: 14, Other: 6 | Non-cirrhosis: 243, Compensated cirrhosis:172 | Experienced: 415 | SOF/VEL/ VOX | Placebo |

Notes: * means the same author and publication year, but different trail. *RCTs* randomized controlled trials; *DAAs* direct-acting antiviral agents; *DNV* danoprevir; *Peg-IFN+RBV* pegylated interferon and ribavirin; *RDV* ravidasvir; *EBR/GZR* elbasvir/grazoprevir; *LDV/SOF* ledipasvir/sofosbuvir; *SOF* sofosbuvir; *SOF/VEL* sofosbuvir/velpatasvir; *SOF/VEL/VOX* sofosbuvir/velpatasvir/voxilaprevir.

**Table S4:** Characteristics of the included SATs

| Included study | Clinical trial number | Setting | Sample size | Mean age | Ethnicity | Cirrhosis status | Treatment experience | Treatment regimen |
| --- | --- | --- | --- | --- | --- | --- | --- | --- |
| Locally developed DAAs | | | | | | | | |
| Rui Hua 2023 [[20](#_ENREF_20)] | NCT04070235 | Multi-center | 326 | 50 | Asian: 326 | Non-cirrhosis: 285, Cirrhosis:41 | Naïve: 303, Experienced: 23 | AOF+DAC |
| Yanhang Gao 2020 [[21](#_ENREF_21)] | NCT03995485 | Multi-center | 371 | 41 | Asian: 295, Other: 76 | Non-cirrhosis: 332, Compensated cirrhosis: 39 | Naïve: 332, Experienced: 29 | CLP+SOF |
| Sujun Zheng 2017 [[22](#_ENREF_22)] | NCT03020004 | Multi-center | 70 | 41 | Asian: 70 | Non-cirrhosis: 70 | Naïve: 70 | DNV-based |
| Lai Wei 2019 [[23](#_ENREF_23)] | [NCT03020082](http://clinicaltrials.gov/show/NCT03020082) | Multi-center | 141 | 42 | Asian: 141 | Non-cirrhosis: 141 | Naïve: 141 | DNV-based |
| Jia-Horng Kao 2016 [[24](#_ENREF_24)] | NCT01749150 | Multi-center | 61 | 51 | Asian: 61 | Non-cirrhosis: 34, Cirrhosis: 27 | Naïve: 61 | DNV-based |
| Huiying Rao 2020 [[25](#_ENREF_25)] | NCT03487107 | Multi-center | 362 | 47 | Asian: 362 | Non-cirrhosis: 362 | Naïve: 289, Experienced: 73 | EMV+SOF |
| Jia-Horng Kao 2018 [[26](#_ENREF_26)] | NCT03020095 | Multi-center | 38 | 60 | Asian: 38 | Non-cirrhosis: 38 | Naïve: 38 | RDV-based |
| Imported DAAs | | | | | | | | |
| Hiromitsu Kumada 2016 [[5](#_ENREF_5)] | NCT02203149 | Multi-center | 35 | 65 | Asian: 35 | Compensated cirrhosis: 35 | Naïve: 20, Experienced: 15 | EBR/GZR |
| Anne Boerekamps 2019 [[27](#_ENREF_27)] | NCT02600325 | Multi-center | 80 | 47 | White: 72, Not Reported: 8 | NA | NA | EBR/GZR |
| Tarik Asselah 2017 [[9](#_ENREF_9)] | NCT02636595 | Multi-center | 121 | 53 | White: 84, Black: 10, Asian: 24, Other: 3 | Non-cirrhosis: 121 | Naïve: 82, Experienced: 39 | EBR/GZR |
| Ahmad AlEid 2022 [[28](#_ENREF_28)] | NCT03578640 | Single-center | 30 | 44 | NA | NA | Naïve: 30 | EBR/GZR |
| Armand Abergel 2020 [[29](#_ENREF_29)] | ANSM number: 160789A-41 | Multi-center | 112 | 54 | NA | Non-cirrhosis: 112 | Naïve: 112 | EBR/GZR |
| Jürgen K Rockstroh 2015 [[30](#_ENREF_30)] | NCT02105662 | Multi-center | 218 | 49 | White: 167, Black: 38, Asian: 16, Other: 7 | Non-cirrhosis: 183, Compensated cirrhosis: 35 | NA | EBR/GZR |
| Maria Buti 2016 [[31](#_ENREF_31)] | NCT02105454 | Multi-center | 79 | 54 | White: 77, Black: 2 | Non-cirrhosis: 45, Compensated cirrhosis: 34 | Experienced: 79 | EBR/GZR+RBV |
| Tarik Asselah 2017* [[9](#_ENREF_9)] | NCT02243293 | Multi-center | 203 | 52 | White: 155, Black: 21, Asian: 23, Other: 4 | Non-cirrhosis: 203 | Naïve: 176, Experienced: 27 | EBR/GZR |
| Xavier Forns 2017 [[32](#_ENREF_32)] | NCT02642432 | Multi-center | 146 | 60 | White: 120, Black: 15, Other: 11 | Compensated cirrhosis: 146 | Naïve: 110, Experienced: 36 | GLE/PIB |
| Nancy Reau 2018 [[33](#_ENREF_33)] | [NCT02692703](https://clinicaltrials.gov/ct2/show/NCT02692703) | Multi-center | 100 | 59 | White: 78, Black: 8, Asian: 10, Other: 4 | Non-cirrhosis: 100 | Naïve: 66, Experienced: 34 | GLE/PIB |
| Masanori Atsukawa 2019 [[34](#_ENREF_34)] | UMIN000032073 | Multi-center | 141 | 68 | Asian: 141 | Non-cirrhosis: 100, Cirrhosis:41 | Naïve: 132, Experienced: 9 | GLE/PIB |
| Tarik Asselah 2019 [[35](#_ENREF_35)] | NCT02966795 | Multi-center | 84 | 59 | White: 25, Black: 1, Asian: 57, Multi-ethnicity: 1 | Non-cirrhosis: 75, Compensated cirrhosis: 9 | Naïve: 76, Experienced: 8 | GLE/PIB |
| Eric Lawitz 2019 [[36](#_ENREF_36)] | NCT0306936 | Multi-center | 101 | 58 | White: 74, Black: 14, Asian: 13 | Non-cirrhosis: 86, Compensated cirrhosis: 14, Missing: 1 | Naïve: 81, Experienced: 20 | GLE/PIB |
| Kovesdy C.P 2017 [[37](#_ENREF_37)] | NCT02651194 | NA | 104 | NA | NA | Non-cirrhosis: 84, Compensated cirrhosis: 20 | Naïve: 60, Experienced: 44 | GLE/PIB |
| Robert S. Brown 2020 [[38](#_ENREF_38)] | NCT03089944 | Multi-center | 343 | 58 | White: 285, Black: 28 | Compensated cirrhosis: 343 | Naïve: 343 | GLE/PIB |
| Lai Wei 2020 [[10](#_ENREF_10)] | NCT03235349 | Multi-center | 160 | 58 | Asian: 160 | Compensated cirrhosis: 160 | Naïve: 110, Experienced: 50 | GLE/PIB |
| Mario Peribañez-Gonzalez 2020 [[39](#_ENREF_39)] | NCT03219216 | Multi-center | 100 | 56 | White: 63, Black: 26, Asian: 2, Multi-ethnicity: 9 | Non-cirrhosis: 74, Compensated cirrhosis: 26 | Naïve: 100 | GLE/PIB |
| Jia-Horng Kao 2016* [[40](#_ENREF_40)] | NCT02021643 | Multi-center | 87 | 53 | Asian: 87 | Non-cirrhosis: 74, Compensated cirrhosis: 13 | Naïve: 43, Experienced: 44 | SOF+RBV |
| S. H. Ahn 2016 [[41](#_ENREF_41)] | NCT02021643 | Multi-center | 129 | 55 | Asian: 129 | Non-cirrhosis: 116, Compensated cirrhosis: 13 | Naïve: 105, Experienced: 24 | SOF+RBV |
| KHALID MAHMUD KHAN 2017 [[42](#_ENREF_42)] | NA | Multi-center | 320 | NA | NA | NA | NA | SOF+RBV |
| Rui Huang 2019 [[43](#_ENREF_43)] | NCT02021643 | Multi-center | 126 | 41 | NA | Non-cirrhosis: 102, Compensated cirrhosis: 24 | Naïve: 89, Experienced: 37 | SOF+RBV |
| Fumimasa Tomooka 2017 [[44](#_ENREF_44)] | NA | NA | 38 | 65 | NA | NA | NA | SOF+RBV |
| Michael Charlton 2015 [[45](#_ENREF_45)] | NCT01687270 | Multi-center | 40 | 50 | White: 34, Black: 3, Asian: 2, Other: 1 | Non-cirrhosis: 24, Cirrhosis: 16 | Naïve: 5, Experienced: 35 | SOF+RBV |
| Michael P. Curry 2015 [[46](#_ENREF_46)] | NCT01559844 | Multi-center | 61 | 59 | White: 55, Black: 6 | Cirrhosis: 61 | Naïve: 15, Experienced: 46 | SOF+RBV |
| Stanislas Pol 2015 [[47](#_ENREF_47)] | NA | Multi-center | 80 | 55 | White: 66, Black: 11, Asian: 3 | NA | Experienced: 80 | SOF+Peg-IFN+RBV |
| [Sherief Abd-Elsalam 2016](https://www.eurekaselect.com/article/javascript:void(0)) [[48](#_ENREF_48)] | NA | NA | 2400 | 54 | NA | Cirrhosis: 2400 | Naïve: 2318, Experienced: 82 | SOF+RBV |
| Masao Omata 2014 [[49](#_ENREF_49)] | NCT01910636 | Multi-center | 153 | 57 | Asian: 153 | Non-cirrhosis: 136, Compensated cirrhosis: 17 | Naïve: 90, Experienced: 63 | SOF+RBV |
| Eric Lawitz 2013 [[15](#_ENREF_15)] | NCT01188772 | Multi-center | 25 | 47 | White: 20, Black: 4, Other: 1 | Non-cirrhosis: 25 | Naïve: 25 | SOF+Peg-IFN+RBV |
| Eric Lawitz 2013* [[12](#_ENREF_12)] | NCT01641640 | Multi-center | 327 | 52 | White: 257, Black: 54, Asian: 7, Other: 9 | Non-cirrhosis: 273, Compensated cirrhosis: 54 | Naïve: 327 | SOF+Peg-IFN+RBV |
| Maribel Rodriguez-Torres 2015 [[50](#_ENREF_50)] | NCT01565889 | Single-center | 23 | 47 | White: 15, Black: 8 | Non-cirrhosis: 23 | Naïve: 23 | SOF+Peg-IFN+RBV |
| Eric Lawitz 2015 [[51](#_ENREF_51)] | NA | Single-center | 47 | 56 | White: 45, Black: 1, Asian: 1 | Non-cirrhosis: 21, Cirrhosis26 | Experienced: 47 | SOF+Peg-IFN+RBV |
| Stefan Wirth 2017 [[52](#_ENREF_52)] | NCT02175758 | Multi-center | 52 | 15 | White: 47, Black: 2, Asian: 1, Pacific Islander: 1, Other: 1 | Non-cirrhosis: 21, Not Reported: 31 | Naïve: 43, Experienced: 9 | SOF+RBV |
| Mohan Kumar 2017 [[53](#_ENREF_53)] | NA | Multi-center | 82 | NA | NA | NA | NA | SOF+PegG-IFN+RBV |
| Mark S. Sulkowski 2014 [[54](#_ENREF_54)] | NCT01667731 | Multi-center | 223 | 49 | White: 156, Black: 52, Other: 15 | Non-cirrhosis: 201, Compensated cirrhosis: 22 | Naïve: 182, Experienced: 41 | SOF+RBV |
| Vasily Isakov 2018 [[55](#_ENREF_55)] | NCT02472886 | Multi-center | 126 | 38 | White: 125, Asian: 1 | Non-cirrhosis: 126 | Naïve: 126 | LDV/SOF |
| Susanna Naggie 2015 [[56](#_ENREF_56)] | NCT02073656 | Multi-center | 335 | 52 | White: 203, Black: 115, Asian: 6 | Non-cirrhosis: 259, Cirrhosis: 76 | Naïve: 150, Experienced: 185 | LDV/SOF |
| Eleanor M. Wilson 2016 [[57](#_ENREF_57)] | NCT01805882 | Single-center | 34 | 59 | White: 6, Black: 28 | Compensated cirrhosis: 33, Decompensated cirrhosis: 1 | Experienced: 34 | LDV/SOF |
| Anu Osinusi 2015 [[58](#_ENREF_58)] | NCT01878799 | Single-center | 50 | 58 | White:7, Blcak:1, Hispanic:42 | Non-cirrhosis: 50 | Naïve: 50 | LDV/SOF |
| El Khayat D.A.H. 2018 [[59](#_ENREF_59)] | NA | Multi-center | 144 | 14 | NA | NA | Naïve: 128, Experienced: 16 | LDV/SOF |
| Babatin M. 2017 [[60](#_ENREF_60)] | NA | NA | 45 | 44 | NA | Non-cirrhosis: 45 | Naïve: 45 | LDV/SOF |
| William F. Balistreri 2017 [[61](#_ENREF_61)] | EudraCT2014-003578-17 | Multi-center | 100 | 15 | White: 90, Black: 7, Asian:2, Not Reported: 1 | Non-cirrhosis: 42, Cirrhosis: 1, Not Reported: 57 | Naïve: 80, Experienced: 20 | LDV/SOF |
| Goki Suda 2017 [[62](#_ENREF_62)] | UMIN000020009 | Multi-center | 15 | 70 | Asian: 15 | Non-cirrhosis: 8, Cirrhosis: 7 | Experienced: 15 | LDV/SOF+RBV |
| Cooper C.L 2016 [[63](#_ENREF_63)] | NA | NA | 9 | NA | Black: 9 | Non-cirrhosis: 7, Cirrhosis: 2 | Experienced: 9 | LDV/SOF+RBV |
| Young-Suk Lim 2016 [[64](#_ENREF_64)] | NCT02021656 | Multi-center | 93 | 54 | Asian: 93 | Non-cirrhosis: 76, Compensated cirrhosis: 17 | Naïve: 46, Experienced: 47 | LDV/SOF |
| Etsuko Iio 2017 [[65](#_ENREF_65)] | NA | Multi-center | 523 | 70 | NA | Non-cirrhosis: 374, Cirrhosis: 149 | Experienced: 23, Naïve: 500 | LDV/SOF |
| V. D. Thong 2017 [[66](#_ENREF_66)] | NA | NA | 46 | NA | NA | Non-cirrhosis: 39, Decompensated cirrhosis: 7 | Naïve: 37, Experienced: 9 | LDV/SOF |
| Chun-Jen Liu 2018 [[67](#_ENREF_67)] | NCT02613871 | Multi-center | 111 | 55 | Asian: 111 | Non-cirrhosis: 93, Compensated cirrhosis: 18 | Naïve: 74, Experienced: 37 | LDV/SOF |
| Bekhbold Dashtseren 2017 [[68](#_ENREF_68)] | NA | NA | 119 | 54 | Asian: 119 | NA | NA | LDV/SOF |
| Anita Kohli 2015 [[69](#_ENREF_69)] | NCT01805882 | Single-center | 21 | 55 | White: 11, Black: 9, American Indian: 1 | Non-cirrhosis: 14, Compensated cirrhosis: 7 | Naïve: 13, Experienced: 8 | LDV/SOF |
| Armand Abergel 2016 [[70](#_ENREF_70)] | NCT02081079 | Multi-center | 44 | 51 | White: 36, Black: 8 | Non-cirrhosis: 34, Compensated cirrhosis: 10 | Naïve: 22, Experienced: 22 | LDV/SOF |
| A. Abergel 2016 [[71](#_ENREF_71)] | NCT02081079 | Multi-center | 41 | NA | White: 41 | Non-cirrhosis: 32, Compensated cirrhosis: 9 | Naïve: 21, Experienced: 20 | LDV/SOF |
| David Wyles 2015 [[72](#_ENREF_72)] | NCT01987453 | Multi-center | 51 | 54 | White: 43, Black: 8 | Non-cirrhosis: 37, Compensated cirrhosis: 14 | Experienced: 51 | LDV/SOF+RBV |
| Jordan J. Feld 2017 [[73](#_ENREF_73)] | NCT02413593 | Multi-center | 111 | 48 | White:78, Black:1, Asian:26, American Indian: 3, Other: 3 | Non-cirrhosis: 72, Compensated cirrhosis: 39 | NA | LDV/SOF+RBV |
| Wan-Long Chuang 2016 [[74](#_ENREF_74)] | NCT02021656 | Multi-center | 85 | 55 | Asian: 85 | Non-cirrhosis: 76, Compensated cirrhosis: 9 | Naïve: 42, Experienced: 43 | LDV/SOF |
| William Balistreri 2016 [[75](#_ENREF_75)] | NA | NA | 100 | 15 | NA | NA | NA | LDV/SOF |
| Lai Wei 2018 [[76](#_ENREF_76)] | NCT02021656 | Multi-center | 206 | 47 | Asian: 206 | Non-cirrhosis: 174, Compensated cirrhosis: 32 | Naïve: 106, Experienced: 100 | LDV/SOF |
| Lkhaasuren Nemekhbaatar 2017 [[77](#_ENREF_77)] | NA | NA | 1020 | NA | Asian: 1020 | NA | Naïve: 937, Experienced: 87 | LDV/SOF |
| Eric Lawitz 2017 [[78](#_ENREF_78)] | NA | NA | 18 | NA | Black: 10, Other: 4 | Non-cirrhosis: 16, Compensated cirrhosis: 2 | Naïve: 14, Experienced: 4 | LDV/SOF |
| Mindie H. Nguyen 2017 [[79](#_ENREF_79)] | NA | Multi-center | 60 | 58 | Asian: 60 | Non-cirrhosis: 33, Compensated cirrhosis: 25, Decompensated cirrhosis: 2 | Naïve: 45, Experienced: 15 | LDV/SOF |
| Patrick Basu P. 2017 [[80](#_ENREF_80)] | NA | Multi-center | 24 | NA | NA | NA | NA | LDV/SOF |
| Seng Gee Lim 2018 [[81](#_ENREF_81)] | NA | Multi-center | 111 | NA | NA | Non-cirrhosis: 95, Compensated cirrhosis: 16 | Naïve: 20, Experienced: 91 | SOF/VEL |
| David Wyles 2017 [[82](#_ENREF_82)] | NCT02480712 | Multi-center | 106 | 54 | White:54, Black:48, Asian:3, Other: 1 | Compensated cirrhosis: 19, Non-cirrhosis: 87 | Naïve: 75, Experienced: 31 | SOF/VEL |
| Lai Wei 2019* [[83](#_ENREF_83)] | NCT02671500 | Multi-center | 375 | 45 | NA | Non-cirrhosis: 308, Compensated cirrhosis: 67 | Naïve: 307, Experienced: 67 | SOF/VEL |
| Ajit Sood 2019 [[84](#_ENREF_84)] | NA | Multi-center | 129 | 42 | Asian: 129 | Non-cirrhosis: 87, Compensated cirrhosis: 42 | Naïve: 148, Experienced: 11 | SOF/VEL |
| Tetsuo Takehara 2022 [[85](#_ENREF_85)] | NCT04112303 | Multi-center | 37 | 65 | Asian: 37 | Compensated cirrhosis: 37 | Naïve: 29, Experienced: 8 | SOF/VEL |
| Vasily Isakov 2019 [[86](#_ENREF_86)] | NCT02722837 | Multi-center | 119 | 44 | White: 117, Asian: 2 | Non-cirrhosis: 97, Compensated cirrhosis: 22 | Naïve: 90, Experienced: 29 | SOF/VEL |
| Tarik Asselah 2019* [[87](#_ENREF_87)] | NCT02346721 | Multi-center | 111 | 54 | White:85, Black 12, Asian:11, Pacific Islander: 1, Others: 2 | Non-cirrhosis: 92, Cirrhosis: 19 | Naïve: 80, Experienced: 31 | SOF/VEL |
| Gane Edward J 2017 [[88](#_ENREF_88)] | NCT02300103 | Multi-center | 69 | 57 | White: 61, Black: 3, Asian: 2, Pacific Islander: 3 | Non-cirrhosis: 51, Cirrhosis18 | Experienced: 69 | SOF/VEL+RBV |
| Xiaoting Ye 2023 [[89](#_ENREF_89)] | NA | Single-center | 140 | NA | Asian: 140 | NA | NA | SOF/VEL |
| Kosh Agarwal 2018 [[90](#_ENREF_90)] | NCT02781571 | Multi-center | 79 | 62 | White: 65, Black: 2, Asian: 12 | Non-cirrhosis: 65, Compensated cirrhosis: 14 | Naïve: 32, Experienced: 47 | SOF/VEL |
| Sergio M. Borgia 2019 [[91](#_ENREF_91)] | NCT03036852 | Multi-center | 59 | 60 | White: 31, Black: 6, Asian: 18, American Indian: 2, Pacific Islander:2 | Non-cirrhosis: 42, Compensated cirrhosis: 17 | Naïve: 46, Experienced: 13 | SOF/VEL |
| Eleanor Wilson 2019 [[92](#_ENREF_92)] | NCT02745535 | Multi-center | 77 | 60 | White: 10, Black: 66, Other: 1 | Non-cirrhosis: 46, Compensated cirrhosis: 31 | Experienced: 77 | SOF/VEL/VOX |
| Marc Bourlière 2018 [[93](#_ENREF_93)] | NCT02607735 | Multi-center | 147 | 60 | White: 121, Black: 20, Asian: 6 | Non-cirrhosis: 98, Compensated cirrhosis: 49 | Experienced: 147 | SOF/VEL/VOX |
| Eric Lawitz 2016 [[94](#_ENREF_94)] | NA | NA | 128 | NA | White: 105 | Non-cirrhosis: 67, Cirrhosis: 61 | NA | SOF/VEL/VOX |
| Peter Ruane 2019 [[95](#_ENREF_95)] | NA | NA | 31 | NA | White: 25 | Non-cirrhosis: 16, Compensated cirrhosis: 15 | Experienced: 31 | SOF/VEL/VOX |

**Notes:** a: It is an organization; * means the same author and publication year, but different trail. *SATs* single-arm trials; *DAAs* direct-acting antiviral agents; *NA* not available; *AOF* alfosbuvir; *DAC* daclatasvir; *CLP* coblopasvir hydrochloride; *SOF* sofosbuvir; *DNV* danoprevir; *EMV* emitasvir phosphate; *RDV* ravidasvir; *EBR/GZR* elbasvir/grazoprevir; *GLE/PIB* glecaprevir/pibrentasvir; *LDV/SOF* ledipasvir/sofosbuvir; *SOF/VEL* sofosbuvir/velpatasvir; *SOF/VEL/VOX* sofosbuvir/velpatasvir/voxilaprevir; *Peg-IFN* pegylated interferon; *RBV* ribavirin.

**Table S5:** Risk of bias appraisal of individual RCTs

| Included study | Bias arising from the randomization process | Bias due to deviations from the intended interventions | Bias due to missing outcome data | Bias in measurement of the outcome | Bias in selection of the reported result | Overall assessment |
| --- | --- | --- | --- | --- | --- | --- |
| Gregory Everson 2014 [[1](#_ENREF_1)] | Low | Low | Low | Low | Low | Low |
| Xiaoyuan Xu 2019 [[2](#_ENREF_2)] | Low | Low | Low | Low | Low | Low |
| David Roth 2015 [[3](#_ENREF_3)] | Low | Low | Low | Low | Low | Low |
| Gregory J. Dore 2016 [[4](#_ENREF_4)] | Low | Low | Low | Low | Low | Low |
| Hiromitsu Kumada 2016 [[5](#_ENREF_5)] | Some concerns | Low | Low | Low | Low | Some concerns |
| Stefan Zeuzem 2015 [[6](#_ENREF_6)] | Low | Low | Low | Low | Low | Low |
| Wei Lai 2019 [[7](#_ENREF_7)] | Low | Low | Low | Low | Low | Low |
| Hézode 2016 [[8](#_ENREF_8)] | Some concerns | Low | Low | Low | Low | Some concerns |
| Tarik Asselah 2017 [[9](#_ENREF_9)] | Some concerns | Low | Low | Low | Low | Some concerns |
| Wei Lai 2020 [[10](#_ENREF_10)] | Low | Low | Low | Low | Low | Low |
| Marc Bourlière 2015 [[11](#_ENREF_11)] | Some concerns | Low | Low | Low | Low | Some concerns |
| Eric Lawitz 2013* [[12](#_ENREF_12)] | Some concerns | Some concerns | Low | Low | Low | Some concerns |
| Ira M. Jacobson 2013 [[13](#_ENREF_13)] | Some concerns | Low | Low | Low | Low | Some concerns |
| YANG Yong-rui 2020 [[14](#_ENREF_14)] | Some concerns | Low | Low | Low | Low | Some concerns |
| Eric Lawitz 2013 [[15](#_ENREF_15)] | Low | Low | Low | Low | Low | Low |
| Fu Xiaoyi 2018 [[16](#_ENREF_16)] | High | Some concerns | Low | Low | Low | High |
| Huang Maohui 2022 [[17](#_ENREF_17)] | Low | Some concerns | Low | Low | Low | Some concerns |
| J.J. Feld 2015 [[18](#_ENREF_18)] | Some concerns | Low | Low | Low | Low | Some concerns |
| M. Bourlière 2017 [[19](#_ENREF_19)] | Low | Low | Low | Low | Low | Low |

Notes: * means the same author and publication year, but different trail; *RCTs* randomized controlled trials. Version 2 of the Cochrane risk-of-bias tool for RCTs considers five aspects of bias, including bias arising from the randomization process, deviations of the intended interventions, missing outcome data, measurement of the outcome and selection of the reported result. The overall quality was rated as with ‘low risk of bias’, ‘some concerns’, or ‘high risk of bias’ respectively. Only all of the five aspects were assessed as with ‘low risk of bias’, the overall quality could be evaluated as with ‘low risk of bias’. When one or two aspects were assessed as with ‘some concerns’, the overall quality was evaluated as with ‘some concerns’. When more than two aspects were assessed as ‘with some concerns’ or more than one aspects were assessed as with ‘high risk of bias’, the overall quality was evaluated as with ‘high risk of bias’.

**Table S6:** Risk of bias appraisal of individual SATs

| Included study | Criterion | | | | | | | | | | | | | | | | | | | | Overall score | Overall assessment |
| --- | --- | --- | --- | --- | --- | --- | --- | --- | --- | --- | --- | --- | --- | --- | --- | --- | --- | --- | --- | --- | --- | --- |
|  | 1 | 2 | 3 | 4 | 5 | 6 | 7 | 8 | 9 | 10 | 11 | 12 | 13 | 14 | 15 | 16 | 17 | 18 | 19 | 20 |  |  |
| Rui Hua 2023 [[20](#_ENREF_20)] | Yes | Yes | Yes | Yes | Yes | Yes | Yes | Yes | No | Yes | Yes | Yes | Yes | Yes | Yes | Yes | Yes | Yes | Yes | Yes | 19 | Low |
| Yanhang Gao 2020 [[21](#_ENREF_21)] | Yes | Yes | Yes | Yes | Yes | Yes | Yes | Yes | No | Yes | Yes | Yes | Yes | Yes | Yes | Yes | Yes | Yes | Yes | Yes | 19 | Low |
| Sujun Zheng 2017 [[22](#_ENREF_22)] | Yes | Yes | Yes | Unclear | Partial | Partial | Yes | Yes | Yes | Yes | Yes | Yes | Yes | Unclear | Yes | Yes | Yes | Yes | Yes | No | 17 | Low |
| Lai Wei 2019 [[23](#_ENREF_23)] | Yes | Yes | Yes | Yes | Yes | Yes | Yes | Yes | Yes | Yes | Yes | Yes | Yes | Unclear | Yes | Yes | Yes | Yes | Yes | Yes | 19.5 | Low |
| Jia-Horng Kao 2016 [[24](#_ENREF_24)] | Yes | Yes | Yes | Yes | Yes | Yes | No | Yes | Yes | Yes | Yes | Yes | Yes | Yes | Yes | Yes | Yes | Yes | Yes | Yes | 19 | Low |
| Huiying Rao 2020 [[25](#_ENREF_25)] | Yes | Yes | Yes | Unclear | Yes | Yes | Yes | Yes | No | Yes | Yes | Yes | Yes | Unclear | Yes | Yes | Yes | Yes | Yes | Yes | 18 | Low |
| Jia-Horng Kao 2018 [[26](#_ENREF_26)] | Yes | Yes | Yes | Yes | Yes | Yes | Yes | Yes | No | Yes | Yes | Yes | Yes | Unclear | Yes | Yes | Yes | Yes | Yes | Yes | 18.5 | Low |
| Hiromitsu Kumada 2016 [[5](#_ENREF_5)] | Yes | Yes | Yes | Yes | Yes | Yes | Yes | Yes | No | Yes | Yes | Yes | Yes | Yes | Yes | Yes | Yes | Yes | Yes | Yes | 19 | Low |
| Anne Boerekamps 2019 [[27](#_ENREF_27)] | Yes | Yes | Yes | Yes | Partial | Yes | Yes | Yes | Yes | Yes | Yes | Yes | Yes | Unclear | Yes | Yes | Yes | Yes | Yes | Yes | 19 | Low |
| Tarik Asselah 2017 [[9](#_ENREF_9)] | Yes | Yes | Yes | Yes | Yes | Yes | Yes | Yes | No | Yes | Yes | Yes | Yes | Yes | Yes | Yes | Yes | Yes | Yes | Yes | 19 | Low |
| Ahmad AlEid 2022 [[28](#_ENREF_28)] | Yes | Yes | No | Yes | Partial | Yes | Unclear | Yes | Yes | Yes | Yes | Yes | Yes | Yes | Yes | Yes | Yes | Yes | Yes | Yes | 18 | Low |
| Armand Abergel 2020 [[29](#_ENREF_29)] | Yes | Yes | Yes | Yes | Partial | Yes | Yes | Yes | No | Yes | Yes | Yes | Yes | Unclear | Yes | Yes | Yes | Yes | Yes | Yes | 18 | Low |
| Jürgen K Rockstroh 2015 [[30](#_ENREF_30)] | Yes | Yes | Yes | Yes | Partial | Yes | Yes | Yes | Yes | Yes | Yes | Yes | Yes | Yes | Yes | Yes | Yes | Yes | Yes | Yes | 19.5 | Low |
| Maria Buti 2016 [[31](#_ENREF_31)] | Yes | Yes | Yes | Unclear | Yes | Yes | No | Yes | No | Yes | Yes | Yes | Yes | Unclear | Yes | Yes | Yes | Yes | Yes | Yes | 17 | Low |
| Tarik Asselah 2017* [[9](#_ENREF_9)] | Yes | Yes | Yes | Yes | Yes | Yes | Yes | Yes | No | Yes | Yes | Yes | Yes | Yes | Yes | Yes | Yes | Yes | Yes | Yes | 19 | Low |
| Xavier Forns 2017 [[32](#_ENREF_32)] | Yes | Yes | Yes | Yes | Yes | Yes | Yes | Yes | Yes | Yes | Yes | Yes | Yes | Yes | Yes | Yes | Yes | Yes | Yes | Yes | 20 | Low |
| Nancy Reau 2018 [[33](#_ENREF_33)] | Yes | Yes | Yes | Yes | Yes | Yes | Yes | Yes | No | Yes | Yes | Yes | Yes | Unclear | Yes | Yes | Yes | Yes | Yes | Yes | 18.5 | Low |
| Masanori Atsukawa 2019 [[34](#_ENREF_34)] | Yes | Yes | Yes | Yes | Yes | Yes | Yes | Yes | Yes | Yes | Yes | Yes | Yes | Yes | Yes | Yes | Yes | Yes | Yes | Yes | 20 | Low |
| Tarik Asselah 2019 [[35](#_ENREF_35)] | Yes | Yes | Yes | Yes | Yes | Yes | Yes | Yes | Yes | Yes | Yes | Yes | Yes | Yes | Yes | Yes | Yes | Yes | Yes | Yes | 20 | Low |
| Eric Lawitz 2019 [[36](#_ENREF_36)] | Yes | Yes | Yes | Yes | Yes | Yes | Yes | Yes | No | Yes | Yes | Yes | Yes | Unclear | Yes | Yes | Yes | Yes | Yes | Yes | 18.5 | Low |
| Kovesdy C.P 2017 [[37](#_ENREF_37)] | Yes | Yes | Unclear | Unclear | Partial | Partial | Yes | Yes | Yes | Yes | Yes | Yes | Yes | Unclear | Yes | Yes | Yes | Yes | Yes | Partial | 17 | Low |
| Robert S. Brown 2020 [[38](#_ENREF_38)] | Yes | Yes | Yes | Yes | Yes | Yes | Yes | Yes | No | Yes | Yes | Yes | Yes | Yes | Yes | Yes | Yes | Yes | Yes | Yes | 19 | Low |
| Lai Wei 2020 [[10](#_ENREF_10)] | Yes | Yes | Yes | Yes | Yes | Yes | Yes | Yes | No | Yes | Yes | Yes | Yes | Yes | Yes | Yes | Yes | Yes | Yes | Yes | 19 | Low |
| Mario Peribañez-Gonzalez 2020 [[39](#_ENREF_39)] | Yes | Yes | Yes | Yes | Yes | Yes | No | Yes | Yes | Yes | Yes | Yes | Yes | Unclear | Yes | Yes | Yes | Yes | Yes | Yes | 18.5 | Low |
| Jia-Horng Kao 2016* [[40](#_ENREF_40)] | Yes | Yes | Yes | Yes | Yes | Yes | Yes | Yes | No | Yes | Yes | Yes | Yes | Unclear | Yes | Yes | Yes | Yes | Yes | Yes | 18.5 | Low |
| S. H. Ahn 2016 [[41](#_ENREF_41)] | Yes | Yes | Yes | Yes | Yes | Yes | Yes | Yes | Yes | Yes | Yes | Yes | Yes | Yes | Yes | Yes | Yes | Yes | Yes | Yes | 20 | Low |
| KHALID MAHMUD KHAN 2017 [[42](#_ENREF_42)] | Yes | Yes | Yes | Unclear | Partial | No | Unclear | Yes | Yes | Yes | Yes | Yes | Yes | Unclear | Yes | Yes | Yes | Yes | Yes | No | 16 | Some concerns |
| Rui Huang 2019 [[43](#_ENREF_43)] | Yes | Yes | Yes | Yes | Yes | Yes | Yes | Yes | No | Yes | Yes | Yes | Yes | Yes | Yes | Yes | Yes | Yes | Yes | Yes | 19 | Low |
| Fumimasa Tomooka 2017 [[44](#_ENREF_44)] | Yes | Yes | Unclear | Unclear | Partial | Partial | Unclear | Yes | Yes | Yes | Yes | Yes | Yes | Unclear | Yes | No | Yes | No | Yes | No | 14 | Some concerns |
| Michael Charlton 2015 [[45](#_ENREF_45)] | Yes | Yes | Yes | Yes | Yes | Yes | No | Yes | No | Yes | Yes | Yes | Yes | Unclear | Yes | Yes | Yes | Yes | Yes | Yes | 17.5 | Low |
| Michael P. Curry 2015 [[46](#_ENREF_46)] | Yes | Yes | Yes | Yes | Yes | Yes | Yes | Yes | Yes | Yes | Yes | Yes | Yes | Yes | Yes | Yes | Yes | Yes | Yes | Yes | 20 | Low |
| Stanislas Pol 2015 [[47](#_ENREF_47)] | Yes | Yes | Yes | Unclear | Yes | Yes | Yes | Yes | Yes | Yes | Yes | Yes | Yes | Yes | Yes | Yes | Yes | Yes | Yes | Yes | 19.5 | Low |
| Sherief Abd-Elsalam 2016 [[48](#_ENREF_48)] | Yes | Yes | Unclear | Unclear | Partial | Partial | Yes | Yes | Yes | Yes | Yes | Yes | Yes | Unclear | Yes | Yes | Yes | Yes | Yes | No | 16.5 | Low |
| Masao Omata 2014 [[49](#_ENREF_49)] | Yes | Yes | Yes | Yes | Yes | Yes | Yes | Yes | Yes | Yes | Yes | Yes | Yes | Yes | Yes | Yes | Yes | Yes | Yes | Yes | 20 | Low |
| Eric Lawitz 2013 [[15](#_ENREF_15)] | Yes | Yes | Yes | Yes | Yes | Yes | Yes | Yes | Yes | Yes | Yes | Yes | Yes | Unclear | Yes | Yes | Yes | Yes | Yes | Yes | 19.5 | Low |
| Eric Lawitz 2013* [[12](#_ENREF_12)] | Yes | Yes | Yes | Yes | Yes | Yes | Yes | Yes | Yes | Yes | Yes | Yes | Yes | Yes | Yes | Yes | Yes | Yes | Yes | Yes | 20 | Low |
| Maribel Rodriguez-Torres 2015 [[50](#_ENREF_50)] | Yes | Yes | No | Yes | Yes | Yes | Yes | Yes | Yes | Yes | Yes | Yes | Yes | Yes | Yes | Yes | Yes | Yes | Yes | Yes | 19 | Low |
| Eric Lawitz 2015 [[51](#_ENREF_51)] | Yes | Yes | No | Yes | Yes | Yes | No | Yes | Yes | Yes | Yes | Yes | Yes | Yes | Yes | Yes | Yes | Yes | Yes | Yes | 18 | Low |
| Stefan Wirth 2017 [[52](#_ENREF_52)] | Yes | Yes | Yes | Yes | Yes | Yes | Unclear | Yes | Yes | Yes | Yes | Yes | Yes | Yes | Yes | Yes | Yes | Yes | Yes | Yes | 19.5 | Low |
| Mohan Kumar 2017 [[53](#_ENREF_53)] | Yes | Yes | Yes | Unclear | Partial | No | Unclear | Yes | Yes | Yes | Yes | Yes | Yes | Unclear | Yes | Yes | Yes | No | Yes | No | 15 | Some concerns |
| Mark S. Sulkowski 2014 [[54](#_ENREF_54)] | Yes | Yes | Yes | Yes | Yes | Yes | Yes | Yes | Yes | Yes | Yes | Yes | Yes | Yes | Yes | Yes | Yes | Yes | Yes | Partial | 19.5 | Low |
| Vasily Isakov 2018 [[55](#_ENREF_55)] | Yes | Yes | Yes | Yes | Yes | Yes | Yes | Yes | Yes | Yes | Yes | Yes | Yes | Yes | Yes | Yes | Yes | Yes | Yes | Yes | 20 | Low |
| Susanna Naggie 2015 [[56](#_ENREF_56)] | Yes | Yes | Yes | Yes | Yes | Yes | No | Yes | Yes | Yes | Yes | Yes | Yes | Yes | Yes | Yes | Yes | Yes | Yes | Yes | 19 | Low |
| Eleanor M. Wilson 2016 [[57](#_ENREF_57)] | Yes | Yes | No | Yes | Yes | Yes | Yes | Yes | Yes | Yes | Yes | Yes | Yes | Yes | Yes | Yes | Yes | Yes | Yes | Yes | 19 | Low |
| Anu Osinusi 2015 [[58](#_ENREF_58)] | Yes | Yes | No | Yes | Yes | Yes | Yes | Yes | Yes | Yes | Yes | Yes | Yes | Yes | Yes | Yes | Yes | Yes | Yes | Yes | 19 | Low |
| El Khayat D.A.H. 2018 [[59](#_ENREF_59)] | Yes | Yes | Yes | No | Partial | Partial | Unclear | Yes | Yes | Yes | Yes | Yes | Yes | Unclear | Yes | No | Yes | Yes | Yes | No | 15 | Some concerns |
| Babatin M. 2017 [[60](#_ENREF_60)] | Yes | Yes | Unclear | No | Partial | Partial | Yes | Yes | No | Yes | Yes | Yes | Yes | Unclear | Yes | Yes | Yes | Yes | Yes | No | 15 | Some concerns |
| William F. Balistreri 2017 [[61](#_ENREF_61)] | Yes | Yes | Yes | Yes | Yes | Yes | No | Yes | Yes | Yes | Yes | Yes | Yes | Yes | Yes | Yes | Yes | Yes | Yes | Yes | 19 | Low |
| Goki Suda 2017 [[62](#_ENREF_62)] | Yes | Yes | Yes | Yes | Yes | Yes | No | Yes | Yes | Yes | Yes | Yes | Yes | Unclear | Yes | Yes | Yes | Yes | Yes | Yes | 18.5 | Low |
| Cooper C.L 2016 [[63](#_ENREF_63)] | Yes | Yes | Unclear | Unclear | Partial | Partial | Yes | Yes | Yes | Yes | Yes | Yes | Yes | Unclear | Yes | Yes | Yes | Yes | Yes | No | 16.5 | Low |
| Young-Suk Lim 2016 [[64](#_ENREF_64)] | Yes | Yes | Yes | Yes | Yes | Yes | Yes | Yes | Yes | Yes | Yes | Yes | Yes | Yes | Yes | Yes | Yes | Yes | Yes | Yes | 20 | Low |
| Etsuko Iio 2017 [[65](#_ENREF_65)] | Yes | Yes | Yes | Unclear | Partial | Partial | No | Yes | Yes | Yes | Yes | Yes | Yes | Yes | Yes | Yes | Yes | Yes | Yes | No | 16.5 | Low |
| V. D. Thong 2017 [[66](#_ENREF_66)] | Yes | Yes | Unclear | Unclear | Partial | Partial | Yes | Yes | Yes | Yes | Yes | Yes | Yes | Unclear | Yes | Yes | Yes | Yes | Yes | No | 16.5 | Low |
| Chun-Jen Liu 2018 [[67](#_ENREF_67)] | Yes | Yes | Yes | Yes | Yes | Yes | No | Yes | Yes | Yes | Yes | Yes | Yes | Unclear | Yes | Yes | Yes | Yes | Yes | Yes | 18.5 | Low |
| Bekhbold Dashtseren 2017 [[68](#_ENREF_68)] | Yes | Yes | Unclear | Unclear | Partial | Partial | Unclear | Yes | Yes | Yes | Yes | Yes | Yes | Unclear | Yes | Yes | Yes | Yes | Yes | No | 16 | Some concerns |
| Anita Kohli 2015 [[69](#_ENREF_69)] | Yes | Yes | Yes | Yes | Yes | Yes | No | Yes | Yes | Yes | Yes | Yes | Yes | Unclear | Yes | Yes | Yes | Yes | Yes | No | 17.5 | Low |
| Armand Abergel 2016 [[70](#_ENREF_70)] | Yes | Yes | Yes | Yes | Yes | Yes | No | Yes | Yes | Yes | Yes | Yes | Yes | Yes | Yes | Yes | Yes | Yes | Yes | Partial | 18.5 | Low |
| A. Abergel 2016 [[71](#_ENREF_71)] | Yes | Yes | Yes | Yes | Yes | Yes | Yes | Yes | Yes | Yes | Yes | Yes | Yes | Yes | Yes | Yes | Yes | Yes | Yes | Yes | 20 | Low |
| David Wyles 2015 [[72](#_ENREF_72)] | Yes | Yes | Yes | Yes | Yes | Yes | No | Yes | Yes | Yes | Yes | Yes | Yes | Yes | Yes | Yes | Yes | Yes | Yes | Yes | 19 | Low |
| Jordan J. Feld 2017 [[73](#_ENREF_73)] | Yes | Yes | Yes | Yes | Yes | Yes | No | Yes | Yes | Yes | Yes | Yes | Yes | Yes | Yes | Yes | Yes | Yes | Yes | Yes | 19 | Low |
| Wan-Long Chuang 2016 [[74](#_ENREF_74)] | Yes | Yes | Yes | Yes | Yes | Yes | Yes | Yes | Yes | Yes | Yes | Yes | Yes | Unclear | Yes | Yes | Yes | Yes | Yes | Yes | 19.5 | Low |
| William Balistreri 2016 [[75](#_ENREF_75)] | Yes | Yes | Unclear | Unclear | Partial | Partial | Unclear | Yes | Yes | Yes | Yes | Yes | Yes | Unclear | Yes | Yes | Yes | Yes | Yes | No | 16 | Some concerns |
| Lai Wei 2018 [[76](#_ENREF_76)] | Yes | Yes | Yes | Yes | Yes | Yes | Yes | Yes | Yes | Yes | Yes | Yes | Yes | Yes | Yes | Yes | Yes | Yes | Yes | Yes | 20 | Low |
| Lkhaasuren Nemekhbaatar 2017 [[77](#_ENREF_77)] | Yes | Yes | Unclear | Unclear | Partial | Partial | Unclear | Yes | Yes | Yes | Yes | Yes | Yes | Unclear | Yes | No | Yes | Yes | Yes | No | 15 | Some concerns |
| Eric Lawitz 2017 [[78](#_ENREF_78)] | Yes | Yes | Unclear | Unclear | Partial | Partial | Yes | Yes | Yes | Yes | Yes | Yes | Yes | Unclear | Yes | Yes | Yes | Yes | Yes | No | 16.5 | Low |
| Mindie H. Nguyen 2017 [[79](#_ENREF_79)] | Yes | Yes | Yes | Unclear | Yes | Partial | No | Yes | Yes | Yes | Yes | Yes | Yes | Unclear | Yes | Yes | Yes | Yes | Yes | No | 16.5 | Low |
| Patrick Basu P. 2017 [[80](#_ENREF_80)] | Yes | Yes | Yes | Unclear | Partial | Partial | Unclear | Yes | Yes | Yes | Yes | Yes | Yes | Unclear | Yes | No | Yes | No | Yes | No | 14.5 | Some concerns |
| Seng Gee Lim 2018 [[81](#_ENREF_81)] | Yes | Yes | Yes | Unclear | Partial | Partial | Yes | Yes | Yes | Yes | Yes | Yes | Yes | Unclear | Yes | Yes | Yes | Yes | Yes | No | 17 | Low |
| David Wyles 2017 [[82](#_ENREF_82)] | Yes | Yes | Yes | Yes | Yes | Yes | Yes | Yes | Yes | Yes | Yes | Yes | Yes | Yes | Yes | Yes | Yes | Yes | Yes | Yes | 20 | Low |
| Lai Wei 2019* [[83](#_ENREF_83)] | Yes | Yes | Yes | Yes | Yes | Yes | Yes | Yes | Yes | Yes | Yes | Yes | Yes | Yes | Yes | Yes | Yes | Yes | Yes | Yes | 20 | Low |
| Ajit Sood 2019 [[84](#_ENREF_84)] | Yes | Yes | Yes | Yes | Yes | Yes | No | Yes | Yes | Yes | Yes | Yes | Yes | Yes | Yes | Yes | Yes | Yes | Yes | Yes | 19 | Low |
| Tetsuo Takehara 2022 [[85](#_ENREF_85)] | Yes | Yes | Yes | Yes | Yes | Yes | Yes | Yes | Yes | Yes | Yes | Yes | Yes | Yes | Yes | Yes | Yes | Yes | Yes | Yes | 20 | Low |
| Vasily Isakov 2019 [[86](#_ENREF_86)] | Yes | Yes | Yes | Yes | Yes | Yes | Yes | Yes | Yes | Yes | Yes | Yes | Yes | Unclear | Yes | Yes | Yes | Yes | Yes | Yes | 19.5 | Low |
| Tarik Asselah 2019* [[87](#_ENREF_87)] | Yes | Yes | Yes | Yes | Yes | Yes | Yes | Yes | Yes | Yes | Yes | Yes | Yes | Yes | Yes | Yes | Yes | Yes | Yes | Yes | 20 | Low |
| Gane Edward J 2017 [[88](#_ENREF_88)] | Yes | Yes | Yes | Yes | Yes | Yes | No | Yes | Yes | Yes | Yes | Yes | Yes | Yes | Yes | Yes | Yes | Yes | Yes | Yes | 19 | Low |
| Xiaoting Ye 2023 [[89](#_ENREF_89)] | Yes | Yes | No | Unclear | Partial | Partial | Unclear | Yes | Yes | Yes | Yes | Yes | Yes | Unclear | Yes | Yes | Yes | No | Yes | No | 14.5 | Some concerns |
| Kosh Agarwal 2018 [[90](#_ENREF_90)] | Yes | Yes | Yes | Unclear | Yes | Yes | Yes | Yes | Yes | Yes | Yes | Yes | Yes | Unclear | Yes | Yes | Yes | Yes | Yes | Partial | 18.5 | Low |
| Sergio M. Borgia 2019 [[91](#_ENREF_91)] | Yes | Yes | Yes | Yes | Yes | Yes | Yes | Yes | Yes | Yes | Yes | Yes | Yes | Yes | Yes | Yes | Yes | Yes | Yes | Yes | 20 | Low |
| Eleanor Wilson 2019 [[92](#_ENREF_92)] | Yes | Yes | Yes | Yes | Yes | Yes | No | Yes | No | Yes | Yes | Yes | Yes | Yes | Yes | Yes | Yes | Yes | Yes | Yes | 18 | Low |
| Marc Bourlière 2018 [[93](#_ENREF_93)] | Yes | Yes | Yes | Yes | Yes | Yes | Yes | Yes | Yes | Yes | Yes | Yes | Yes | Yes | Yes | Yes | Yes | Yes | Yes | Yes | 20 | Low |
| Eric Lawitz 2016 [[94](#_ENREF_94)] | Yes | Yes | Unclear | Unclear | Partial | Partial | No | Yes | Yes | Yes | Yes | Yes | Yes | Unclear | Yes | Yes | Yes | Yes | Yes | No | 15.5 | Some concerns |
| Peter Ruane 2019 [[95](#_ENREF_95)] | Yes | Yes | Unclear | Unclear | Partial | Yes | Yes | Yes | Yes | Yes | Yes | Yes | Yes | Yes | Yes | Yes | Yes | Yes | Yes | Yes | 18.5 | Low |

**Notes:** * means the same author and publication year, but different trail; *SATs* single-arm trials; Criterion 1: Was the hypothesis/aim/objective of the study clearly stated? Criterion 2: Was the study conducted prospectively? Criterion 3: Were the cases collected in more than one center? Criterion 4: Were patients recruited consecutively? Criterion 5: Were the characteristics of the patients included in the study described? Criterion 6: Were the eligibility criteria for entry into the study clearly stated? Criterion 7: Did patients enter the study at a similar point in the disease? Criterion 8: Was the intervention of interest clearly described? Criterion 9: Were additional interventions (co-interventions) clearly described? Criterion 10: Were relevant outcome measures established a priori? Criterion 11: Were outcome assessors blinded to the intervention that patients received? (Answer yes when blinding is not applicable or is unnecessary) Criterion 12: Were the relevant outcomes measured using appropriate objective/subjective methods? Criterion 13: Were the relevant outcome measures made before and after the intervention? Criterion 14: Were the statistical tests used to assess the relevant outcomes appropriate? Criterion 15: Was follow-up long enough for important events and outcomes to occur? Criterion 16: Were losses to follow-up reported? Criterion 17: Did the study provided estimates of random variability in the data analysis of relevant outcomes? Criterion 18: Were the adverse events reported? Criterion 19: Were the conclusions of the study supported by the results? Criterion 20: Were both competing interests and sources of support for the study reported? The check list of the Institute of Health Economics consists of 20 criteria. Each criterion was assessed as ‘yes’, ‘no’, ‘partial’ or ‘unclear’. We assigned 1 point for ‘yes’, 0.5 points for ‘partial/unclear’, and 0 points for ’no’. The full score is 20 points if all criteria are assessed as ‘yes’. Following the method adopted by previous studies for making the overall quality assessment, we assessed the study as with ‘low risk of bias’ when the overall score reached more than 80% of the full score (> 16 out of 20 points for this case), ‘some concern’ when the overall score was between 50% and 80% of the full score (10 to 16 out of 20 points), and ‘high risk’ when the overall score was less than 50% of the full score (< 10 out of 20 points).

Figure S2: Summary of overall risk of bias of individual RCTs

Figure S3: Summary of overall risk of bias of individual SATs

**Notes:** Criterion 1: Was the hypothesis/aim/objective of the study clearly stated? Criterion 2: Was the study conducted prospectively? Criterion 3: Were the cases collected in more than one center? Criterion 4: Were patients recruited consecutively? Criterion 5: Were the characteristics of the patients included in the study described? Criterion 6: Were the eligibility criteria for entry into the study clearly stated? Criterion 7: Did patients enter the study at a similar point in the disease? Criterion 8: Was the intervention of interest clearly described? Criterion 9: Were additional interventions (co-interventions) clearly described? Criterion 10: Were relevant outcome measures established a priori? Criterion 11: Were outcome assessors blinded to the intervention that patients received? (Answer yes when blinding is not applicable or is unnecessary) Criterion 12: Were the relevant outcomes measured using appropriate objective/subjective methods? Criterion 13: Were the relevant outcome measures made before and after the intervention? Criterion 14: Were the statistical tests used to assess the relevant outcomes appropriate? Criterion 15: Was follow-up long enough for important events and outcomes to occur? Criterion 16: Were losses to follow-up reported? Criterion 17: Did the study provided estimates of random variability in the data analysis of relevant outcomes? Criterion 18: Were the adverse events reported? Criterion 19: Were the conclusions of the study supported by the results? Criterion 20: Were both competing interests and sources of support for the study reported? The check list of the Institute of Health Economics consists of 20 criteria. Each criterion was assessed as ‘yes’, ‘no’, ‘partial’ or ‘unclear’. We assigned 1 point for ‘yes’, 0.5 points for ‘partial/unclear’, and 0 points for ’no’. The full score is 20 points if all criteria are assessed as ‘yes’. Following the method adopted by previous studies for making the overall quality assessment, we assessed the study as with ‘low risk of bias’ when the overall score reached more than 80% of the full score (> 16 out of 20 points for this case), ‘some concern’ when the overall score was between 50% and 80% of the full score (10 to 16 out of 20 points), and ‘high risk’ when the overall score was less than 50% of the full score (< 10 out of 20 points).

Table S7: Subgroup analyses of RCTs with Peg-IFN+RBV as the control for safety outcome measures: locally developed vs imported DAAs

| Outcome measure | Subgroup | Origin of DAA | No. of included studies | Experimental group | Control group | Heterogeneity | | RD (95% *CI*) | *P*-value |
| --- | --- | --- | --- | --- | --- | --- | --- | --- | --- |
|  |  |  |  | No. of events/sample size | | *I^2^*(%) | *P* |  |  |
| AEs | Ethnicity | White and Black | 2 | 310/349 | 275/287 |  |  |  |  |
|  |  | Locally developed | 1 | 90/93 | 42/44 | / | / | 0.01 (-0.06, 0.08) | **0.01** |
|  |  | Imported | 1 | 220/256 | 233/243 | / | / | -0.10 (-0.15, -0.05) |  |
|  | Treatment experience | Treatment-naïve | 3 | 347/395 | 320/333 |  |  |  |  |
|  |  | Locally developed | 1 | 90/93 | 42/44 | / | / | 0.01 (-0.06, 0.08) | **0.01** |
|  |  | Imported | 2 | 257/302 | 278/289 | 0.00 | 0.97 | -0.11 (-0.17, -0.06) |  |
| SAEs | Ethnicity | White and Black | 2 | 15/349 | 4/287 |  |  |  |  |
|  |  | Locally developed | 1 | 8/93 | 1/44 | / | / | 0.06 (-0.01, 0.14) | 0.21 |
|  |  | Imported | 1 | 7/256 | 3/243 | / | / | 0.01 (-0.01, 0.04) |  |
|  | Treatment experience | Treatment-naïve | 3 | 15/395 | 4/333 |  |  |  |  |
|  |  | Locally developed | 1 | 8/93 | 1/44 | / | / | 0.06 (-0.009, 0.14) | 0.10 |
|  |  | Imported | 2 | 7/302 | 3/289 | / | 1.00 | 0.002 (-0.008, 0.01) |  |

**Notes:** *RCTs* randomized controlled trials; *Peg-IFN+RBV* pegylated interferon and ribavirin; *DAAs* direct-acting antiviral agents; *RD* risk difference; *CI* confidence interval; *AEs* any adverse events; *SAEs* serious adverse events; *P*-value for test of group difference; Bold means statistically significant; Heterogeneity is not reported in case that the number of trials ≤ 3.

Table S8: Subgroup analyses of RCTs with placebo as the control for safety outcome measures: locally developed vs imported DAAs

| Outcome measure | Subgroup | Origin of DAA | No. of included studies | Experimental group | Control group | Heterogeneity | | RD (95% *CI*) | *P*-value |
| --- | --- | --- | --- | --- | --- | --- | --- | --- | --- |
|  |  |  |  | No. of events/sample size | | *I^2^*(%) | *P* |  |  |
| AEs | Ethnicity | Asian | 4 | 806/1272 | 290/486 |  |  |  |  |
|  |  | Locally developed | 1 | 298/318 | 84/106 | / | / | 0.14 (0.06, 0.23) | **0.002** |
|  |  | Imported | 3 | 508/954 | 206/380 | 0.00 | 0.86 | -0.02 (-0.08, 0.04) |  |
|  | Cirrhosis status | Non-cirrhotic | 4 | 751/1109 | 286/463 |  |  |  |  |
|  |  | Locally developed | 1 | 298/318 | 84/106 | / | / | 0.14 (0.06, 0.23) | **0.01** |
|  |  | Imported | 3 | 453/791 | 202/357 | 0.01 | 0.38 | -0.003 (-0.06, 0.06) |  |
|  | Treatment experience | Treatment-naïve | 4 | 863/1200 | 301/434 |  |  |  |  |
|  |  | Locally developed | 1 | 298/318 | 84/106 | / | / | 0.14 (0.06, 0.23) | **0.003** |
|  |  | Imported | 3 | 565/882 | 217/328 | 0.00 | 0.97 | -0.003 (-0.06, 0.05) |  |
| SAEs | Ethnicity | Asian | 4 | 26/1272 | 11/486 |  |  |  |  |
|  |  | Locally developed | 1 | 7/318 | 5/106 | / | / | -0.03 (-0.07, 0.02) | 0.25 |
|  |  | Imported | 3 | 19/954 | 6/380 | 57.50 | 0.15 | 0.004 (-0.02, 0.03) |  |
|  | Cirrhosis status | Non-cirrhotic | 4 | 24/1109 | 10/463 |  |  |  |  |
|  |  | Locally developed | 1 | 7/318 | 5/106 | / | / | -0.03 (-0.07, 0.02) | 0.21 |
|  |  | Imported | 3 | 17/791 | 5/357 | 53.33 | 0.15 | 0.006 (-0.02, 0.03) |  |
|  | Treatment experience | Treatment-naïve | 4 | 28/1200 | 14/434 |  |  |  |  |
|  |  | Locally developed | 1 | 7/318 | 5/106 | / | / | -0.03 (-0.07, 0.02) | 0.35 |
|  |  | Imported | 3 | 21/882 | 9/328 | 0.05 | 0.99 | -0.002 (-0.02, 0.02) |  |

**Notes:** *RCTs* randomized controlled trials; *DAAs* direct-acting antiviral agents; *RD* risk difference; *CI* confidence interval; *AEs* any adverse events; *SAEs* serious adverse events; *P*-value for test of group difference; Bold means statistically significant; Heterogeneity is not reported in case that the number of trials ≤ 3.

Table S9: Subgroup analyses of SATs for efficacy outcome measures: locally developed vs imported DAAs

| Outcome measure | Subgroup | Origin of DAA | No. of included studies | No. of events/sample size | Heterogeneity | | ES (95% *CI*) | *P*-value |
| --- | --- | --- | --- | --- | --- | --- | --- | --- |
|  |  |  |  |  | *I^2^*(%) | *P* |  |  |
| SVR12 | Ethnicity | Asian | 24 | 3995/4089 |  |  |  |  |
|  |  | Locally developed | 7 | 1335/1369 | 79.01 | 0.00 | 0.97 (0.95, 0.99) | 0.38 |
|  |  | Imported | 17 | 2660/2720 | 61.23 | 0.00 | 0.99 (0.98, 0.99) |  |
|  | Cirrhosis status | Non-cirrhotic | 13 | 1385/1416 |  |  |  |  |
|  |  | Locally developed | 4 | 601/611 | 77.57 | 0.00 | 0.98 (0.95, 1.00) | 0.66 |
|  |  | Imported | 9 | 784/805 | 0.00 | 0.52 | 0.98 (0.97, 0.99) |  |
|  |  | Mixed | 49 | 5905/6179 |  |  |  |  |
|  |  | Locally developed | 3 | 734/758 | / | / | 0.96 (0.92, 0.99) | 0.98 |
|  |  | Imported | 46 | 5171/5421 | 78.52 | 0.00 | 0.97 (0.95, 0.98) |  |
|  | Treatment experience | Treatment-naïve | 14 | 1421/1491 |  |  |  |  |
|  |  | Locally developed | 4 | 294/310 | 62.64 | 0.05 | 0.96 (0.91, 0.99) | 0.72 |
|  |  | Imported | 10 | 1127/1181 | 66.21 | 0.00 | 0.97 (0.94, 0.99) |  |
|  |  | Mixed | 46 | 8549/9498 |  |  |  |  |
|  |  | Locally developed | 3 | 1041/1059 | / | / | 0.99 (0.96, 0.998) | 0.10 |
|  |  | Imported | 43 | 7508/8439 | 96.70 | 0.00 | 0.96 (0.94, 0.99) |  |
|  | Pan-genotypic | Yes | 43 | 6898/7907 | 96.39 | 0.00 | 0.95 (0.92, 0.97) |  |
|  |  | Locally developed | 2 | 680/697 | / | / | 0.98 (0.96, 0.99) | **0.02** |
|  |  | Imported | 41 | 6218/7210 | 96.29 | 0.00 | 0.95 (0.91, 0.97) |  |
|  |  | No | 39 | 4933/5081 | 59.23 | 0.00 | 0.98 (0.97, 0.98) |  |
|  |  | Locally developed | 5 | 655/672 | 84.61 | 0.00 | 0.97 (0.92, 0.999) | 0.87 |
|  |  | Imported | 34 | 4278/4409 | 48.90 | 0.00 | 0.98 (0.97, 0.99) |  |
|  |  | Genotype subgroup difference: *P*-for-interaction = 0.07, *I*^2^ = 94.81% | | | | | | |
| Relapse | Ethnicity | Asian | 21 | 57/3770 |  |  |  |  |
|  |  | Locally developed | 7 | 28/1369 | 69.52 | 0.003 | 0.02 (0.006, 0.04) | 0.14 |
|  |  | Imported | 14 | 29/2401 | 32.87 | 0.11 | 0.006 (0.001, 0.01) |  |
|  | Cirrhosis status | Non-cirrhotic | 13 | 20/1416 |  |  |  |  |
|  |  | Locally developed | 4 | 8/611 | 68.67 | 0.02 | 0.01 (< 1E-09, 0.04) | 0.96 |
|  |  | Imported | 9 | 12/805 | 14.59 | 0.31 | 0.009 (0.002, 0.02) |  |
|  |  | Mixed | 45 | 176/5529 |  |  |  |  |
|  |  | Locally developed | 3 | 20/758 | / | / | 0.03 (0.009, 0.06) | 0.74 |
|  |  | Imported | 42 | 156/4771 | 75.21 | 0.00 | 0.02 (0.01, 0.03) |  |
|  | Treatment experience | Treatment-naïve | 13 | 48/1461 |  |  |  |  |
|  |  | Locally developed | 4 | 12/310 | 42.06 | 0.16 | 0.03 (0.008, 0.07) | 0.52 |
|  |  | Imported | 9 | 36/1151 | 77.74 | 0.00002 | 0.02 (0.003, 0.05) |  |
|  |  | Mixed | 41 | 144/6448 |  |  |  |  |
|  |  | Locally developed | 3 | 16/1059 | / | / | 0.01 (0.002, 0.03) | 0.45 |
|  |  | Imported | 38 | 128/5389 | 75.95 | 0.00 | 0.02 (0.008, 0.03) |  |
|  | Pan-genotypic | Yes | 41 | 208/5367 | 83.73 | 0.00 | 0.03 (0.01, 0.04) |  |
|  |  | Locally developed | 2 | 15/697 | / | / | 0.02 (0.01, 0.03) | 0.30 |
|  |  | Imported | 39 | 193/4670 | 84.32 | 0.00 | 0.03 (0.01, 0.04) |  |
|  |  | No | 30 | 77/4078 | 57.34 | 0.00006 | 0.01 (0.007, 0.02) |  |
|  |  | Locally developed | 5 | 13/672 | 77.62 | 0.001 | 0.02 (0.0009, 0.06) | 0.70 |
|  |  | Imported | 25 | 64/3406 | 51.95 | 0.001 | 0.01 (0.006, 0.02) |  |
|  |  | Genotype subgroup difference: *P*-for-interaction = 0.23, *I^2^* = 78.93% | | | | | | |
| Virological breakthrough | Ethnicity | Asian | 17 | 14/2465 |  |  |  |  |
|  |  | Locally developed | 7 | 13/1369 | 79.00 | 0.00007 | 0.003 (< 1E-09, 0.02) | 0.43 |
|  |  | Imported | 10 | 1/1096 | 0.00 | 0.99 | < 1E-09 (< 1E-09, 0.002) |  |
|  | Cirrhosis status | Non-cirrhotic | 11 | 0/1259 |  |  |  |  |
|  |  | Locally developed | 4 | 0/611 | 0.00 | 0.89 | < 1E-09 (< 1E-09, 0.001) | 0.68 |
|  |  | Imported | 7 | 0/648 | 0.00 | 0.99 | < 1E-09 (< 1E-09, 0.001) |  |
|  |  | Mixed | 36 | 20/4712 |  |  |  |  |
|  |  | Locally developed | 3 | 13/758 | / | / | 0.01 (< 1E-09, 0.06) | 0.31 |
|  |  | Imported | 33 | 7/3954 | 0.00 | 0.999 | < 1E-09 (< 1E-09, 0.0008) |  |
|  | Treatment experience | Treatment-naïve | 11 | 1/1304 |  |  |  |  |
|  |  | Locally developed | 4 | 1/310 | 0.00 | 0.53 | 0.0003 (< 1E-09, 0.0096) | 0.28 |
|  |  | Imported | 7 | 0/994 | 0.00 | 0.97 | < 1E-09 (< 1E-09, 0.000009) |  |
|  |  | Mixed | 33 | 21/4552 |  |  |  |  |
|  |  | Locally developed | 3 | 12/1059 | / | / | 0.006 (< 1E-09, 0.04) | 0.70 |
|  |  | Imported | 30 | 9/3493 | 0.00 | 0.95 | 0.00004 (< 1E-09, 0.001) |  |
|  | Pan-genotypic | Yes | 32 | 24/4387 | 37.86 | 0.02 | 0.0007 (< 1E-09, 0.003) |  |
|  |  | Locally developed | 2 | 12/697 | / | / | 0.009 (0.003, 0.02) | **0.04** |
|  |  | Imported | 30 | 12/3690 | 0.00 | 0.67 | 0.00006 (< 1E-09, 0.001) |  |
|  |  | No | 22 | 3/2620 | 0.00 | 0.998 | < 1E-09 (< 1E-09, 0.0004) |  |
|  |  | Locally developed | 5 | 1/672 | 0.00 | 0.45 | < 1E-09 (< 1E-09, 0.002) | 0.78 |
|  |  | Imported | 17 | 2/1948 | 0.00 | 0.9998 | < 1E-09 (< 1E-09, 0.0008) |  |
|  |  | Genotype subgroup difference: *P-*for-interaction = 0.18, *I^2^* = 97.21% | | | | | | |

**Notes:** *SATs* single-arm trials; *DAAs* direct-acting antiviral agents; *ES* effect size; *CI* confidence interval; *SVR12* 12-week sustained virologic response, which is undetectable HCV RNA in the blood 12 weeks after the end of treatment; *P*-value for test of group difference; Heterogeneity is not reported in case that the number of trials ≤ 3.

Table S10: Subgroup analyses of SATs for safety outcome measures: locally developed vs imported DAAs

| Outcome measure | Subgroup | Origin of DAA | No. of included studies | No. of events/sample size | Heterogeneity | | ES (95% *CI*) | *P*-value |
| --- | --- | --- | --- | --- | --- | --- | --- | --- |
|  |  |  |  |  | *I^2^*(%) | *P* |  |  |
| AEs | Ethnicity | Asian | 20 | 2478/3735 |  |  |  |  |
|  |  | Locally developed | 7 | 1150/1369 | 96.01 | 0.00 | 0.93 (0.85, 0.99) | **<0.0001** |
|  |  | Imported | 13 | 1328/2366 | 92.96 | 0.00 | 0.54 (0.46, 0.63) |  |
|  | Cirrhosis status | Non-cirrhotic | 12 | 968/1304 |  |  |  |  |
|  |  | Locally developed | 4 | 528/611 | 97.47 | 0.00 | 0.98 (0.81, 1.00) | **0.03** |
|  |  | Imported | 8 | 440/693 | 96.19 | 0.00 | 0.72 (0.53, 0.88) |  |
|  |  | Mixed | 41 | 3479/5035 |  |  |  |  |
|  |  | Locally developed | 3 | 622/758 | / | / | 0.85 (0.77, 0.92) | **0.002** |
|  |  | Imported | 38 | 2857/4277 | 95.46 | 0.00 | 0.69 (0.62, 0.75) |  |
|  | Treatment experience | Treatment-naïve | 13 | 990/1379 |  |  |  |  |
|  |  | Locally developed | 4 | 307/310 | 57.53 | 0.07 | 0.996 (0.97, 1.00) | **0.0001** |
|  |  | Imported | 9 | 683/1069 | 98.11 | 0.00 | 0.69 (0.45, 0.88) |  |
|  |  | Mixed | 38 | 3863/5991 |  |  |  |  |
|  |  | Locally developed | 3 | 843/1059 | / | / | 0.80 (0.76, 0.83) | **<0.0001** |
|  |  | Imported | 35 | 3020/4932 | 92.87 | 0.00 | 0.64 (0.58, 0.69) |  |
|  | Pan-genotypic | Yes | 35 | 3028/4472 | 96.36 | 0.00 | 0.72 (0.64, 0.79) |  |
|  |  | Locally developed | 2 | 564/697 | / | / | 0.81 (0.78, 0.84) | **0.01** |
|  |  | Imported | 33 | 2464/3775 | 96.33 | 0.00 | 0.71 (0.63, 0.79) |  |
|  |  | No | 28 | 2604/3858 | 95.89 | 0.00 | 0.73 (0.65, 0.80) |  |
|  |  | Locally developed | 5 | 586/672 | 96.67 | 0.00 | 0.97 (0.84, 1.00) | **0.0003** |
|  |  | Imported | 23 | 2018/3186 | 92.37 | 0.00 | 0.66 (0.59, 0.72) |  |
|  |  | Genotype subgroup difference: *P*-for-interaction = 0.81, *I^2^* = 96.10% | | | | | | |
| SAEs | Ethnicity | Asian | 21 | 92/2810 |  |  |  |  |
|  |  | Locally developed | 7 | 63/1369 | 34.76 | 0.16 | 0.04 (0.03, 0.06) | **0.003** |
|  |  | Imported | 14 | 29/1441 | 10.82 | 0.33 | 0.01 (0.008, 0.02) |  |
|  | Cirrhosis status | Non-cirrhotic | 12 | 38/1371 |  |  |  |  |
|  |  | Locally developed | 4 | 23/611 | 0.00 | 0.75 | 0.03 (0.02, 0.05) | 0.09 |
|  |  | Imported | 8 | 15/760 | 57.30 | 0.02 | 0.01 (0.0009, 0.03) |  |
|  |  | Mixed | 46 | 208/5417 |  |  |  |  |
|  |  | Locally developed | 3 | 40/758 | / | / | 0.05 (0.02, 0.09) | 0.34 |
|  |  | Imported | 43 | 168/4659 | 75.09 | 0.00 | 0.03 (0.02, 0.04) |  |
|  | Treatment experience | Treatment-naïve | 13 | 28/1446 |  |  |  |  |
|  |  | Locally developed | 4 | 10/310 | 0.00 | 0.75 | 0.03 (0.01, 0.05) | 0.05 |
|  |  | Imported | 9 | 18/1136 | 4.58 | 0.40 | 0.009 (0.003, 0.02) |  |
|  |  | Mixed | 42 | 221/5444 |  |  |  |  |
|  |  | Locally developed | 3 | 53/1059 | / | / | 0.05 (0.03, 0.08) | 0.34 |
|  |  | Imported | 39 | 168/4385 | 80.48 | 0.00 | 0.03 (0.02, 0.05) |  |
|  | Pan-genotypic | Yes | 37 | 207/4770 | 80.46 | 0.00 | 0.04 (0.03, 0.05) |  |
|  |  | Locally developed | 2 | 37/697 | / | / | 0.05 (0.04, 0.07) | 0.42 |
|  |  | Imported | 35 | 170/4073 | 80.42 | 0.00 | 0.04 (0.02, 0.05) |  |
|  |  | No | 34 | 77/3350 | 44.51 | 0.003 | 0.01 (0.008, 0.02) |  |
|  |  | Locally developed | 5 | 26/672 | 0.00 | 0.82 | 0.04 (0.02, 0.05) | **0.007** |
|  |  | Imported | 29 | 51/2678 | 41.85 | 0.01 | 0.01 (0.005, 0.02) |  |
|  |  | Genotype subgroup difference: *P*-for-interaction = 0.005, *I^2^* = 73.06% | | | | | | |

**Notes:** *SATs* single-arm trials; *DAAs* direct-acting antiviral agents; *ES* effect size; *CI* confidence interval; *AEs* any adverse events; *SAEs* serious adverse events; *P*-value for test of group difference; Bold means statistically significant; Heterogeneity is not reported in case that the number of trials ≤ 3.

**Figure S4**: Forest plot for SVR12 rate from SATs: locally developed vs imported DAAs

**
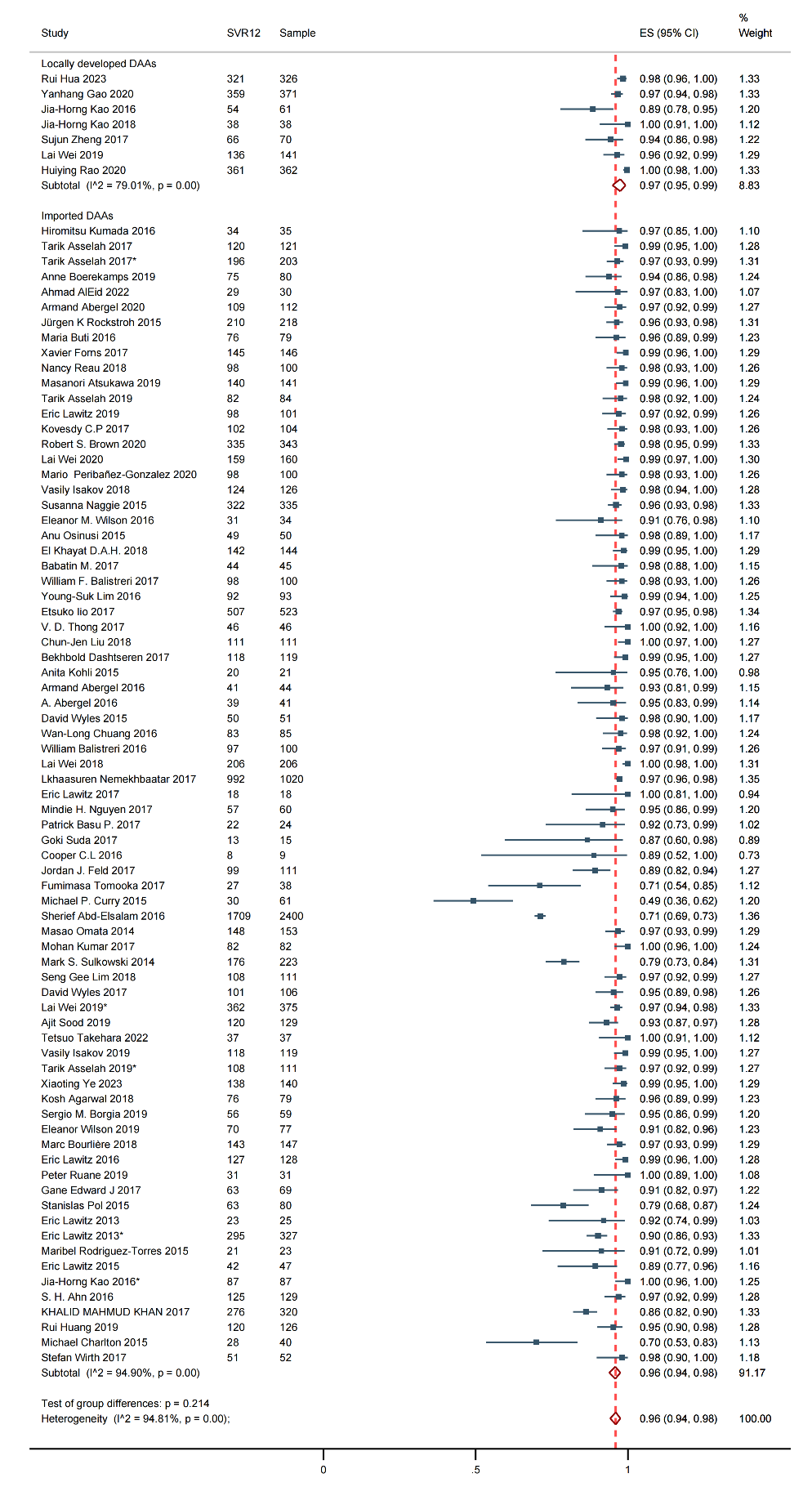
**

**Notes:** *SVR12* 12-week sustained virologic response, which is undetectable HCV RNA in the blood 12 weeks after the end of treatment; *SATs* single-arm trials; *DAAs* direct-acting antiviral agents; *ES* effect size.

Figure S5: Forest plot for relapse rate from SATs: locally developed vs imported DAAs


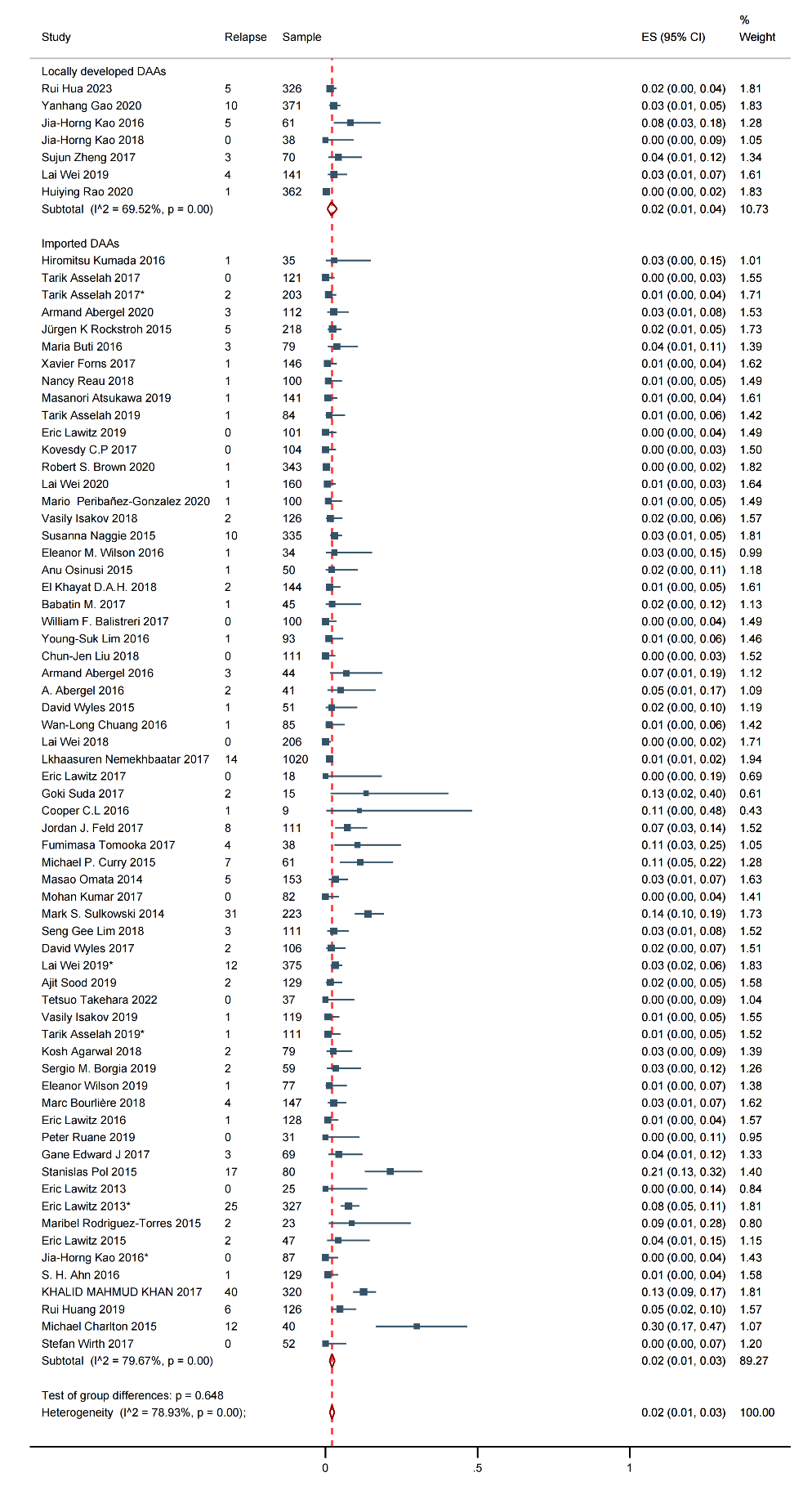


**Notes:** *SATs* single-arm trials; *DAAs* direct-acting antiviral agents; *ES* effect size.

Figure S6: Forest plot for virological breakthrough rate from SATs: locally developed vs imported DAAs


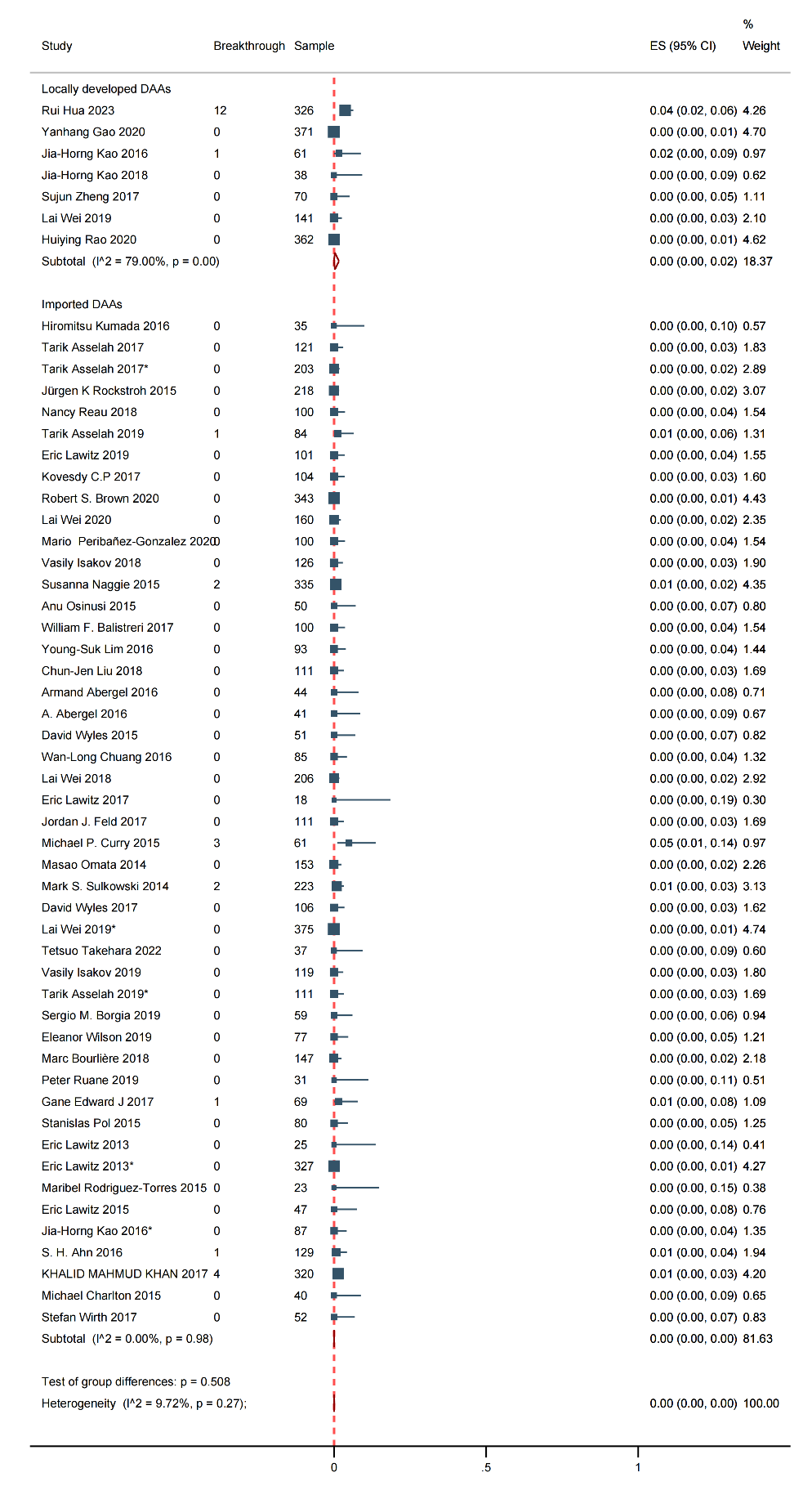


**Notes:** *SATs* single-arm trials; *DAAs* direct-acting antiviral agents; *ES* effect size.

Figure S7: Forest plot for AE rate from SATs: locally developed vs imported DAAs


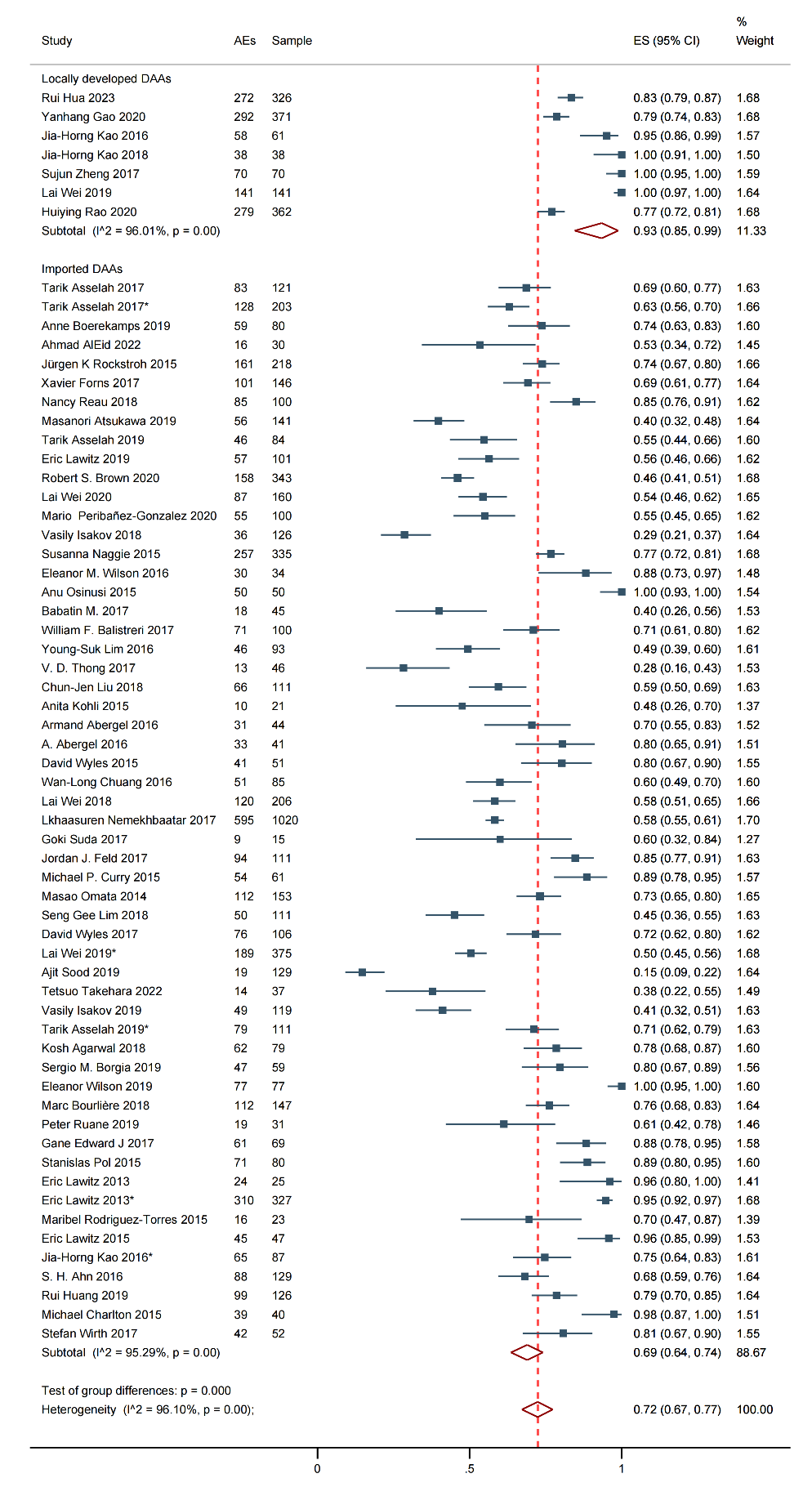


**Notes:** *AEs* any adverse events; *SATs* single-arm trials; *DAAs* direct-acting antiviral agents; *ES* effect size.

Figure S8: Forest plot for SAE rate from SATs: locally developed vs imported DAAs

**
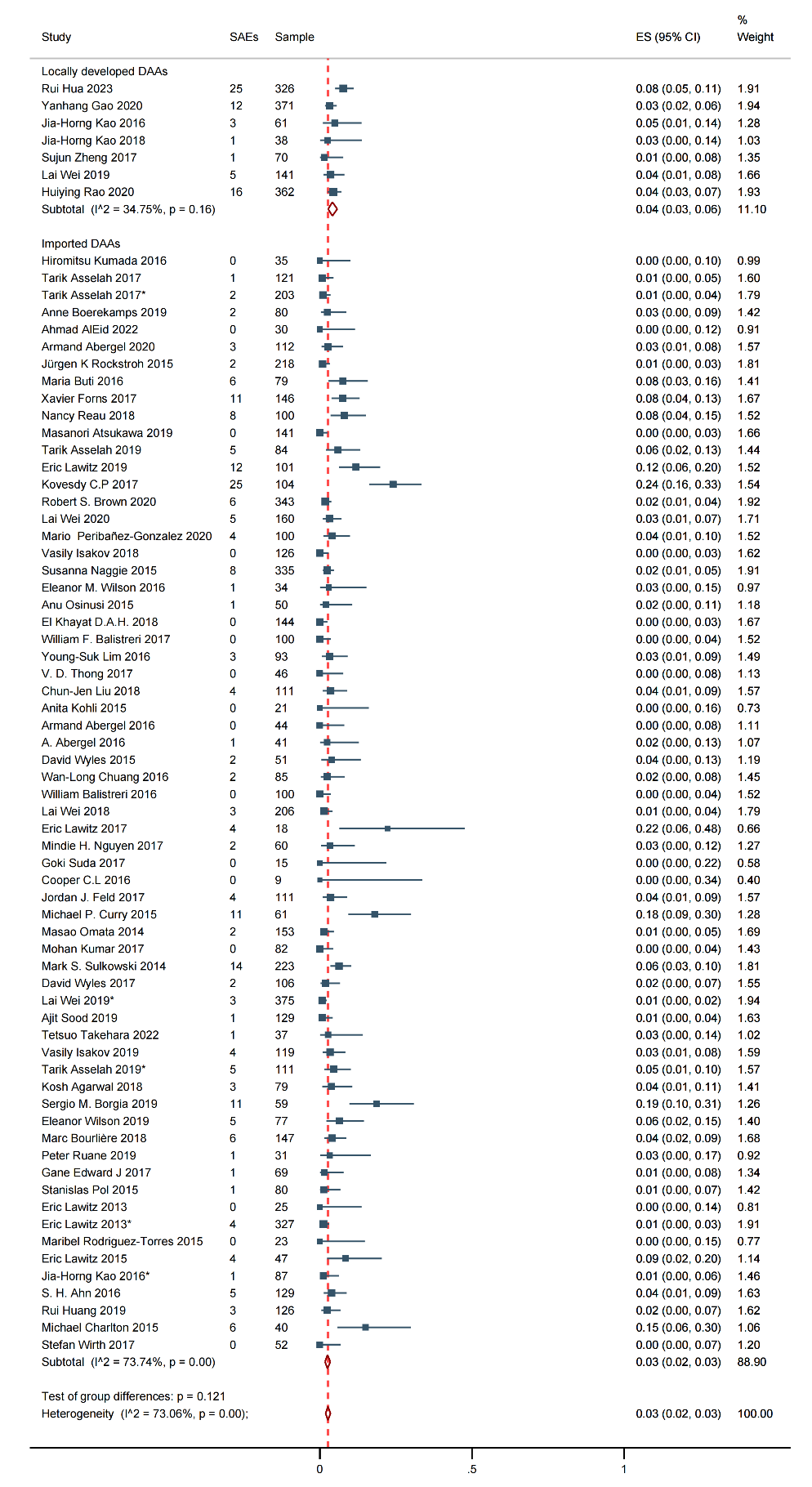
**

**Notes:** *SAEs* serious adverse events; *SATs* single-arm trials; *DAAs* direct-acting antiviral agents; *ES* effect size.

Figure S9: Forest plot for SVR12 rate from SATs: pan-genotypic vs genotype-specific DAAs

**
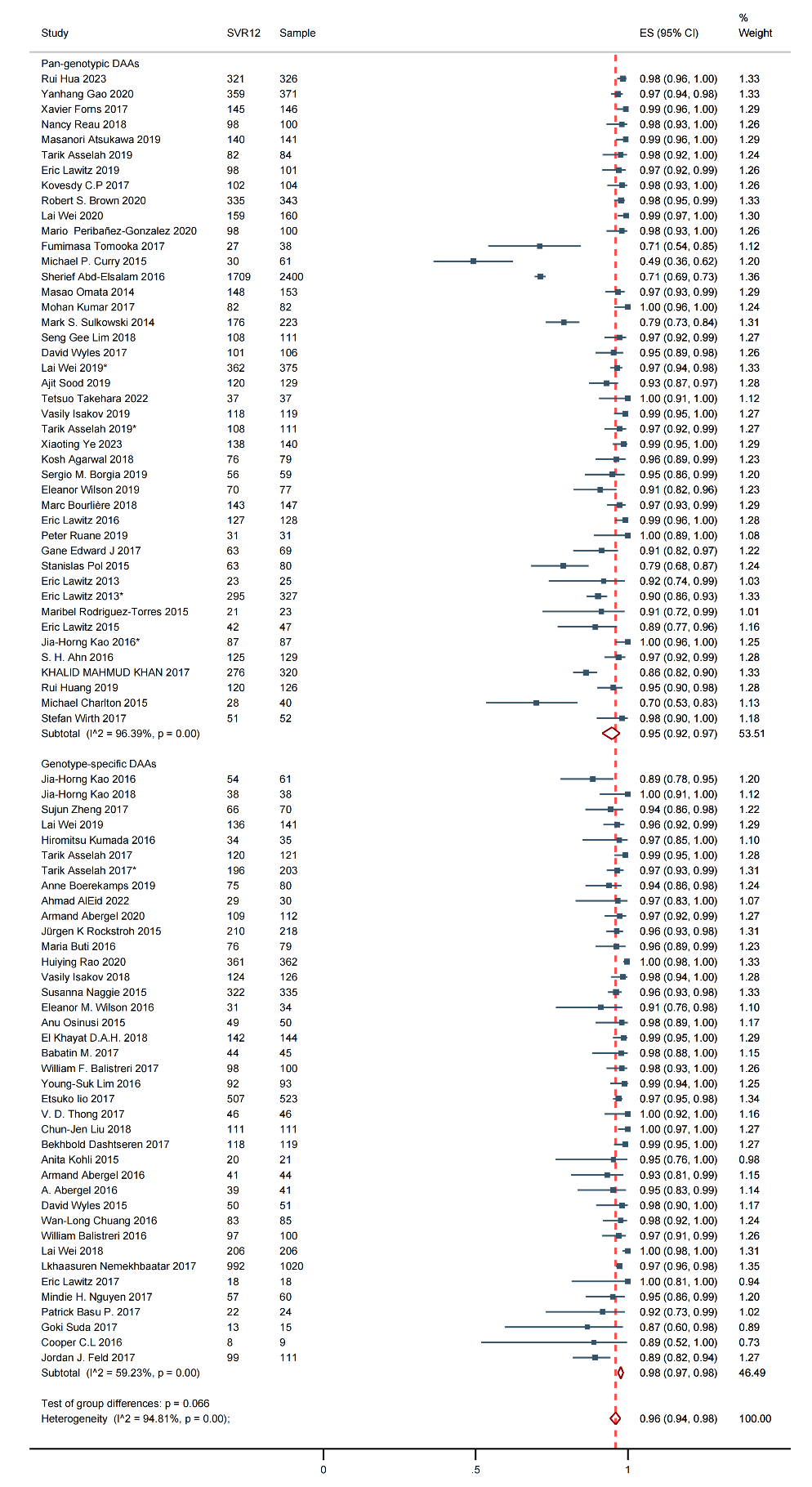
**

**Notes:** *SVR12* 12-week sustained virologic response, which is undetectable HCV RNA in the blood 12 weeks after the end of treatment; *SATs* single-arm trials; *DAAs* direct-acting antiviral agents; *ES* effect size.

Figure S10: Forest plot for relapse rate from SATs: pan-genotypic vs genotype-specific DAAs


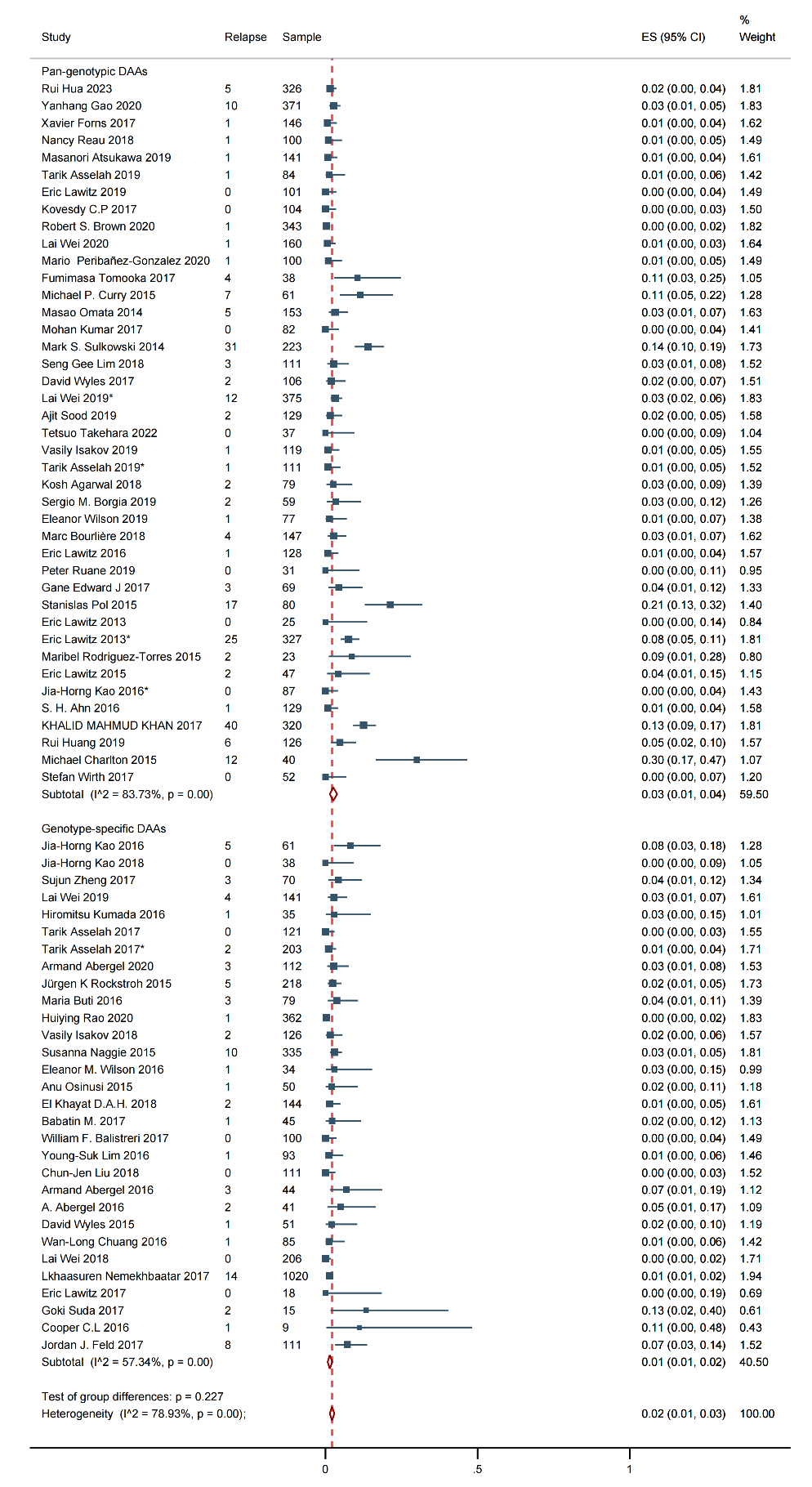


**Notes:** *SATs* single-arm trials; *DAAs* direct-acting antiviral agents; *ES* effect size.

Figure S11: Forest plot for virological breakthrough rate from SATs: pan-genotypic vs genotype-specific DAAs


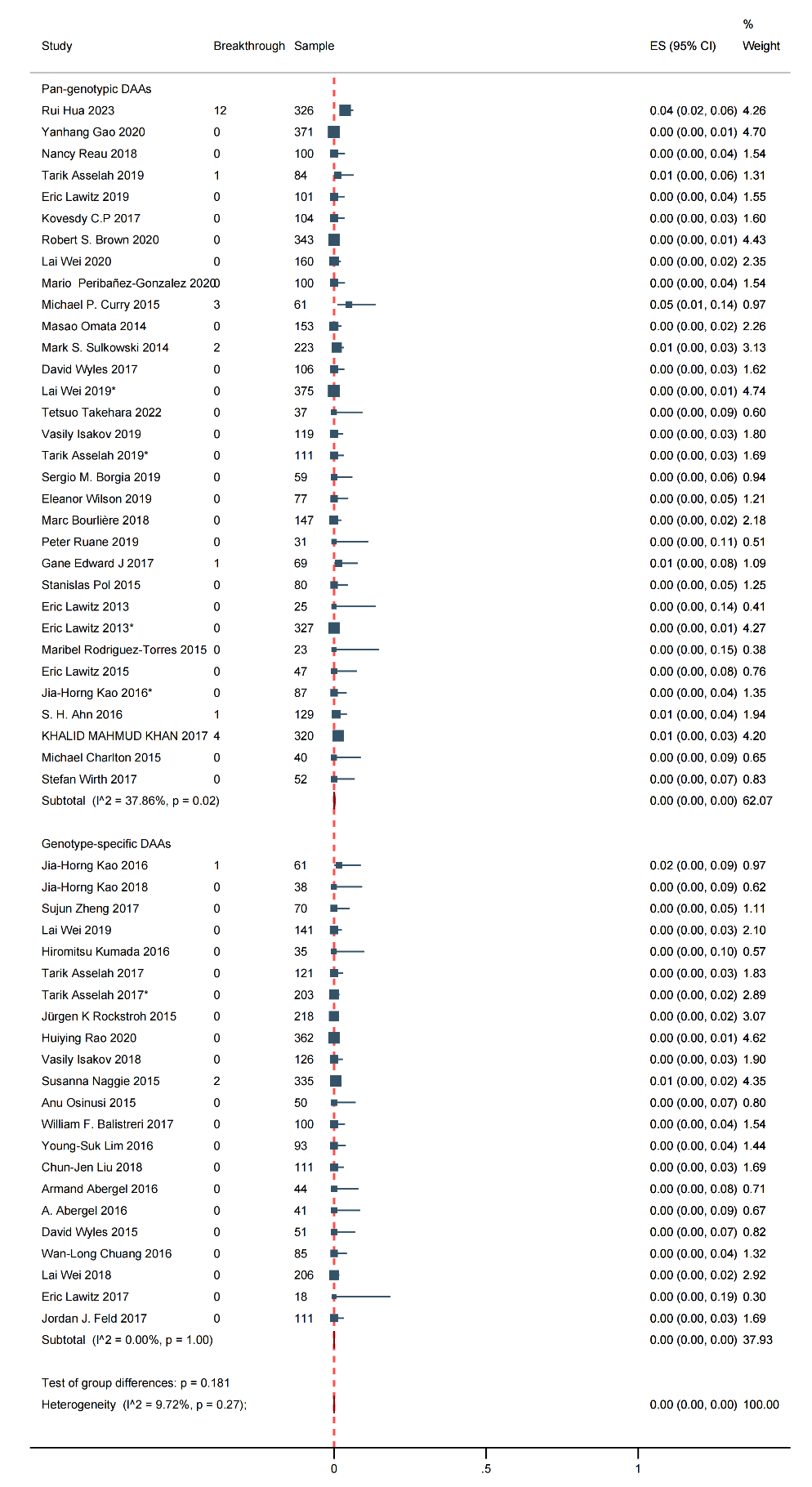


**Notes:** *SATs* single-arm trials; *DAAs* direct-acting antiviral agents; *ES* effect size.

Figure S12: Forest plot for AE rate from SATs: pan-genotypic vs genotype-specific DAAs


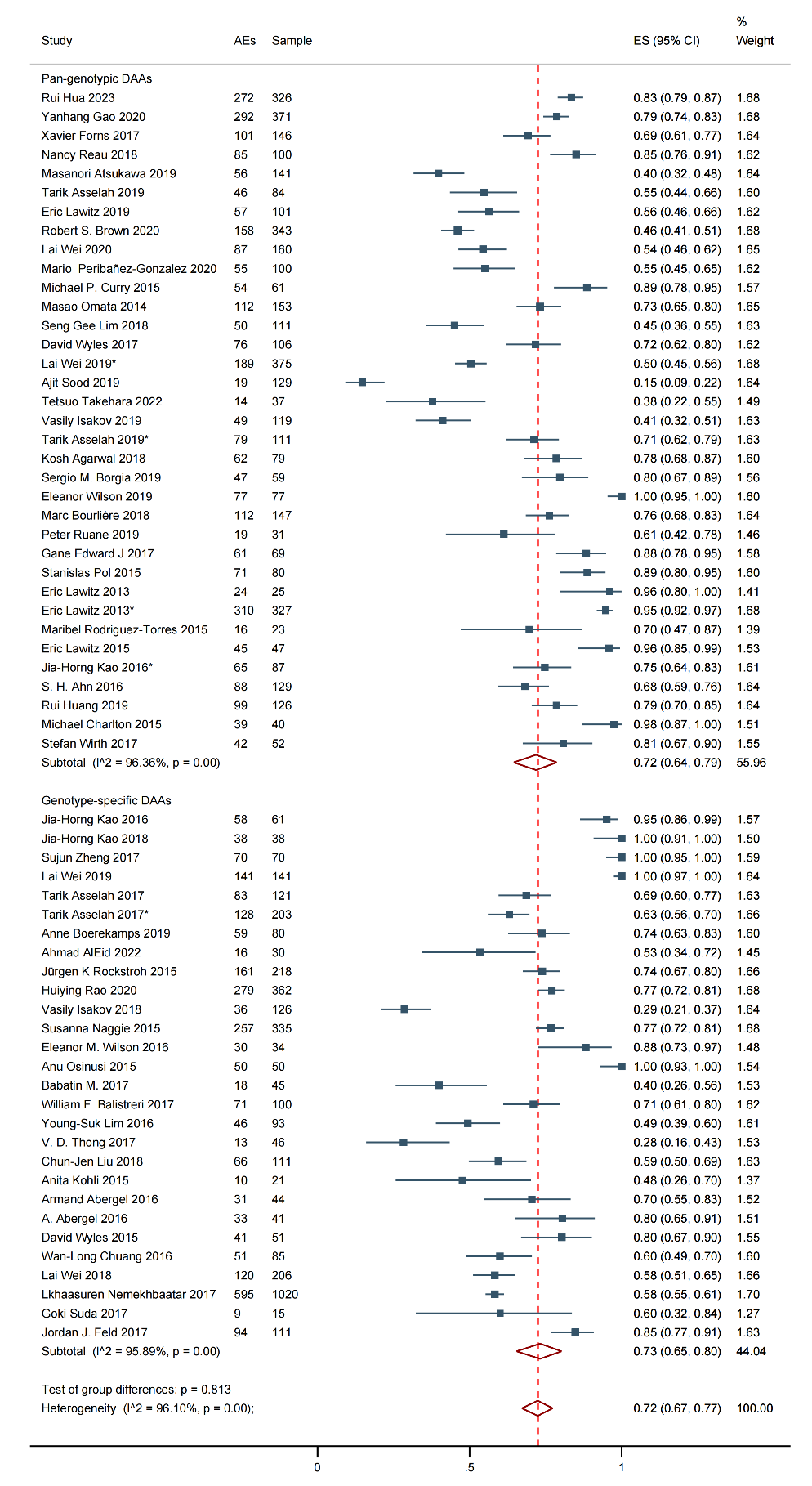


**Notes:** *AEs* any adverse events; *SATs* single-arm trials; *DAAs* direct-acting antiviral agents; *ES* effect size.

Figure S13: Forest plot for SAE rate from SATs: pan-genotypic vs genotype-specific DAAs


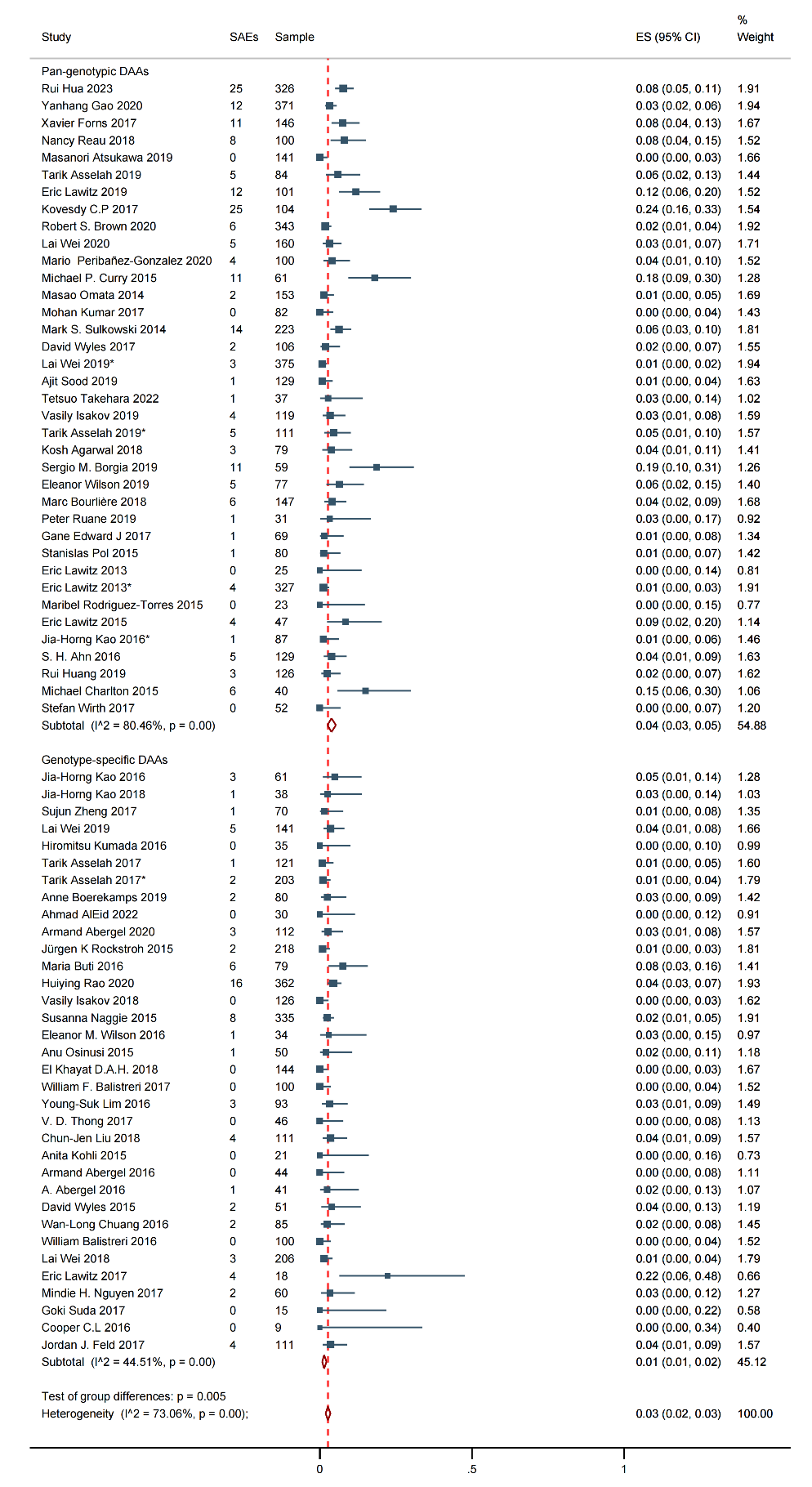


**Notes:** *SAEs* serious adverse events; *SATs* single-arm trials; *DAAs* direct-acting antiviral agents; *ES* effect size.

Table S11: Univariate meta-regression of SATs for efficacy and safety outcome measures

| Outcome measure | Variable | No. of included studies | Coefficient | 95% *CI* | *I^2^* (%) | Adjusted *R^2^* (%) | *P* |
| --- | --- | --- | --- | --- | --- | --- | --- |
| SVR12 | Origin of DAA | 82 |  |  | 45.79 | 4.65 |  |
|  | Imported (Ref.) | 75 |  |  |  |  |  |
|  | Locally developed | 7 | 0.028 | -0.061, 0.116 |  |  | 0.54 |
|  | Mean age | 65 | -0.001 | -0.004, 0.002 | 55.22 | -0.90 | 0.59 |
|  | Ethnicity | 82 |  |  | 20.62 | 68.55 |  |
|  | Asian (Ref.) | 24 |  |  |  |  |  |
|  | White and black | 42 | -0.039 | -0.091, 0.013 |  |  | 0.14 |
|  | Unknown | 16 | -0.080 | -0.139, -0.020 |  |  | **0.01** |
|  | Cirrhosis status | 82 |  |  | 0.00 | 100.00 |  |
|  | All non-cirrhotic (Ref.) | 13 |  |  |  |  |  |
|  | All cirrhotic | 8 | -0.207 | -0.270, -0.143 |  |  | **< 0.001** |
|  | Mixed | 49 | -0.022 | -0.081, 0.036 |  |  | 0.45 |
|  | Unknown | 12 | -0.031 | -0.099, 0.036 |  |  | 0.36 |
|  | Treatment experience | 82 |  |  | 47.74 | -3.16 |  |
|  | All naïve (Ref.) | 14 |  |  |  |  |  |
|  | All experienced | 11 | -0.034 | -0.153, 0.085 |  |  | 0.57 |
|  | Mixed | 46 | -0.008 | -0.088, 0.073 |  |  | 0.85 |
|  | Unknown | 11 | -0.017 | -0.121, 0.087 |  |  | 0.74 |
|  | Pan-genotypic | 82 |  |  | 35.43 | 36.89 |  |
|  | Yes (Ref.) | 43 |  |  |  |  |  |
|  | No | 39 | 0.044 | -0.007, 0.094 |  |  | 0.09 |
| Relapse | Origin of DAA | 71 |  |  | 0.00 | / |  |
|  | Imported (Ref.) | 64 |  |  |  |  |  |
|  | Locally developed | 7 | -0.011 | -0.070, 0.047 |  |  | 0.70 |
|  | Mean age | 57 | 0.0002 | -0.002, 0.003 | 0.00 | / | 0.86 |
|  | Ethnicity | 71 |  |  | 0.00 | / |  |
|  | Asian (Ref.) | 21 |  |  |  |  |  |
|  | White and black | 40 | 0.022 | -0.023, 0.067 |  |  | 0.33 |
|  | Unknown | 10 | 0.034 | -0.028, 0.095 |  |  | 0.28 |
|  | Cirrhosis status | 71 |  |  | 0.00 | / |  |
|  | All non-cirrhotic (Ref.) | 13 |  |  |  |  |  |
|  | All cirrhotic | 7 | 0.0006 | -0.087, 0.088 |  |  | 0.99 |
|  | Mixed | 45 | 0.018 | -0.042, 0.077 |  |  | 0.55 |
|  | Unknown | 6 | 0.032 | -0.040, 0.104 |  |  | 0.38 |
|  | Treatment experience | 71 |  |  | 0.00 | / |  |
|  | All naïve (Ref.) | 13 |  |  |  |  |  |
|  | All experienced | 11 | 0.022 | -0.072, 0.116 |  |  | 0.64 |
|  | Mixed | 41 | -0.011 | -0.068, 0.047 |  |  | 0.72 |
|  | Unknown | 6 | 0.032 | -0.053, 0.116 |  |  | 0.46 |
|  | Pan-genotypic | 71 |  |  | 0.00 | / |  |
|  | Yes (Ref.) | 41 |  |  |  |  |  |
|  | No | 30 | 0.020 | -0.061, 0.022 |  |  | 0.34 |
| Virological breakthrough | Origin of DAA | 54 |  |  | 0.00 | / |  |
|  | Imported (Ref.) | 47 |  |  |  |  |  |
|  | Locally developed | 7 | 0.007 | -0.053, 0.067 |  |  | 0.82 |
|  | Mean age | 46 | 0.00009 | -0.003, 0.003 | 0.00 | / | 0.95 |
|  | Ethnicity | 54 |  |  | 0.00 | / |  |
|  | Asian (Ref.) | 17 |  |  |  |  |  |
|  | White and black | 34 | -0.003 | -0.055, 0.049 |  |  | 0.90 |
|  | Unknown | 3 | -0.0007 | -0.082, 0.081 |  |  | 0.99 |
|  | Cirrhosis status | 54 |  |  | 0.00 | / |  |
|  | All non-cirrhotic (Ref.) | 11 |  |  |  |  |  |
|  | All cirrhotic | 5 | 0.005 | -0.093, 0.102 |  |  | 0.92 |
|  | Mixed | 36 | 0.004 | -0.059, 0.068 |  |  | 0.89 |
|  | Unknown | 2 | 0.010 | -0.105, 0.125 |  |  | 0.86 |
|  | Treatment experience | 54 |  |  | 0.00 | / |  |
|  | All naïve (Ref.) | 11 |  |  |  |  |  |
|  | All experienced | 7 | 0.001 | -0.104, 0.106 |  |  | 0.98 |
|  | Mixed | 33 | 0.004 | -0.059, 0.067 |  |  | 0.90 |
|  | Unknown | 3 | 0.005 | -0.091, 0.102 |  |  | 0.91 |
|  | Pan-genotypic | 54 |  |  | 0.00 | / |  |
|  | Yes (Ref.) | 32 |  |  |  |  |  |
|  | No | 22 | -0.004 | -0.054, 0.045 |  |  | 0.86 |
| AEs | Origin of DAA | 63 |  |  | 72.86 | 17.85 |  |
|  | Imported (Ref.) | 56 |  |  |  |  |  |
|  | Locally developed | 7 | 0.223 | 0.082, 0.365 |  |  | **0.003** |
|  | Mean age | 55 | -0.001 | -0.007, 0.005 | 75.39 | -1.83 | 0.71 |
|  | Ethnicity | 63 |  |  | 75.52 | 4.07 |  |
|  | Asian (Ref.) | 20 |  |  |  |  |  |
|  | White and black | 37 | 0.065 | -0.039, 0.170 |  |  | 0.22 |
|  | Unknown | 6 | -0.162 | -0.342, 0.017 |  |  | 0.08 |
|  | Cirrhosis status | 63 |  |  | 76.22 | -7.03 |  |
|  | All non-cirrhotic (Ref.) | 12 |  |  |  |  |  |
|  | All cirrhotic | 6 | -0.136 | -0.338, 0.067 |  |  | 0.19 |
|  | Mixed | 41 | -0.078 | -0.211, 0.056 |  |  | 0.25 |
|  | Unknown | 4 | -0.073 | -0.305, 0.159 |  |  | 0.53 |
|  | Treatment experience | 63 |  |  | 75.58 | 5.93 |  |
|  | All naïve (Ref.) | 13 |  |  |  |  |  |
|  | All experienced | 9 | 0.098 | -0.080, 0.276 |  |  | 0.28 |
|  | Mixed | 38 | -0.099 | -0.225, 0.026 |  |  | 0.12 |
|  | Unknown | 3 | 0.031 | -0.205, 0.267 |  |  | 0.79 |
|  | Pan-genotypic | 63 |  |  | 77.32 | -4.48 |  |
|  | Yes (Ref.) | 35 |  |  |  |  |  |
|  | No | 28 | 0.008 | -0.094, 0.110 |  |  | 0.88 |
| SAEs | Origin | 71 |  |  | 0.00 | / |  |
|  | Imported (Ref.) | 64 |  |  |  |  |  |
|  | Locally developed | 7 | 0.013 | -0.046, 0.072 |  |  | 0.66 |
|  | Mean age | 61 | 0.0009 | -0.001, 0.003 | 0.00 | / | 0.40 |
|  | Ethnicity | 71 |  |  | 0.00 | / |  |
|  | Asian (Ref.) | 21 |  |  |  |  |  |
|  | White and black | 41 | 0.005 | -0.044, 0.054 |  |  | 0.84 |
|  | Unknown | 9 | -0.002 | -0.073, 0.068 |  |  | 0.95 |
|  | Cirrhosis status | 71 |  |  | 0.00 | / |  |
|  | All non-cirrhotic (Ref.) | 12 |  |  |  |  |  |
|  | All cirrhotic | 7 | 0.015 | -0.073, 0.103 |  |  | 0.73 |
|  | Mixed | 46 | 0.011 | -0.049, 0.071 |  |  | 0.72 |
|  | Unknown | 6 | -0.022 | -0.125, 0.081 |  |  | 0.67 |
|  | Treatment experience | 71 |  |  | 0.00 | / |  |
|  | All naïve (Ref.) | 13 |  |  |  |  |  |
|  | All experienced | 11 | 0.023 | -0.072, 0.117 |  |  | 0.63 |
|  | Mixed | 42 | 0.021 | -0.038, 0.080 |  |  | 0.47 |
|  | Unknown | 5 | -0.006 | -0.103, 0.091 |  |  | 0.91 |
|  | Pan-genotypic | 71 |  |  | 0.00 | / |  |
|  | Yes (Ref.) | 37 |  |  |  |  |  |
|  | No | 34 | -0.020 | -0.065, 0.024 |  |  | 0.37 |

**Notes:** *SATs* single-arm trials; *CI* confidence interval; *SVR12* 12-week sustained virologic response, which is undetectable HCV RNA in the blood 12 weeks after the end of treatment; *AEs* any adverse events; *SAEs* serious adverse events; Bold means statistically significant.

﹒

Table S12: Multiple meta-regression of SATs for efficacy outcome measures

| Outcome measure | Variable | Coefficient | | 95% *CI* | *P* |
| --- | --- | --- | --- | --- | --- |
| SVR12 | Origin of DAAs |  | |  |  |
|  | Imported (Ref.) |  | |  |  |
|  | Locally developed | -0.037 | | -0.124, 0.050 | 0.40 |
|  | Ethnicity |  | |  |  |
|  | Asian (Ref.) |  | |  |  |
|  | White and black | -0.049 | | -0.110, 0.012 | 0.11 |
|  | Unknown | -0.076 | | -0.139, -0.012 | **0.02** |
|  | Cirrhosis status |  | |  |  |
|  | All non-cirrhotic (Ref.) |  | |  |  |
|  | All cirrhotic | -0.138 | | -0.227, -0.049 | **0.003** |
|  | Mixed | -0.027 | | -0.096, 0.043 | 0.45 |
|  | Unknown | -0.046 | | -0.136, 0.044 | 0.31 |
|  | No. of included studies = 82; *I^2^* = 0.00%; Adjusted *R^2^* = 55.65% | | | | |
| AEs | Origin of DAAs | |  |  |  |
|  | Imported (Ref.) | |  |  |  |
|  | Locally developed | | 0.337 | 0.188, 0.486 | **< 0.001** |
|  | Ethnicity | |  |  |  |
|  | Asian (Ref.) | |  |  |  |
|  | White and black | | 0.185 | 0.080, 0.291 | **0.001** |
|  | Unknown | | -0.036 | -0.202, 0.130 | 0.67 |
|  | No. of included studies = 63; *I*^2^ = 66.74%; Adjusted *R*^2^ = 35.39% | | | | |
| SAEs | Origin of DAAs | |  |  |  |
|  | Imported (Ref.) | |  |  |  |
|  | Locally developed | | 0.015 | -0.044, 0.074 | 0.61 |
|  | Pan-genotypic | |  |  |  |
|  | Yes (Ref.) | |  |  |  |
|  | No | | -0.021 | -0.066, 0.024 | 0.35 |
|  | No. of included studies = 71; *I^2^* = 0.00%; Adjusted *R^2^* = NA | | | | |

**Notes:** *SATs* single-arm trials; *CI* confidence interval; *Ref.* reference; *SVR12* 12-week sustained virologic response, which is undetectable HCV RNA in the blood 12 weeks after the end of treatment**;** *AEs* any adverse events; *SAEs* serious adverse events**;** Bold means statistically significant.

Figure S14: Publication bias for RD of AEs from RCTs

*P*-value of Egger test = 0.61, *N* = 15

Notes: *RD* risk difference; *AEs* any adverse events; *RCTs* randomized controlled trials; *DAAs* direct-acting antiviral agents.

Figure S15: Publication bias for RD of AEs from RCTs with placebo as the control

*P*-value of Egger test = 0.26, *N* = 11

Notes: *RD* risk difference; *AEs* any adverse events; *RCTs* randomized controlled trials; *DAAs* direct-acting antiviral agents.

Figure S16: Publication bias for RD of AEs from RCTs with placebo as the control of imported DAAs

*P*-value of Egger test = 0.81, *N* = 10

Notes: *RD* risk difference; *AEs* any adverse events; *RCTs* randomized controlled trials; *DAAs* direct-acting antiviral agents.

Figure S17: Publication bias for RD of SAEs from RCTs

*P*-value of Egger test = 0.15, *N* = 17

Notes: *RD* risk difference; *SAEs* serious adverse events; *RCTs* randomized controlled trials; DAAs direct-acting antiviral agents.

Figure S18: Publication bias for RD of SAEs from RCTs with placebo as the control

*P*-value of Egger test = 0.053, *N* = 13

Notes: *RD* risk difference; *SAEs* serious adverse events; *RCTs* randomized controlled trials; *DAAs* direct-acting antiviral agents.

Figure S19: Publication bias for RD of SAEs from RCTs with placebo as the control of imported DAAs

*P*-value of Egger test = 0.09, *N* = 12

Notes: *RD* risk difference; *SAEs* serious adverse events; *RCTs* randomized controlled trials; *DAAs* direct-acting antiviral agents.

Figure S20: Publication bias for SVR12 rate from SATs

*P*-value of Egger test < **0.001**, *N* = 82

**Notes:** *SVR12* 12-week sustained virologic response, which is undetectable HCV RNA in the blood 12 weeks after the end of treatment; *SATs* single-arm trials; *DAAs* direct-acting antiviral agents; *ES* effect size; *se(ES)* standard error for effect size.

Figure S21: Publication bias for SVR12 rate from SATs of imported DAAs

*P*-value of Egger test < **0.001**, N = 75

**Notes:** *SVR12* 12-week sustained virologic response, which is undetectable HCV RNA in the blood 12 weeks after the end of treatment; *SATs* single-arm trials; *DAAs* direct-acting antiviral agents; *ES* effect size; *se(ES)* standard error for effect size.

Figure S22: Publication bias for relapse rate from SATs

*P*-value of Egger test = 0.33, *N* = 71

**Notes:** *SATs* single-arm trials; *DAAs* direct-acting antiviral agents; *ES* effect size; *se(ES)* standard error for effect size.

Figure S23: Publication bias for relapse rate from SATs of imported DAAs

*P*-value of Egger test = 0.50, *N* = 64

**Notes:** *SATs* single-arm trials; *DAAs* direct-acting antiviral agents; *ES* effect size; *se(ES)* standard error for effect size.

Figure S24: Publication bias for virological breakthrough rate from SATs

*P*-value of Egger test = 0.40, *N* = 54

**Notes:** *SATs* single-arm trials; *DAAs* direct-acting antiviral agents; *ES* effect size; *se(ES)* standard error for effect size.

Figure S25: Publication bias for virological breakthrough rate from SATs of imported DAAs

*P*-value of Egger test = 0.28, *N* = 47

**Notes:** *SATs* single-arm trials; *DAAs* direct-acting antiviral agents; *ES* effect size; *se(ES)* standard error for effect size.

Figure S26: Publication bias for AE rate from SATs

*P*-value of Egger test = 0.28, *N* = 63

Notes: *AEs* any adverse events; *SATs* single-arm trials; *DAAs* direct-acting antiviral agents; *ES* effect size; *se(ES)* standard error for effect size.

Figure S27: Publication bias for AE rate from SATs of imported DAAs

*P*-value of Egger test = 0.17, *N* = 56

Notes: *AEs* any adverse events; *SATs* single-arm trials; *DAAs* direct-acting antiviral agents; *ES* effect size; *se(ES)* standard error for effect size.

Figure S28: Publication bias for SAE rate from SATs

*P*-value of Egger test = 0.29, *N* = 71

**Notes:** *SAEs* serious adverse events; *SATs* single-arm trials; *DAAs* direct-acting antiviral agents; *ES* effect size; *se(ES)* standard error for effect size.

Figure S29: Publication bias for SAE rate from SATs of imported DAAs

*P*-value of Egger test = 0.10, *N* = 64

**Notes:** *SAEs* serious adverse events; *SATs* single-arm trials; *DAAs* direct-acting antiviral agents; *ES* effect size; *se(ES)* standard error for effect size.

Table S13: Sensitivity analyses of univariate meta-regressions of SATs (removed ‘some concerns’)

| Outcome measure | Variable | No. of included studies | Coefficient | 95% CI | *I^2^* (%) | Adjusted *R^2^* (%) | *P* |
| --- | --- | --- | --- | --- | --- | --- | --- |
| SVR12 | Origin of DAA | 71 |  |  | 48.52 | 6.97 |  |
|  | Imported (Ref.) | 64 |  |  |  |  |  |
|  | Locally developed | 7 | 0.029 | -0.066, 0.123 |  |  | 0.55 |
|  | Mean age | 60 | -0.0003 | -0.004, 0.003 | 57.95 | -3.30 | 0.84 |
|  | Ethnicity | 71 |  |  | 22.18 | 69.26 |  |
|  | Asian (Ref.) | 21 |  |  |  |  |  |
|  | White and black | 41 | -0.040 | -0.097, 0.017 |  |  | 0.16 |
|  | Unknown | 9 | -0.084 | -0.156, -0.013 |  |  | **0.02** |
|  | Cirrhosis | 71 |  |  | 0.00 | 100.00 |  |
|  | All non-cirrhosis (Ref.) | 12 |  |  |  |  |  |
|  | All cirrhosis | 8 | -0.207 | -0.271, -0.143 |  |  | **<0.0001** |
|  | Mixed | 48 | -0.023 | -0.083, 0.036 |  |  | 0.44 |
|  | Unknown | 3 | -0.099 | -0.253, 0.055 |  |  | 0.20 |
|  | Treatment experience | 71 |  |  | 50.97 | -2.33 |  |
|  | All naïve (Ref.) | 13 |  |  |  |  |  |
|  | All experienced | 11 | -0.033 | -0.158, 0.091 |  |  | 0.59 |
|  | Mixed | 44 | -0.008 | -0.094, 0.078 |  |  | 0.85 |
|  | Unknown | 3 | -0.020 | -0.182, 0.142 |  |  | 0.81 |
|  | Pan-genotypic | 71 |  |  | 41.30 | 29.78 |  |
|  | Yes (Ref.) | 38 |  |  |  |  |  |
|  | No | 33 | 0.040 | -0.019, 0.098 |  |  | 0.18 |
| Relapse | Origin of DAA | 64 |  |  | 0.00 | / |  |
|  | Imported (Ref.) | 57 |  |  |  |  |  |
|  | Locally developed | 7 | -0.011 | -0.070, 0.049 |  |  | 0.73 |
|  | Mean age | 54 | 0.00004 | -0.003, 0.003 | 0.00 | / | 0.98 |
|  | Ethnicity | 64 |  |  | 0.00 | / |  |
|  | Asian (Ref.) | 20 |  |  |  |  |  |
|  | White and black | 39 | 0.023 | -0.027, 0.072 |  |  | 0.36 |
|  | Unknown | 5 | 0.013 | -0.066, 0.092 |  |  | 0.74 |
|  | Cirrhosis | 64 |  |  | 0.00 | / |  |
|  | All non-cirrhosis (Ref.) | 12 |  |  |  |  |  |
|  | All cirrhosis | 7 | 0.0009 | -0.087, 0.089 |  |  | 0.98 |
|  | Mixed | 44 | 0.019 | -0.042, 0.079 |  |  | 0.54 |
|  | Unknown | 1 | 0.199 | -0.031, 0.428 |  |  | 0.09 |
|  | Treatment experience | 64 |  |  | 0.00 | / |  |
|  | All naïve (Ref.) | 12 |  |  |  |  |  |
|  | All experienced | 11 | 0.022 | -0.073, 0.117 |  |  | 0.65 |
|  | Mixed | 39 | -0.009 | -0.069, 0.051 |  |  | 0.77 |
|  | Unknown | 2 | 0.006 | -0.116, 0.129 |  |  | 0.92 |
|  | Pan-genotypic | 64 |  |  | 0.00 | / |  |
|  | Yes (Ref.) | 37 |  |  |  |  |  |
|  | No | 27 | -0.013 | -0.060, 0.034 |  |  | 0.58 |
| Virological breakthrough | Origin of DAA | 53 |  |  | 0.00 | / |  |
|  | Imported (Ref.) | 46 |  |  |  |  |  |
|  | Locally developed | 7 | 0.008 | -0.053, 0.068 |  |  | 0.80 |
|  | Mean age | 47 | 0.00009 | -0.003, 0.003 | 0.00 | / | 0.95 |
|  | Ethnicity | 53 |  |  | 0.00 | / |  |
|  | Asian (Ref.) | 17 |  |  |  |  |  |
|  | White and black | 34 | -0.003 | -0.055, 0.049 |  |  | 0.90 |
|  | Unknown | 2 | -0.006 | -0.106, 0.095 |  |  | 0.91 |
|  | Cirrhosis | 53 |  |  | 0.00 | / |  |
|  | All non-cirrhosis (Ref.) | 11 |  |  |  |  |  |
|  | All cirrhosis | 5 | 0.005 | -0.093, 0.102 |  |  | 0.92 |
|  | Mixed | 36 | 0.004 | -0.059, 0.068 |  |  | 0.89 |
|  | Unknown | 1 | -2e-18 | -0.231, 0.231 |  |  | >0.999 |
|  | Treatment experience | 53 |  |  | 0.00 | / |  |
|  | All naïve (Ref.) | 11 |  |  |  |  |  |
|  | All experienced | 7 | 0.001 | -0.104, 0.106 |  |  | 0.98 |
|  | Mixed | 33 | 0.004 | -0.059, 0.067 |  |  | 0.90 |
|  | Unknown | 2 | -0.0008 | -0.125, 0.123 |  |  | 0.99 |
|  | Pan-genotypic | 53 |  |  | 0.00 | / |  |
|  | Yes (Ref.) | 31 |  |  |  |  |  |
|  | No | 22 | -0.004 | -0.054, 0.046 |  |  | 0.88 |
| AEs | Origin of DAA | 61 |  |  | 72.87 | 15.56 |  |
|  | Imported (Ref.) | 54 |  |  |  |  |  |
|  | Locally developed | 7 | 0.218 | 0.074, 0.362 |  |  | **0.004** |
|  | Mean age | 54 | -0.001 | -0.007, 0.004 | 75.34 | -1.60 | 0.61 |
|  | Ethnicity | 61 |  |  | 75.35 | 3.12 |  |
|  | Asian (Ref.) | 19 |  |  |  |  |  |
|  | White and black | 37 | 0.060 | -0.050, 0.170 |  |  | 0.28 |
|  | Unknown | 5 | -0.152 | -0.349, 0.045 |  |  | 0.13 |
|  | Cirrhosis | 61 |  |  | 75.96 | 0.91 |  |
|  | All non-cirrhosis (Ref.) | 11 |  |  |  |  |  |
|  | All cirrhosis | 6 | -0.163 | -0.366, 0.040 |  |  | 0.11 |
|  | Mixed | 41 | -0.105 | -0.241, 0.032 |  |  | 0.13 |
|  | Unknown | 3 | -0.045 | -0.318, 0.228 |  |  | 0.74 |
|  | Treatment experience | 61 |  |  | 75.45 | 3.87 |  |
|  | All naïve (Ref.) | 12 |  |  |  |  |  |
|  | All experienced | 9 | 0.072 | -0.113, 0.258 |  |  | 0.44 |
|  | Mixed | 37 | -0.122 | -0.256, 0.011 |  |  | 0.07 |
|  | Unknown | 3 | 0.006 | -0.239, 0.252 |  |  | 0.96 |
|  | Pan-genotypic | 61 |  |  | 76.67 | -2.11 |  |
|  | Yes (Ref.) | 35 |  |  |  |  |  |
|  | No | 26 | 0.023 | -0.081, 0.128 |  |  | 0.66 |
| SAEs | Origin of DAA | 68 |  |  | 0.00 | / |  |
|  | Imported (Ref.) | 61 |  |  |  |  |  |
|  | Locally developed | 7 | 0.012 | -0.048, 0.071 |  |  | 0.70 |
|  | Mean age | 59 | 0.0009 | -0.002, 0.004 | 0.00 | / | 0.52 |
|  | Ethnicity | 68 |  |  | 0.00 | / |  |
|  | Asian (Ref.) | 21 |  |  |  |  |  |
|  | White and black | 41 | 0.005 | -0.044, 0.054 |  |  | 0.84 |
|  | Unknown | 6 | 0.010 | -0.070, 0.090 |  |  | 0.80 |
|  | Cirrhosis | 68 |  |  | 0.00 | / |  |
|  | All non-cirrhosis (Ref.) | 12 |  |  |  |  |  |
|  | All cirrhosis | 7 | 0.015 | -0.073, 0.103 |  |  | 0.73 |
|  | Mixed | 46 | 0.011 | -0.050, 0.071 |  |  | 0.72 |
|  | Unknown | 3 | -0.012 | -0.166, 0.142 |  |  | 0.88 |
|  | Treatment experience | 68 |  |  | 0.00 | / |  |
|  | All naïve (Ref.) | 13 |  |  |  |  |  |
|  | All experienced | 11 | 0.023 | -0.072, 0.117 |  |  | 0.63 |
|  | Mixed | 41 | 0.022 | -0.037, 0.082 |  |  | 0.45 |
|  | Unknown | 3 | 0.0002 | -0.111, 0.112 |  |  | 0.997 |
|  | Pan-genotypic | 68 |  |  | 0.00 | / |  |
|  | Yes (Ref.) | 36 |  |  |  |  |  |
|  | No | 32 | -0.019 | -0.065, 0.027 |  |  | 0.40 |

**Notes:** *SATs* single-arm trials; *CI* confidence interval; *Ref.* reference; *SVR12* 12-week sustained virologic response, which is undetectable HCV RNA in the blood 12 weeks after the end of treatment**;** *AEs* any adverse events; *SAEs* serious adverse events**;** Bold means statistically significant.

Table S14: Sensitivity analyses of multiple meta-regressions of SATs (removed ‘some concerns’)

| Outcome measure | Variable | | Coefficient | | 95% CI | | *P* |
| --- | --- | --- | --- | --- | --- | --- | --- |
| SVR12 | Origin of DAAs | |  | |  | |  |
|  | Imported (Ref.) | |  | |  | |  |
|  | Locally developed | | -0.036 | | -0.129, 0.058 | | 0.45 |
|  | Ethnicity | |  | |  | |  |
|  | Asian (Ref.) | |  | |  | |  |
|  | White and black | | -0.048 | | -0.117, 0.020 | | 0.16 |
|  | Unknown | | -0.078 | | -0.157, 0.0007 | | 0.052 |
|  | Cirrhosis status | |  | |  | |  |
|  | All non-cirrhotic (Ref.) | |  | |  | |  |
|  | All cirrhotic | | -0.026 | | -0.098, 0.047 | | 0.48 |
|  | Mixed | | -0.131 | | -0.224, -0.038 | | **0.007** |
|  | Unknown | | -0.089 | | -0.256, 0.078 | | 0.29 |
|  | No. of included studies = 71; *I^2^* = 0.00%; Adjusted *R^2^* = 51.50% | | | | | | |
| AEs | Origin of DAAs |  | | |  | |  |
|  | Imported (Ref.) |  | | |  | |  |
|  | Locally developed | 0.235 | | | 0.091, 0.379 | | **0.002** |
|  | Treatment experience |  | | |  | |  |
|  | All naïve (Ref.) |  | | |  | |  |
|  | All experienced | 0.155 | | | -0.022, 0.332 | | 0.09 |
|  | Mixed | -0.066 | | | -0.192, 0.060 | | 0.30 |
|  | Unknown | 0.087 | | | -0.140, 0.314 | | 0.45 |
|  | No. of included studies = 61; *I*^2^ = 69.49%; Adjusted *R*^2^ = 27.00% | | | | | | |
| SAEs | Origin of DAAs |  | |  | |  | |
|  | Imported (Ref.) |  | |  | |  | |
|  | Locally developed | 0.014 | | -0.046, 0.073 | | 0.64 | |
|  | Pan-genotypic |  | |  | |  | |
|  | Yes (Ref.) |  | |  | |  | |
|  | No | -0.020 | | -0.067, 0.026 | | 0.38 | |
|  | No. of included studies = 68; *I*^2^ = 0.00%; Adjusted *R*^2^ = / | | | | | | |

**Notes:** *SATs* single-arm trials; *CI* confidence interval; *Ref.* reference; *SVR12* 12-week sustained virologic response, which is undetectable HCV RNA in the blood 12 weeks after the end of treatment**;** *AEs* any adverse events; Bold means statistically significant.

**Table S15:** The Preferred Reporting Items for Systematic Reviews and Meta-Analyses for Protocols

| **Section and Topic** | **Item #** | **Checklist item** | **Reported on Page #** |
| --- | --- | --- | --- |
| **TITLE** | | |  |
| Title | 1 | Identify the report as a systematic review. | 1 |
| **ABSTRACT** | | |  |
| Abstract | 2 | See the PRISMA 2020 for Abstracts checklist. | 1–2 |
| **INTRODUCTION** | | |  |
| Rationale | 3 | Describe the rationale for the review in the context of existing knowledge. | 2–3 |
| Objectives | 4 | Provide an explicit statement of the objective(s) or question(s) the review addresses. | 3–4 |
| **METHODS** | | |  |
| Eligibility criteria | 5 | Specify the inclusion and exclusion criteria for the review and how studies were grouped for the syntheses. | 4–5 |
| Information sources | 6 | Specify all databases, registers, websites, organisations, reference lists and other sources searched or consulted to identify studies. Specify the date when each source was last searched or consulted. | 4–5 |
| Search strategy | 7 | Present the full search strategies for all databases, registers and websites, including any filters and limits used. | 4 |
| Selection process | 8 | Specify the methods used to decide whether a study met the inclusion criteria of the review, including how many reviewers screened each record and each report retrieved, whether they worked independently, and if applicable, details of automation tools used in the process. | 4–5 |
| Data collection process | 9 | Specify the methods used to collect data from reports, including how many reviewers collected data from each report, whether they worked independently, any processes for obtaining or confirming data from study investigators, and if applicable, details of automation tools used in the process. | 5 |
| Data items | 10a | List and define all outcomes for which data were sought. Specify whether all results that were compatible with each outcome domain in each study were sought (e.g. for all measures, time points, analyses), and if not, the methods used to decide which results to collect. | 5 |
|  | 10b | List and define all other variables for which data were sought (e.g. participant and intervention characteristics, funding sources). Describe any assumptions made about any missing or unclear information. | 5–6 |
| Study risk of bias assessment | 11 | Specify the methods used to assess risk of bias in the included studies, including details of the tool(s) used, how many reviewers assessed each study and whether they worked independently, and if applicable, details of automation tools used in the process. | 5 |
| Effect measures | 12 | Specify for each outcome the effect measure(s) (e.g. risk ratio, mean difference) used in the synthesis or presentation of results. | 6 |
| Synthesis methods | 13a | Describe the processes used to decide which studies were eligible for each synthesis (e.g. tabulating the study intervention characteristics and comparing against the planned groups for each synthesis (item #5)). | 6 |
|  | 13b | Describe any methods required to prepare the data for presentation or synthesis, such as handling of missing summary statistics, or data conversions. | 6 |
|  | 13c | Describe any methods used to tabulate or visually display results of individual studies and syntheses. | 6 |
|  | 13d | Describe any methods used to synthesize results and provide a rationale for the choice(s). If meta-analysis was performed, describe the model(s), method(s) to identify the presence and extent of statistical heterogeneity, and software package(s) used. | 6 |
|  | 13e | Describe any methods used to explore possible causes of heterogeneity among study results (e.g. subgroup analysis, meta-regression). | 6 |
|  | 13f | Describe any sensitivity analyses conducted to assess robustness of the synthesized results. | 6 |
| Reporting bias assessment | 14 | Describe any methods used to assess risk of bias due to missing results in a synthesis (arising from reporting biases). | 6–7 |
| Certainty assessment | 15 | Describe any methods used to assess certainty (or confidence) in the body of evidence for an outcome. | 6 |
| **RESULTS** | | |  |
| Study selection | 16a | Describe the results of the search and selection process, from the number of records identified in the search to the number of studies included in the review, ideally using a flow diagram. | 7–8 |
|  | 16b | Cite studies that might appear to meet the inclusion criteria, but which were excluded, and explain why they were excluded. | 7–8 |
| Study characteristics | 17 | Cite each included study and present its characteristics. | 8–9 |
| Risk of bias in studies | 18 | Present assessments of risk of bias for each included study. | 9 |
| Results of individual studies | 19 | For all outcomes, present, for each study: (a) summary statistics for each group (where appropriate) and (b) an effect estimate and its precision (e.g. confidence/credible interval), ideally using structured tables or plots. | 9–10, 15–16 |
| Results of syntheses | 20a | For each synthesis, briefly summarise the characteristics and risk of bias among contributing studies. | 9–10, 15–16 |
|  | 20b | Present results of all statistical syntheses conducted. If meta-analysis was done, present for each the summary estimate and its precision (e.g. confidence/credible interval) and measures of statistical heterogeneity. If comparing groups, describe the direction of the effect. | 9–10, 15–16 |
|  | 20c | Present results of all investigations of possible causes of heterogeneity among study results. | 9–10, 15–16 |
|  | 20d | Present results of all sensitivity analyses conducted to assess the robustness of the synthesized results. | 16–17 |
| Reporting biases | 21 | Present assessments of risk of bias due to missing results (arising from reporting biases) for each synthesis assessed. | 17 |
| Certainty of evidence | 22 | Present assessments of certainty (or confidence) in the body of evidence for each outcome assessed. | 16–17 |
| **DISCUSSION** | | |  |
| Discussion | 23a | Provide a general interpretation of the results in the context of other evidence. | 17–22 |
|  | 23b | Discuss any limitations of the evidence included in the review. | 21–22 |
|  | 23c | Discuss any limitations of the review processes used. | 21–22 |
|  | 23d | Discuss implications of the results for practice, policy, and future research. | 22 |
| **OTHER INFORMATION** | | |  |
| Registration and protocol | 24a | Provide registration information for the review, including register name and registration number, or state that the review was not registered. | 2 |
|  | 24b | Indicate where the review protocol can be accessed, or state that a protocol was not prepared. | 2 |
|  | 24c | Describe and explain any amendments to information provided at registration or in the protocol. | 2 |
| Support | 25 | Describe sources of financial or non-financial support for the review, and the role of the funders or sponsors in the review. | 23 |
| Competing interests | 26 | Declare any competing interests of review authors. | 23 |
| Availability of data, code and other materials | 27 | Report which of the following are publicly available and where they can be found: template data collection forms; data extracted from included studies; data used for all analyses; analytic code; any other materials used in the review. | 23 |

List of included studies

1. Everson G. DAUPHINE: a randomized phase II study of danoprevir/ritonavir plus peginterferon alpha-2a/ribavirin in HCV genotypes 1 or 4. Liver Int. 2014;35(1):108–19.

2. Xiaoyuan X, Bo F, Yujuan G, Sujun Z, Jifang S, Xingxiang Y, et al. Efficacy and Safety of All-oral, 12-week Ravidasvir Plus Ritonavir-boosted Danoprevir and Ribavirin in Treatment-naïve Noncirrhotic HCV Genotype 1 Patients: Results from a Phase 2/3 Clinical Trial in China. J Clin Transl Hepatol. 2019;7(3):213–20.

3. Roth D, Nelson DR, Bruchfeld A, Liapakis A, Silva M, Monsour H, et al. Grazoprevir plus elbasvir in treatment-naive and treatment-experienced patients with hepatitis C virus genotype 1 infection and stage 4-5 chronic kidney disease (the C-SURFER study): a combination phase 3 study. Lancet. 2015;386(10003):1537–45.

4. Dore GJ, Altice F, Litwin AH, Dalgard O, Gane EJ, Shibolet O, et al. Elbasvir-Grazoprevir to Treat Hepatitis C Virus Infection in Persons Receiving Opioid Agonist Therapy: a Randomized Trial. Ann Intern Med. 2016;165(9):625–34.

5. Kumada H, Suzuki Y, Karino Y, Chayama K, Kawada N, Okanoue T, et al. The combination of elbasvir and grazoprevir for the treatment of chronic HCV infection in Japanese patients: a randomized phase II/III study. J Gastroenterol. 2016;52(4):520–33.

6. Zeuzem S, Ghalib R, Reddy KR, Pockros PJ, Ben Ari Z, Zhao Y, et al. Grazoprevir-Elbasvir Combination Therapy for Treatment-Naive Cirrhotic and Noncirrhotic Patients With Chronic Hepatitis C Virus Genotype 1, 4, or 6 Infection: a Randomized Trial. Ann Intern Med. 2015;163(1):1‐13.

7. Wei L, Jia JD, Wang FS, Niu JQ, Zhao XM, Mu S, et al. Efficacy and safety of elbasvir/grazoprevir in participants with hepatitis C virus genotype 1, 4, or 6 infection from the Asia-Pacific region and Russia: final results from the randomized C-CORAL study. J Gastroenterol Hepatol. 2019;34(1):12–21.

8. Hézode C, Fried MW, Colombo M, Bourlière M, Spengler U, Ben-Ari Z, et al. Efficacy and safety of elbasvir/grazoprevir in patients with chronic hepatitis c virus infection and inherited blood disorders: Final data from the C-edge IBLD study. Blood. 2016;128(22).

9. Asselah T, Kowdley KV, Zadeikis N, Wang S, Hassanein T, Horsmans Y, et al. Efficacy of Glecaprevir/Pibrentasvir for 8 or 12 Weeks in Patients With Hepatitis C Virus Genotype 2, 4, 5, or 6 Infection Without Cirrhosis. Clin Gastroenterol Hepatol. 2017;16(3):417–26.

10. Wei L, Wang G, Alami NN, Xie W, Heo J, Xie Q, et al. Glecaprevir-pibrentasvir to treat chronic hepatitis C virus infection in Asia: two multicentre, phase 3 studies- a randomised, double-blind study (VOYAGE-1) and an open-label, single-arm study (VOYAGE-2). Lancet Gastroenterology Hepatol. 2020;5(9):839–49.

11. Bourlière M, Bronowicki JP, de Ledinghen V, Hézode C, Zoulim F, Mathurin P, et al. Ledipasvir-sofosbuvir with or without ribavirin to treat patients with HCV genotype 1 infection and cirrhosis non-responsive to previous protease-inhibitor therapy: a randomised, double-blind, phase 2 trial (SIRIUS). Lancet Infect Dis. 2015;15(4):397–404.

12. Lawitz E, Mangia A, Wyles D, Rodriguez-Torres M, Hassanein T, Gordon SC, et al. Sofosbuvir for previously untreated chronic hepatitis C infection. N Engl J Med. 2013;368(20):1878–87.

13. Jacobson IM, Gordon SC, Kowdley KV, Yoshida EM, Rodriguez-Torres M, Sulkowski MS, et al. Sofosbuvir for hepatitis C genotype 2 or 3 in patients without treatment options. N Engl J Med. 2013;368(20):1867–77.

14. Yang Y, Shen L, Wu W, Guo Y, Li H, Liu J, et al. Clinical Efficacy of DAA in the Treatment of HCV Infection and HCV/HIV Co-infection. J Kunming Med Univ. 2020;41(9):107–13. (in Chinese)

15. Lawitz E, Lalezari JP, Hassanein T, Kowdley KV, Poordad FF, Sheikh AM, et al. Sofosbuvir in combination with peginterferon alfa-2a and ribavirin for non-cirrhotic, treatment-naive patients with genotypes 1, 2, and 3 hepatitis C infection: a randomised, double-blind, phase 2 trial. Lancet Infect Dis. 2013;13(5):401–8.

16. Fu X, Jin X, Xu X, Peng Y. Pan-genotype DAA for treatment of chronic hepatitis C. Electron J Emerg Infect Dis. 2018;3(1):30–3. (in Chinese)

17. Huang M, Zhu X. Efficacy and safety analysis of sofosbuvir/velpatasvir combined with interferon α-2b and ribavirin in the treatment of chronic hepatitis C. Jilin Medical J. 2022;43(7):1866–8. (in Chinese)

18. Feld JJ, Jacobson IM, Hézode C, Asselah T, Ruane PJ, Gruener N, et al. Sofosbuvir and Velpatasvir for HCV Genotype 1, 2, 4, 5, and 6 Infection. N Engl J Med. 2015;373(27):2599–607.

19. The New England Journal of MedicineBourlière M, Gordon SC, Flamm SL, Cooper CL, Ramji A, Tong M, et al. Sofosbuvir, Velpatasvir, and Voxilaprevir for Previously Treated HCV Infection. N Engl J Med. 2017;376(22):2134–46.

20. Hua R, Kong F, Li G, Wen X, Zhang Y, Yang X, et al. Alfosbuvir plus Daclatasvir for Treatment of Chronic Hepatitis C Virus Infection in China. Infect Dis Ther. 2023;12(11):2595–609.

21. Gao Y, Kong F, Li G, Li C, Zheng S, Lin J, et al. Coblopasvir and sofosbuvir for treatment of chronic hepatitis C virus infection in China: A single-arm, open-label, phase 3 trial. Liver Int. 2020;40(11):2685–93.

22. Zheng S, Hua R, Xie Q, Feng B, Yang Y, Zhang D, et al. MAKALU: twelve-week of treatment with ritonavir-boosted danoprevir pluzs peginterferon and ribavirin produces 96% SVR12 in HCV genotype 1-infected non-cirrhotic chinese patients. Hepatol Int. 2017;11(1):S190.

23. Wei L, Shang J, Ma Y, Xu X, Huang Y, Guan Y, et al. Efficacy and Safety of 12-week Interferon-based Danoprevir Regimen in Patients with Genotype 1 Chronic Hepatitis C. J Clin Transl Hepatol. 2019;7(3):221–25.

24. Kao JH, Tung SY, Lee Y, Thongsawat S, Tanwandee T, Sheen IS, et al. Ritonavir-boosted danoprevir plus peginterferon alfa-2a and ribavirin in Asian chronic hepatitis C patients with or without cirrhosis. J Gastroenterol Hepatol. 2016;31(10):1757–65.

25. Rao H, Yang X, Tan Y, Ning Q, Yang D, Wang J, et al. Efficacy and Safety of All-oral Emitasvir and Sofosbuvir in Patients with Genotype 1b HCV Infections without Cirrhosis. J Clin Transl Hepatol. 2020;8(3):255–61.

26. Kao J-H, Yu M-L, Chen C-Y, Peng C-Y, Chen M-Y, Tang H, et al. Twelve-week ravidasvir plus ritonavir-boosted danoprevir and ribavirin for non-cirrhotic HCV genotype 1 patients: A phase 2 study. J Gastroenterol Hepatol. 2018;33(8):1507–10.

27. Boerekamps A, De Weggheleire A, van den Berk GE, Lauw FN, Claassen MAA, Posthouwer D, et al. Treatment of acute hepatitis C genotypes 1 and 4 with 8 weeks of grazoprevir plus elbasvir (DAHHS2): an open-label, multicentre, single-arm, phase 3b trial. Lancet Gastroenterology Hepatol. 2019;4(4):269–77.

28. AlEid A, Al Balkhi A, Qutub A, Abbarh S, AlLehibi A, Almtawa A, et al. The efficacy of Elbasvir/Grazoprevir fixed-dose combination for 8 weeks in HCV treatment and health-related quality of life (HRQoL) in treatment-naïve, non-cirrhotic, genotype 4-infected patients (ELEGANT-4): A single-center, single-arm, open-label, phase 3 trial. Saudi J Gastroenterol. 2022;28(3):225–32.

29. Abergel A, Asselah T, Mallat A, Chanteranne B, Faure F, Larrey D, et al. Phase 3, Multicenter Open-Label study to investigate the efficacy of elbasvir and grazoprevir fixed-dose combination for 8 weeks in treatment-naïve, HCV GT1b-infected patients, with non-severe fibrosis. Liver Int. 2020;40(8):1853–9.

30. Rockstroh JK, Nelson M, Katlama C, Lalezari J, Mallolas J, Bloch M, et al. Efficacy and safety of grazoprevir (MK-5172) and elbasvir (MK-8742) in patients with hepatitis C virus and HIV co-infection (C-EDGE CO-INFECTION): A non-randomised, open-label trial. Lancet HIV. 2015;2(8):e319–27.

31. Buti M, Gordon SC, Zuckerman E, Lawitz E, Calleja JL, Hofer H, et al. Grazoprevir, Elbasvir, and Ribavirin for Chronic Hepatitis C Virus Genotype 1 Infection After Failure of Pegylated Interferon and Ribavirin With an Earlier-Generation Protease Inhibitor: Final 24-Week Results From C-SALVAGE. Cini Infect Dis. 2016;62(1):32–6.

32. Forns X, Lee SS, Valdes J, Lens S, Ghalib R, Aguilar H, et al. Glecaprevir plus pibrentasvir for chronic hepatitis C virus genotype 1, 2, 4, 5, or 6 infection in adults with compensated cirrhosis (EXPEDITION-1): a single-arm, open-label, multicentre phase 3 trial. Lancet Infect Dis. 2017;17(10):1062–8.

33. Reau N, Kwo PY, Rhee S, Brown RS, Jr., Agarwal K, Angus P, et al. Glecaprevir/Pibrentasvir Treatment in Liver or Kidney Transplant Patients With Hepatitis C Virus Infection. Hepatology. 2018;68(4):1298–307.

34. Atsukawa M, Tsubota A, Toyoda H, Takaguchi K, Nakamuta M, Watanabe T, et al. The efficacy and safety of glecaprevir plus pibrentasvir in 141 patients with severe renal impairment: a prospective, multicenter study. Aliment Pharmacol Ther. 2019;49(9):1230–41.

35. Asselah T, Lee SS, Yao BB, Nguyen T, Wong F, Mahomed A, et al. Efficacy and safety of glecaprevir/pibrentasvir in patients with chronic hepatitis C virus genotype 5 or 6 infection (ENDURANCE-5,6): an open-label, multicentre, phase 3b trial. Lancet Gastroenterology Hepatol. 2019;4(1):45–51.

36. Lawitz E, Flisiak R, Abunimeh M, Sise ME, Park JY, Kaskas M, et al. Efficacy and safety of glecaprevir/pibrentasvir in renally impaired patients with chronic HCV infection. Liver Int. 2019;40(5):1032–41.

37. Kovesdy CP, Dumas E, Thompson A, Horsmans Y, Reynaert H, Ghali P, et al. Expedition-4: Efficacy and safety of glecaprevir/pibrentasvir in patients with chronic hepatitis C genotype 1-6 infection by dialysis status. J Am Soc Nephrol. 2017;28:9.

38. Brown RS, Jr., Buti M, Rodrigues L, Chulanov V, Chuang W-L, Aguilar H, et al. Glecaprevir/pibrentasvir for 8 weeks in treatment-naive patients with chronic HCV genotypes 1-6 and compensated cirrhosis: The EXPEDITION-8 trial. J Hepatol. 2020;72(3):441–9.

39. Peribanez-Gonzalez M, Cheinquer H, Rodrigues L, Lima MP, Alvares-da-Silva MR, Madruga J, et al. Efficacy and safety of glecaprevir/pibrentasvir in treatment-naïve adults with chronic hepatitis C virus genotypes 1–6 in Brazil. Ann Hepatol. 2020;20:100257.

40. Kao JH, Chien RN, Chang TT, Peng CY, Hu TH, Lo GH, et al. A phase 3b study of sofosbuvir plus ribavirin in Taiwanese patients with chronic genotype 2 hepatitis C virus infection. Liver Int. 2016;36(8):1101–7.

41. Ahn SH, Lim YS, Lee KS, Paik SW, Lee YJ, Jeong SH, et al. A phase 3b study of sofosbuvir plus ribavirin in treatment-naive and treatment-experienced Korean patients chronically infected with genotype 2 hepatitis C virus. J Viral Hepat. 2016;23(5):358–65.

42. Khan KM, Ahmad T, Mozammi R, Khan S, Latif A. Assessment of therapeutic effects of Sofosbuvir plus Ribavirin in patients suffering from hepatitis C virus with genotype 3. Pak J Med Sci. 2017;11(3):1096–9.

43. Huang R, Rao H, Xie Q, Gao Z, Li W, Jiang D, et al. Comparison of the efficacy of sofosbuvir plus ribavirin in Chinese patients with genotype 3a or 3b HCV infection. J Med Virol. 2019;91(7):1313–8.

44. Tomooka F, Nakatani T, Fujimoto Y, Ishida K, Kaya D, Fujinaga Y, et al. Evaluation of IFN-free therapy using sofosbuvir/ribavirin in patients with hepatitis C virus genotype-2 infection. Hepatol Int. 2017;11(1):S1015.

45. Charlton M, Gane E, Manns MP, Brown RS, Jr., Curry MP, Kwo PY, et al. Sofosbuvir and ribavirin for treatment of compensated recurrent hepatitis C virus infection after liver transplantation. Gastroenterology. 2015;148(1):108–17.

46. Curry MP, Forns X, Chung RT, Terrault NA, Brown R, Jr., Fenkel JM, et al. Sofosbuvir and ribavirin prevent recurrence of HCV infection after liver transplantation: an open-label study. Gastroenterology. 2015;148(1):100‐07.e1.

47. Pol S, Sulkowski MS, Hassanein T, Gane EJ, Liu L, Mo H, et al. Sofosbuvir plus pegylated interferon and ribavirin in patients with genotype 1 hepatitis C virus in whom previous therapy with direct-acting antivirals has failed. Hepatology. 2015;62(1):129–34.

48. Abd-Elsalam S, Sharaf-Eldin M, Ahmad Y, Tawefeek S. Sofosbuvir plus ribavirin for treatment of cirrhotic HCV patients genotype-4. United Eur Gastroent. 2016;4(5):A170.

49. Omata M, Nishiguchi S, Ueno Y, Mochizuki H, Izumi N, Ikeda F, et al. Sofosbuvir plus ribavirin in Japanese patients with chronic genotype 2 HCV infection: an open-label, phase 3 trial. J Viral Hepat. 2014;21(11):762–8.

50. Rodriguez-Torres M, Gaggar A, Shen G, Kirby B, Svarovskaia E, Brainard D, et al. Sofosbuvir for chronic hepatitis C virus infection genotype 1-4 in patients coinfected with HIV. J Acquir Immune Defic Syndr. 2015;68(5):543–9.

51. Lawitz E, Poordad F, Brainard DM, Hyland RH, An D, Dvory-Sobol H, et al. Sofosbuvir with peginterferon-ribavirin for 12 weeks in previously treated patients with hepatitis C genotype 2 or 3 and cirrhosis. Hepatology. 2015;61(3):769–75.

52. Wirth S, Rosenthal P, Gonzalez-Peralta RP, Jonas MM, Balistreri WF, Lin CH, et al. Sofosbuvir and ribavirin in adolescents 12-17 years old with hepatitis C virus genotype 2 or 3 infection. Hepatology. 2017;66(4):1102–10.

53. Kumar M, Durrani AA, Yaqoab N, Kumar A, Durrani T. Title the triple thearpy sofosbuvir/ribavirin and peg interferon in asymptomatic hepatitis C geno3 type in northern area of Pakistan. Hepatol Int. 2017;11(1):S1007.

54. Sulkowski MS, Naggie S, Lalezari J, Fessel WJ, Mounzer K, Shuhart M, et al. Sofosbuvir and ribavirin for hepatitis C in patients with HIV coinfection. JAMA. 2014;312(4):353–61.

55. Isakov V, Gankina N, Morozov V, Kersey K, Lu S, Osinusi A, et al. Ledipasvir-Sofosbuvir for 8 Weeks in Non-Cirrhotic Patients with Previously Untreated Genotype 1 HCV Infection ± HIV-1 Co-Infection. Clin Drug Investig. 2018;38(3):239–47.

56. Naggie S, Cooper C, Saag M, Workowski K, Ruane P, Towner WJ, et al. Ledipasvir and Sofosbuvir for HCV in Patients Coinfected with HIV-1. N Engl J Med. 2015;373(8):705–13.

57. Wilson EM, Kattakuzhy S, Sidharthan S, Sims Z, Tang L, McLaughlin M, et al. Successful Retreatment of Chronic HCV Genotype-1 Infection With Ledipasvir and Sofosbuvir After Initial Short Course Therapy With Direct-Acting Antiviral Regimens. Cini Infect Dis. 2016;62(3):280–8.

58. Osinusi A, Townsend K, Kohli A, Nelson A, Seamon C, Meissner EG, et al. Virologic response following combined ledipasvir and sofosbuvir administration in patients with HCV genotype 1 and HIV co-infection. JAMA. 2015;313(12):1232–9.

59. El Khayat DAH, Kamal I, El Sayed M, Elshabrawy M, Ayoub H, Rizk A, et al. Safety of treatment of genotype 4 adolescents chronic HCV infected patients with Ledipasvir/Sofosbuvir combination. Hepatol Int. 2018;12(2):S277.

60. Babatin M, Alghamdi AS, Assiri A, Aleladi H, Shoja H, Mogharbel M, et al. Safety and efficacy of 8-weeks ledipasvir/sofosbuvir combination in chronic hepatitis C genotype 4-infected patients. Hepatology. 2017;66:821A.

61. Balistreri WF, Murray KF, Rosenthal P, Bansal S, Lin CH, Kersey K, et al. The safety and effectiveness of ledipasvir-sofosbuvir in adolescents 12-17 years old with hepatitis C virus genotype 1 infection. Hepatology. 2017;66(2):371–8.

62. Suda G, Ogawa K, Yamamoto Y, Katagiri M, Furuya K, Kumagai K, et al. Retreatment with sofosbuvir, ledipasvir, and add-on ribavirin for patients who failed daclatasvir and asunaprevir combination therapy. J Gastroenterol. 2017;52(10):1122–9.

63. Cooper CL, Naggie S, Saag MS, Yang JC, Stamm LM, Dvory-Sobol H, et al. Retreatment of HCV/HIV-coinfected patients who failed 12 weeks of LDV/SOF. Top Antivir Med. 2016;24(E-1):229.

64. Lim YS, Ahn SH, Lee KS, Paik SW, Lee YJ, Jeong SH, et al. A phase IIIb study of ledipasvir/sofosbuvir fixed-dose combination tablet in treatment-naive and treatment-experienced Korean patients chronically infected with genotype 1 hepatitis C virus. Hepatol Int. 2016;10(6):947–55.

65. Iio E, Shimada N, Takaguchi K, Eguchi Y, Atsukawa M, Tsubota A, et al. Clinical evaluation of sofosbuvir/ledipasvir in chronic hepatitis C genotype 1 with and without prior daclatasvir/asnaprevir therapy at clinical practice. Hepatol Int. 2017;11(1):S1009.

66. Thong VD. Efficacy and safety of ledipasvir/sofosbuvir in treatment-naïve and-experienced patients with hepatitis C virus genotype 6 infection. Hepatology. 2017;66:604A–5A.

67. Liu C-J, Chuang W-L, Sheen IS, Wang H-Y, Chen C-Y, Tseng K-C, et al. Efficacy of Ledipasvir and Sofosbuvir Treatment of HCV Infection in Patients Coinfected With HBV. Gastroenterology. 2018;154(4):989–97.

68. Dashtseren B, Dendev B, Genden Z, Jargalsaikhan G, Oidovsambuu O, Dashdorj N, et al. Hepatitis C treatment with sofosbuvir/ledipasvir single tablet regimen in Mongolia. Hepatol Int. 2017;11(1):S1036–7.

69. Kohli A, Kapoor R, Sims Z, Nelson A, Sidharthan S, Lam B, et al. Ledipasvir and sofosbuvir for hepatitis C genotype 4: a proof-of-concept, single-centre, open-label phase 2a cohort study. Lancet Infect Dis. 2015;15(9):1049–54.

70. Abergel A, Metivier S, Samuel D, Jiang D, Kersey K, Pang PS, et al. Ledipasvir plus sofosbuvir for 12 weeks in patients with hepatitis C genotype 4 infection. Hepatology. 2016;64(4):1049–56.

71. Abergel A, Asselah T, Metivier S, Kersey K, Jiang D, Mo H, et al. Ledipasvir-sofosbuvir in patients with hepatitis C virus genotype 5 infection: an open-label, multicentre, single-arm, phase 2 study. Lancet Infect Dis. 2016;16(4):459–64.

72. Wyles D, Pockros P, Morelli G, Younes Z, Svarovskaia E, Yang JC, et al. Ledipasvir-sofosbuvir plus ribavirin for patients with genotype 1 hepatitis C virus previously treated in clinical trials of sofosbuvir regimens. Hepatology. 2015;61(6):1793–7.

73. Feld JJ, Ramji A, Shafran SD, Willems B, Marotta P, Huchet E, et al. Ledipasvir-Sofosbuvir Plus Ribavirin in Treatment-Naive Patients With Hepatitis C Virus Genotype 3 Infection: An Open-Label Study. Cini Infect Dis. 2017;65(1):13–9.

74. Chuang W, Chien R, Peng C, Chang T, Lo G, Sheen IS, et al. Ledipasvir/sofosbuvir fixed-dose combination tablet in Taiwanese patients with chronic genotype 1 hepatitis C virus. J Gastroenterol Hepatol. 2016;31(7):1323–9.

75. Balistreri W, Rosenthal P, Bansal S, Gonzalez-Peralta R, Wen J, Whitworth MS, et al. Ledipasvir/sofosbuvir for 12 weeks is safe and effective in adolescents with chronic hepatitis C infection. J Pediatr Gastroenterol Nutr. 2016;63:S140–1.

76. Wei L, Xie Q, Hou JL, Tang H, Ning Q, Cheng J, et al. Ledipasvir/sofosbuvir for treatment-naive and treatment-experienced Chinese patients with genotype 1 HCV: an open-label, phase 3b study. Hepatol Int. 2018;12(2):126–32.

77. Nemekhbaatar L, Oidov B, Palam J, Davaadorj T, Damba E, Jenskhan A, et al. Outcome of HCV treatment using ledipasvir/sofosbuvir combination in Mongolian population. Hepatol Int. 2017;11(1):S159.

78. Lawitz E, Landis CS, Maliakkal BJ, Bonacini M, Ortiz-Lasanta G, Zhang J, et al. Safety and efficacy of treatment with once-daily ledipasvir/sofosbuvir (90/400 MG) for 12 weeks in genotype 1 HCV-infected patients with severe renal impairment. Hepatology. 2017;66:848A.

79. Nguyen MH, Trinh H, Do S, Nguyen T. Ledipasvir/sofosbuvir fixed-dose combination (LDV/SOF FDC) for 8 weeks for treatment-Naive, non-cirrhotic hepatitis C genotype 6 (HCV-6) and 12 weeks in those with cirrhosis and/ or prior treatment failure: a multicenter open-labelled clinical trial. Hepatol Int. 2017;11(1):S105.

80. Patrick Basu P, Shah NJ, John N, Aloysius MM, Fortuzi K. Sofosbuvir and ledipasvir in attainment of SVR12 in Sickle Cell Disease (SCD) sub-population with Chronic Hepatitis C (CHC). A single center prospective open label clinical pilot study: slash C trial. Surg Endosc. 2017;31:S260.

81. Lim SG, Mohamed R, Le P, Tee HP, McNabb BL, Lu S, et al. Safety and efficacy of sofosbuvir/velpatasvir in a genotype 1-6 HCV infected population from Singapore, Malaysia, Thailand, and Vietnam: Results from a phase 3, clinical trial. Hepatology. 2018;66:586A.

82. Wyles D, Bräu N, Kottilil S, Daar ES, Ruane P, Workowski K, et al. Sofosbuvir and Velpatasvir for the Treatment of Hepatitis C Virus in Patients Coinfected With Human Immunodeficiency Virus Type 1: An Open-Label, Phase 3 Study. Cini Infect Dis. 2017;65(1):6–12.

83. Wei L, Lim SG, Xie Q, Văn KN, Piratvisuth T, Huang Y, et al. Sofosbuvir-velpatasvir for treatment of chronic hepatitis C virus infection in Asia: a single-arm, open-label, phase 3 trial. Lancet Gastroenterology Hepatol. 2019;4(2):127–34.

84. Sood A, Duseja A, Kabrawala M, Amrose P, Goswami B, Chowdhury A, et al. Sofosbuvir-velpatasvir single-tablet regimen administered for 12 weeks in a phase 3 study with minimal monitoring in India. Hepatol Int. 2019;13(2):173–9.

85. Takehara T, Izumi N, Mochida S, Genda T, Fujiyama S, Notsumata K, et al. Sofosbuvir–velpatasvir in adults with hepatitis C virus infection and compensated cirrhosis in Japan. Hepatol Res. 2022;52(10):833–40.

86. Isakov V, Chulanov V, Abdurakhmanov D, Burnevich E, Nurmukhametova E, Kozhevnikova G, et al. Sofosbuvir/velpatasvir for the treatment of HCV: excellent results from a phase-3, open-label study in Russia and Sweden. Infect Dis Ther. 2019;51(2):131–9.

87. Asselah T, Shafran SD, Bourgeois S, Lai CL, Mathurin P, Willems B, et al. Deferred treatment with a fixed-dose combination of sofosbuvir-velpatasvir for chronic hepatitis C virus genotype 1, 2, 4 and 6 infection. J Viral Hepat. 2019;26(10):1229–32.

88. Gane EJ, Shiffman ML, Etzkorn K, Morelli G, Stedman CA, Davis MN, et al. Sofosbuvir-velpatasvir with ribavirin for 24 weeks in hepatitis C virus patients previously treated with a direct-acting antiviral regimen. Hepatology. 2017;66(4):1083–9.

89. Ye X, Xu S, Hong L. Patients characteristics and the efficacy with sofosbuvir/ velpatasvir of genotype 3 and 6 chronic hepatitis C patients in Eastern China from 2020 to 2021. Hepatol Int. 2023;17:S154.

90. Agarwal K, Castells L, Mullhaupt B, Rosenberg WMC, McNabb B, Arterburn S, et al. Sofosbuvir/velpatasvir for 12 weeks in genotype 1-4 HCV-infected liver transplant recipients. J Hepatol. 2018;69(3):603–7.

91. Borgia SM, Dearden J, Yoshida EM, Shafran SD, Brown A, Ben-Ari Z, et al. Sofosbuvir/velpatasvir for 12 weeks in hepatitis C virus-infected patients with end-stage renal disease undergoing dialysis. J Hepatol. 2019;71(4):660–5.

92. Wilson E, Covert E, Hoffmann J, Comstock E, Emmanuel B, Tang L, et al. A pilot study of safety and efficacy of HCV retreatment with sofosbuvir/velpatasvir/voxilaprevir in patients with or without HIV (RESOLVE STUDY). J Hepatol. 2019;71(3):498–504.

93. Bourliere M, Gordon SC, Schiff ER, Tran TT, Ravendhran N, Landis CS, et al. Deferred treatment with sofosbuvir-velpatasvir-voxilaprevir for patients with chronic hepatitis C virus who were previously treated with an NS5A inhibitor: an open-label substudy of POLARIS-1. Lancet Gastroenterology Hepatol. 2018;3(8):559–65.

94. Lawitz E, Kowdley K, Curry M, Reau N, Nguyen M, Kwo P, et al. High efficacy of sofosbuvir/velpatasvir plus GS-9857 for 12 weeks in treatment-experienced genotype 1-6 HCV-infected patients, including those previously treated with direct-acting antivirals. Am J Gastroenterol. 2016;111:S380.

95. Ruane P, Strasser SI, Gane EJ, Hyland RH, Shao J, Dvory-Sobol H, et al. Sofosbuvir/Velpatasvir/Voxilaprevir for patients with HCV who previously received a Sofosbuvir/Velpatasvir-containing regimen: results from a retreatment study. J Viral Hepat. 2019;26(6):770–3.
